# Anti-Adhesion and Antibiofilm Activity of *Eruca sativa* Miller Extract Targeting Cell Adhesion Proteins of Food-Borne Bacteria as a Potential Mechanism: Combined In Vitro-In Silico Approach

**DOI:** 10.3390/plants11050610

**Published:** 2022-02-24

**Authors:** Amir Mahgoub Awadelkareem, Eyad Al-Shammari, AbdElmoneim O. Elkhalifa, Mohd Adnan, Arif Jamal Siddiqui, Danish Mahmood, Z. R. Azaz Ahmad Azad, Mitesh Patel, Khalid Mehmood, Corina Danciu, Syed Amir Ashraf

**Affiliations:** 1 Department of Clinical Nutrition, College of Applied Medical Sciences, University of Hail, P.O. Box 2440, Hail 34464, Saudi Arabia; mahgoubamir22@gmail.com (A.M.A.); eyadhealth@hotmail.com (E.A.-S.); ao.abdalla@uoh.edu.sa (A.O.E.); 2 Department of Biology, College of Science, University of Hail, P.O. Box 2440, Hail 34464, Saudi Arabia; drmohdadnan@gmail.com (M.A.); arifjamal13@gmail.com (A.J.S.); 3 Department of Pharmacology and Toxicology, Unaizah College of Pharmacy, Qassim University, P.O. Box 6688, Qassim 51452, Saudi Arabia; ma.alam@qu.edu.sa; 4 Department of Post-Harvest Engineering and Technology, Aligarh Muslim University, Aligarh 202002, India; zrazad@gmail.com; 5 Bapalal Vaidya Botanical Research Centre, Department of Biosciences, Veer Narmad South Gujarat University, Surat 395007, India; patelmeet15@gmail.com; 6 Department of Pharmaceutics, College of Pharmacy, University of Hail, P.O. Box 2440, Hail 34464, Saudi Arabia; adckhalid@gmail.com; 7 Department of Pharmacognosy, Faculty of Pharmacy, “Victor Babes” University of Medicine and Pharmacy, 2 Eftimie Murgu Square, 300041 Timisoara, Romania; corina.danciu@umft.ro

**Keywords:** *Eruca sativa* Miller, food-borne pathogens, biofilms, edible plants, adhesion proteins, extracellular polysaccharide, molecular docking, scanning electron microscopy, light microscopy

## Abstract

Bacterial cells have the ability to form biofilm onto the surfaces of food matrixes and on food processing equipment, leading to a source of food contamination posing serious health implications. Therefore, our study aimed to determine the effect of *Eruca sativa* Miller *(E. sativa)* crude extract against biofilms of food-borne bacteria along with in silico approaches to investigate adhesion proteins responsible for biofilm activity against the identified phytochemicals. The antibacterial potential of crude extract was evaluated using agar well diffusion technique and combinations of light and scanning electron microscopy to assess the efficacy of crude extract against the developed biofilms. Our results showed that crude extract of *E. sativa* was active against all tested food-borne bacteria, exhibiting a rapid kinetics of killing bacteria in a time-dependent manner. MIC and MBC values of *E. sativa* crude extract were found to be ranging from 125 to 500 µg/mL and 250 to 1000 µg/mL respectively. Furthermore, inhibition of developed biofilm by *E sativa* was found to be ranging from 58.68% to 73.45% for all the tested strains. The crude extract also reduced the viability of bacterial cells within biofilms and amount of EPS (ranging 59.73–82.77%) in the biofilm matrix. Additionally, the microscopic images also revealed significant disruption in the structure of biofilms. A molecular docking analysis of *E. sativa* phytochemicals showed interaction with active site of adhesion proteins *Sortase A*, *EspA*, *OprD*, and *type IV b pilin* of *S. aureus*, *E. coli*, *P. aeruginosa*, and *S. enterica ser.* typhi, respectively. Thus, our findings represent the first demonstration of *E. sativa* crude extract’s bioactivity and potency against food-borne bacteria in their planktonic forms, as well as against the developed biofilms. Therefore, a possible mechanistic approach for inhibition of biofilm via targeting adhesion proteins can be explored further to target biofilm producing food-borne bacterial pathogens.

## 1. Introduction

*Eruca sativa* Miller *(E. sativa)*, commonly known as ‘Rocket plant’, belongs to the *Brassicaceae* family and it is a popular vegetable salad in many countries. It is an important aromatic herb commonly employed in foods and drugs. It is used in traditional medicine to enhance fertility and sperm production, to enhance the digestive process and kidney activity, and to fight against eye infection [1]. It has been reported to possess antimicrobial, anti-genotoxic, anticancer, analgesic, antioxidant, anti-diabetic, anti-acne, anti-hyperlipidemic, anti-hyperglycemic, anti-hyperuricaemic and anti-inflammatory properties [2,3,4,5,6,7,8]. Owing to its antimicrobial potential, rocket plant could be used in the food manufacturing or processing industries as a food preservative or as a nutraceutical to regulate monitor and control the activity of food-borne pathogens.

Food spoilage by different food-borne pathogens remains a serious problem for the food industry around the globe and poses a serious risk to human health [9]. The resistance of food-borne pathogens to numerous unfavorable environmental conditions (salt, heat, cold, acid) and the ability to develop biofilms on any kind of biotic and abiotic surfaces are major aspects for food-borne pathogens to persist in food industry environments on a variety of surfaces (ceramics, glass, stainless steel, plastic, polypropylene, rubber, and on food products) [10]. The development of bacterial biofilms in the food industry environment provides numerous benefits to bacteria, such as protection towards desiccation (physical resistance), liquid streams in pipelines (mechanical resistance), disinfectants, antimicrobials, and chemicals utilized in the industry (chemical resistance) [11,12].

Moreover, inside biofilms, bacterial cells are different from their planktonic counterparts in growth pattern and gene expression, which specifically regulate the development of biofilms beneath diverse conditions [13,14,15]. Examples of bacteria which majorly cause food contamination and food-borne diseases are *Escherichia coli* (*E. coli*), *Staphylococcus aureus* (*S. aureus*), *Pseudomonas aeruginosa* (*P. aeruginosa*), *Listeria monocytogenes* (*L. monocytogenes*), *Salmonella enterica* (*S. enterica ser.* typhi), *Campylobacter jejuni* (*C. jejuni*), *Bacillus cereus* (*B. cereus*), etc. These food-borne pathogens are capable of forming biofilms on any type of surfaces in food industry and can contaminate food products at any step during processing, distribution, and storage [16]. Therefore, control of biofilms in food industry is a serious issue, which requires the discovery of novel, natural biologically active compounds as an alternative for currently used chemically synthesized antimicrobial agents.

In recent years, the use of antimicrobial agents derived from natural resources has been widely accepted by consumers, as it is considered to be free from any chemical agents [17]. Furthermore, consumers continue to utilize aromatic herbs to give aroma and flavor to food. Thus, numerous studies have been mainly focused on various types of extracts of aromatic plants and their essential oils, which have been reported as having enormous potential as food preservatives along with significant benefits for human health [18].

Even though the antibacterial properties of *E. sativa* have been reported against different pathogenic bacteria in their planktonic form, there are no reports on its antibiofilm activity. Hence, the determination of antibiofilm potential of *E. sativa* crude extract is absolutely an area to investigate. Therefore, this study was aimed to evaluate the effect of *E. sativa* crude extract on the formation of biofilm of food-borne pathogens such as, *E. coli*, *P. aeruginosa*, *S. enterica ser.* typhi, and *S. aureus* via different in vitro assays. Additionally, an in silico approach was also carried out to understand the possible molecular mechanism behind its antibiofilm potential.

## 2. Results

### 2.1. Phytochemical Screening

**Figure 1:**
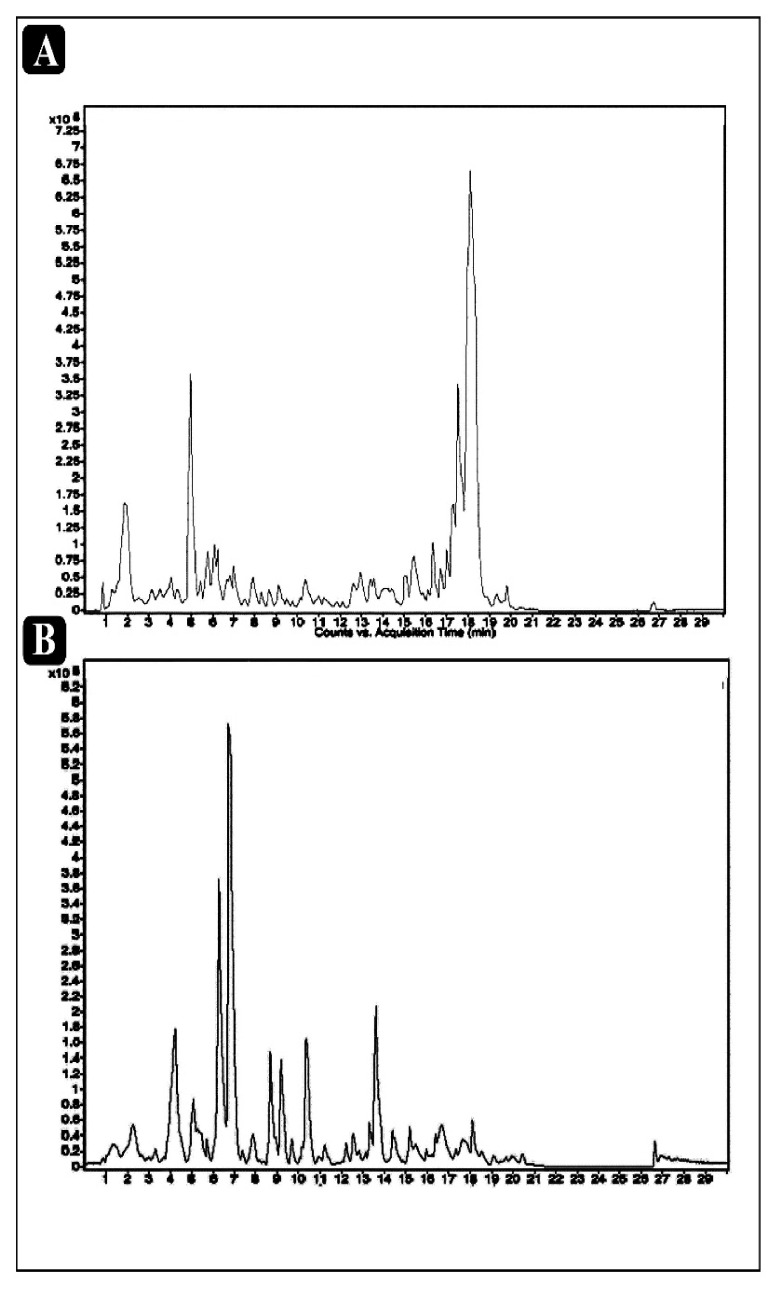
A chromatogram of *E. sativa* crude extract obtained through HR-LC/MS analysis. (**A**) Positive analysis, (**B**) negative analysis.

Phytochemical qualitative analysis of crude extract of *E. sativa* confirmed the occurrence of alkaloids, flavonoids, tannins, phenolics, terpenoids, glycosides, saponins, and carbohydrates in *E. sativa* crude extract (Table 1). Identified phytocompounds with their mass spectra details can be seen in the supplied Supplementary Figure S1 and the high-resolution–liquid chromatography mass spectroscopy (HR-LC/MS), which was used to obtain a chromatogram of *E sativa* crude extract, is presented in Figure 1.

### 2.2. Antibacterial Activity of E. sativa

**Figure 2:**
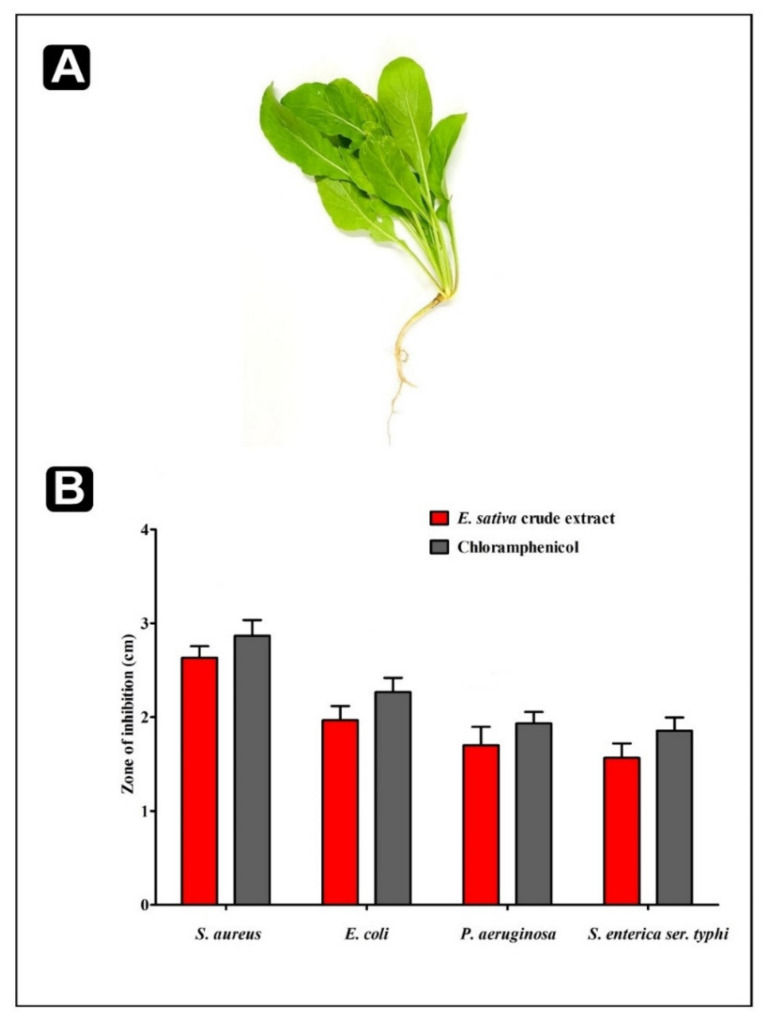
*E. sativa* plant and its antibacterial activity. (**A**) close up of plant; (**B**) antibacterial activity against *S. aureus*, *E. coli*, *P. aeruginosa,* and *S. enterica ser.* typhi. All experiments were carried out in triplicate, and data represent the mean ± SD.

The antibacterial potential of *E. sativa* crude extract was determined by agar well diffusion method against food-borne bacteria, namely *S. aureus, P. aeruginosa, E. coli*, and *S. enterica ser.* typhi. The crude extract was found to be susceptible against all the tested strains. The maximum zone of inhibition was obtained against *S. aureus* (2.63 ± 0.32 cm), whereas the minimum zone of inhibition was obtained against *S. enterica ser.* typhi (1.56 ± 0.15 cm) (Figure 2). Furthermore, the antagonistic ability of *E. sativa* crude extract was assessed via the determination of MIC and MBC values against the food-borne pathogens. MIC and MBC values of *E. sativa* crude extract was found to be ranging from 125 to 500 µg/mL and 250 to 1000 µg/mL respectively as presented in Table 2.

### 2.3. Determination of Growth Pattern of Food-Borne Bacteria

**Figure 3:**
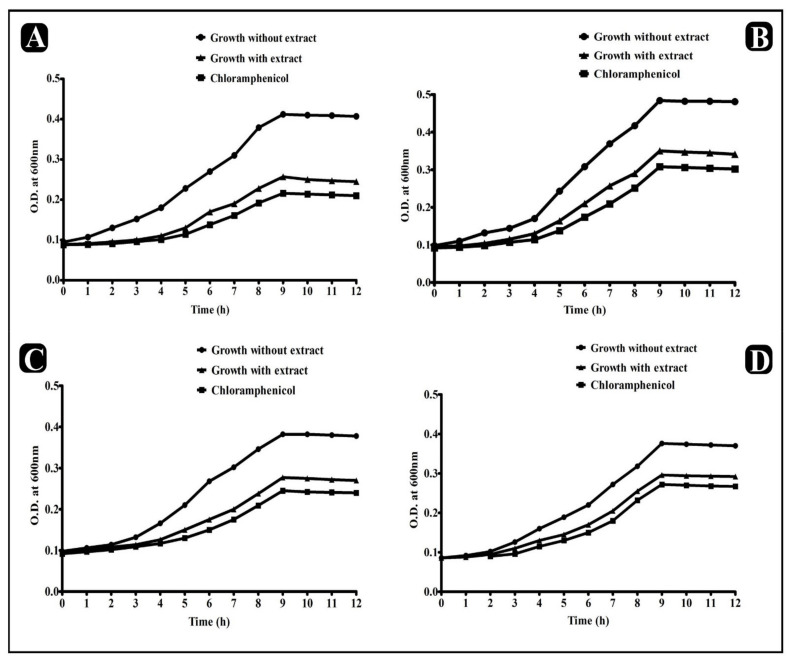
Growth curve analysis of bacteria (with and without *E. sativa* extract). (**A**) Growth curve pattern of *S. aureus*. (**B**) Growth curve pattern of *E. coli*. (**C**) Growth curve pattern of *P. aeruginosa*. (**D**) Growth curve pattern of *S. enterica ser.* typhi.

To determine the inhibitory effect on the growth pattern of bacteria, a growth curve analysis was performed in the presence of crude extract. Our results showed that crude extract had an efficient inhibition in the growth of all tested strains. Moreover, in comparison with control-selected bacterial strains, growth against the *E. sativa* extract was characterized by an extended lag phase and a slow log phase (Figure 3).

### 2.4. Effect of E. sativa Crude Extract on Established Biofilms and on Their Adhesion Properties

**Figure 4:**
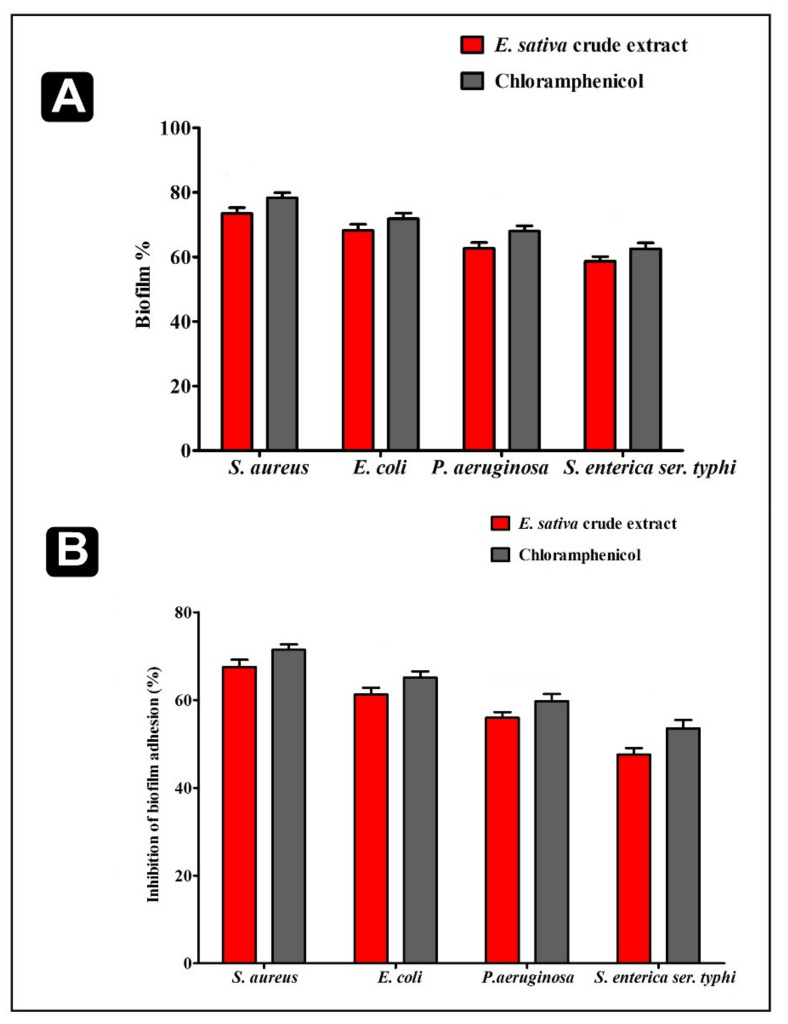
Antibiofilm potential of *E. sativa.* (**A**) Effect of *E. sativa* crude extract on established biofilms of *S. aureus*, *E. coli*, *P. aeruginosa* and *S. enterica ser.* typhi and (**B**) Effect of *E. sativa* crude extract on adherence ability of *S. aureus*, *E. coli*, *P. aeruginosa* and *S. enterica ser.* typhi. All experiments were carried out in triplicate, and data represent the mean ± SD.

The disruption of the developed biofilms and the ability of crude extract to inhibit their adhesion ability were assessed at the MIC levels. We found that the disruption and adhesion of biofilms were considerably efficient at MIC concentration. Furthermore, inhibition of developed biofilms by crude extract of *E. sativa* was about 73.45% for *S. aureus*, 62.70% for *P. aeruginosa*, 68.17% for *E. coli*, and 58.68% for *S. enterica ser.* typhi. The crude extract of *E. sativa* also reduced the biofilms adhesion with percentages of inhibition of 67.50% for *S. aureus*, 61.23% for *E. coli*, 55.86% for *P. aeruginosa*, and 47.59% for *S. enterica ser.* typhi (Figure 4).

### 2.5. Effect of E. sativa Crude Extract on Bacterial Cells Inside Biofilms

**Figure 5:**
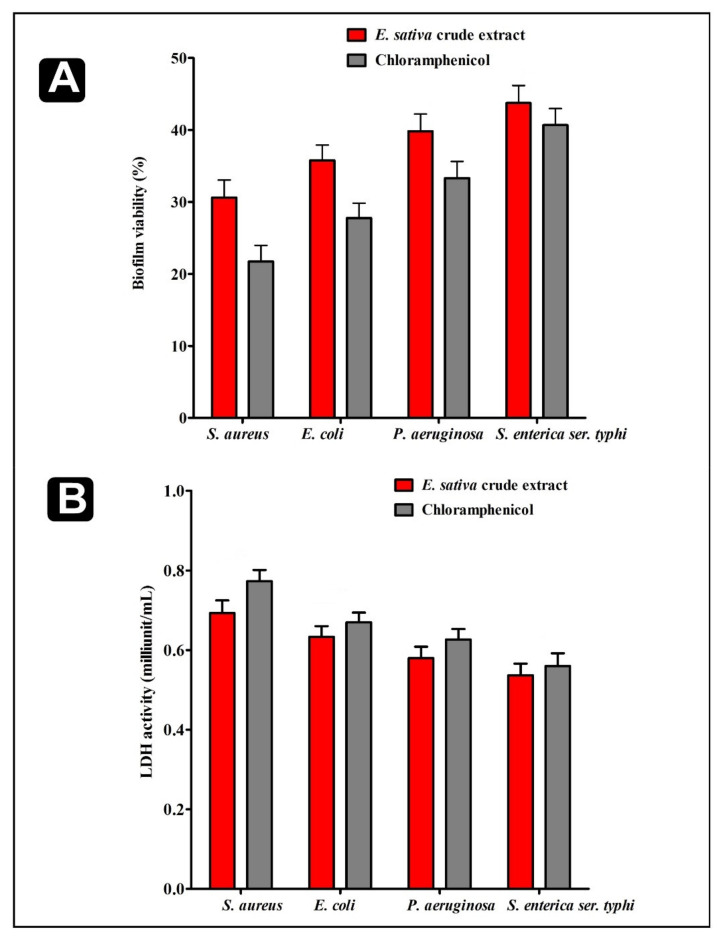
Results of XTT reduction assay and LDH activity assay. (**A**) Percentage of bacterial viability within biofilms measured by the XTT assay at respective MICs. (**B**) Bacterial cell damage within the biofilm based on LDH activity in the presence of *E. sativa* crude extract at their respective MICs. All experiments were carried out in triplicate, and data represent the mean ± SD.

The 2,3-Bis(2-methoxy-4-nitro-5-sulfophenyl)-5-[(phenyl-amino)carbonyl]-2H-tetrazolium hydroxide (XTT) reduction assay and Lactate dehydrogenase(LDH) activity assay was carried out to determine the bacterial cells viability inside the biofilms. The XTT assay revealed that bacterial cells viability inside the biofilms declined efficiently with diverse sensitivities upon the treatment of *E. sativa* crude extract (Figure 5A). Subsequently activities of LDH enzyme in the bacterial supernatant were also investigated. The activity of LDH enzyme was well detected upon the disruption of bacterial cell membrane. The LDH enzyme activity showed that the activity of LDH enzyme in supernatant was increased upon the treatment of *E. sativa* crude extract (Figure 5B). These results suggested that crude extract of *E. sativa* could efficiently inhibit the viability of biofilms of the tested bacterial strains.

### 2.6. Microscopic Analysis of Disruption of Preformed Biofilm

**Figure 6:**
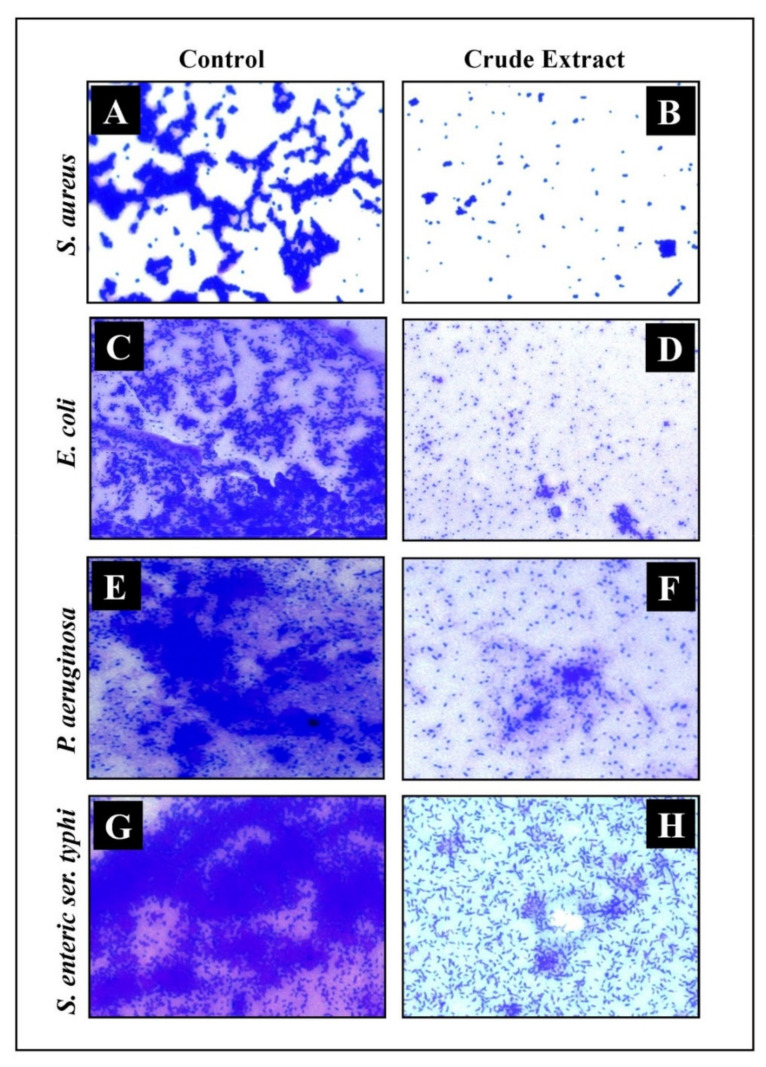
Micrographs of the disrupted matured biofilms of tested strains formed on glass surfaces by the *E. sativa* crude extract at their respective MICs by LM; (**A**,**C**,**E**,**G**) Growth control; (**B**,**D**,**F**,**H**) *E. sativa* crude extract treatment.

**Figure 7:**
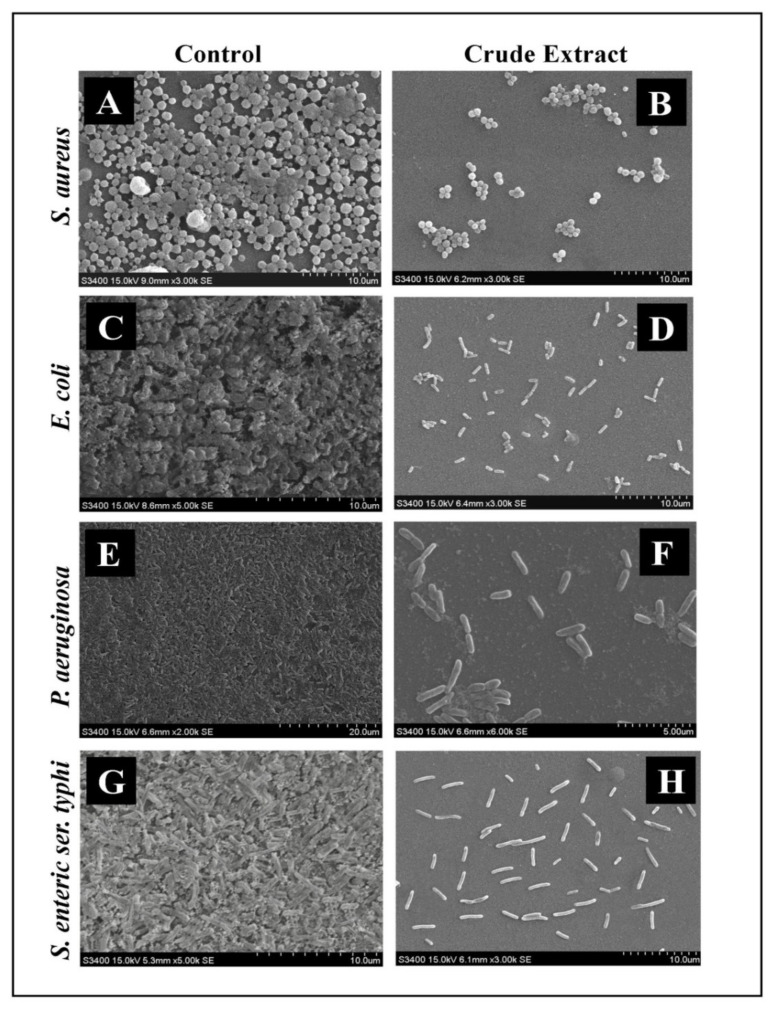
SEM micrographs of the disrupted matured biofilms of tested strains formed on glass surfaces by the *E. sativa* crude extract at their respective MICs; (**A**,**C**,**E**,**G**) Growth control; (**B**,**D**,**F**,**H**) *E. sativa* crude extract treatment.

Light Microscopy (LM) and Scanning Electron Microscopy (SEM) analysis of the developed biofilm and effect of crude extract at its MIC values over glass cover slips were recorded. In LM, a thick, knit-like mat of biofilms stained with crystal violet was observed in control, whereas a reduction in thickness and minimum manifestation of micro-colonies was observed in *E. sativa* crude extract treated biofilms (Figure 6). The ultrastructure of biofilms developed by different food-borne pathogens presented with or without *E. sativa* crude extract was analyzed under SEM. In SEM, typical multilayer biofilms growth was observed in control samples, whereas a significant reduction in biofilm formation along with the disruption of the bacteria’s original structure was observed in the treated samples. Additionally, SEM analysis also showed the bacterial cell wall with distorted and irregular shapes when the sample was treated with *E. sativa* crude extract (Figure 7).

### 2.7. Effect on Exopolysaccharide (EPS) Production

**Figure 8:**
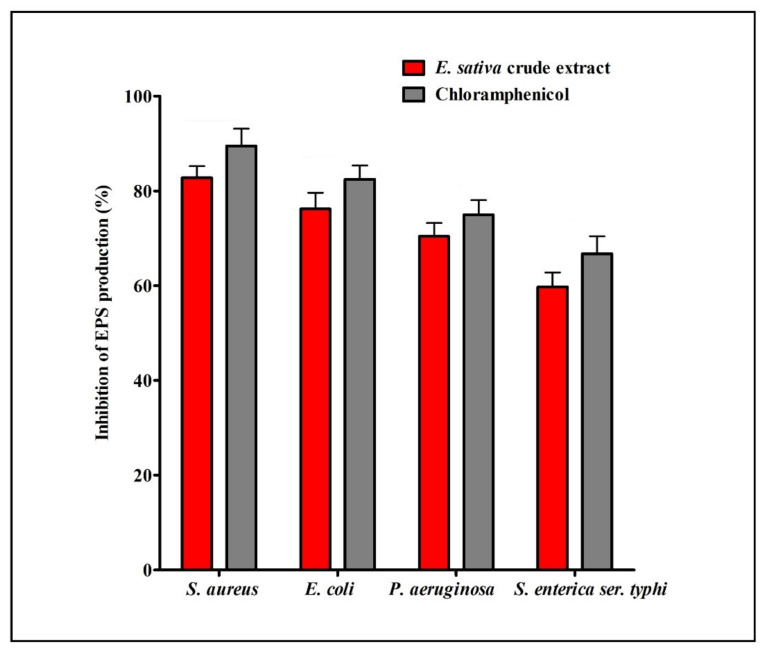
Result of total EPS production inhibition (%) by different bacterial strains in the presence of *E. sativa* crude extract at their respective MICs. All experiments were carried out in triplicate, and data represent the mean ± SD.

EPS is the main component of biofilm, which offers an ideal environment for chemical reactions, entrapment of nutrients, and defense against adverse environmental conditions. Our results showed that total production of EPS at MIC values of *E. sativa* crude extract was significantly reduced in all the tested bacteria. Furthermore, in comparison with control, EPS production was inhibited by 82.77% for *S. aureus*, 76.19% for *E. coli*, 70.42% for *P. aeruginosa*, and 59.73% for *S. enterica ser.* typhi (Figure 8).

### 2.8. Molecular Docking Analysis

**Figure 9:**
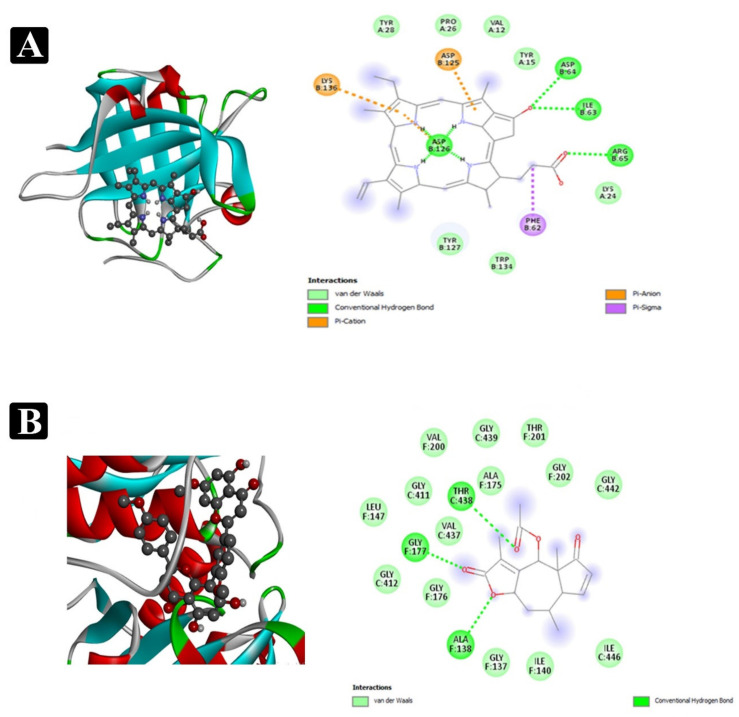
Interactions of phytochemicals which have higher binding affinity with 1T2P adhesion protein of *S. aureus* and 1X0U adhesion protein of *E. coli*. (**A**) Visualization of docking analysis of pyropheophorbide a with 1T2P. (**B**) Visualization of docking analysis of sciadopitysin with 1X0U.

**Figure 10:**
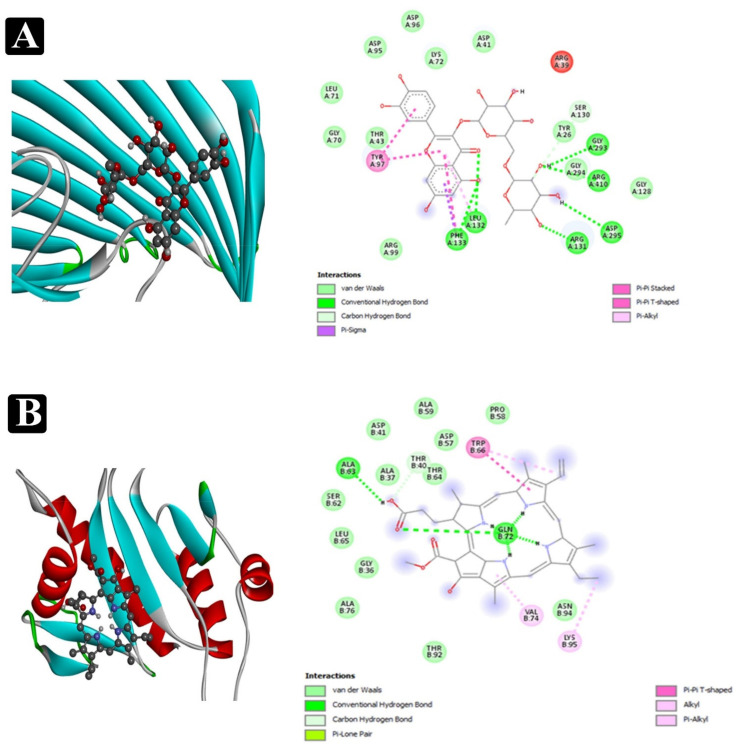
Interactions of phytochemicals which have higher binding affinity with 3SY7 adhesin proteins of *P. aeruginosa* and 3FHU adhesin proteins of *S. enterica ser.* typhi. (**A**) Visualization of docking analysis of rutin with 3SY7. (**B**) Visualization of docking analysis of pheophorbide a with 3FHU.

AutoDock Vina, one of the most renowned tools for molecular docking, was applied to foretell the binding affinity between the phytochemical constituents identified and adhesion proteins of food-borne pathogens. Molecular docking analysis results showed that lower binding energy of the phytochemical constituents against the targeted proteins has the highest binding affinity towards the adhesion proteins. Binding energy of the top-rated pose of ligand-receptor complex are presented in Table 3 and Supplementary Table S2. The active sites covered by compounds in different ways can be observed in Figure 9 and Figure 10.

## 3. Discussion

For the last few years, biofilm has been a serious problem for food industry, as it creates technological and severe hygienic concerns via cross contaminations. In food industry, biofilm of different food-borne pathogens can develop on different food products as well as on any type of contact surfaces or any food processing equipment [19]. Such development of biofilm increases the chance of food-borne pathogens to spoil food, cause contamination, and to survive in adverse environmental conditions such as low temperatures, low pH, salinity, disinfection, and etc., which are frequent conditions in food processing [20]. Furthermore, development of resistant bacterial cells inside the biofilm against the antimicrobial agents occurs due to the presence of extracellular polymeric substances [21].

Hence, traditional sanitizers such as hydrogen peroxide, chlorine, peracetic acid, and quaternary ammonium chloride are used to control the activity of pathogens in the food industries. These antimicrobials are supposed to have an inadequate efficacy against the biofilms [22]. Therefore, the development of resistance incentivizes that the utilization of exceedingly higher concentrations of chemical antimicrobial agents which can be toxic or carcinogenic [23]. Hence, there is an urgent need for novel antimicrobials, which can efficiently control the biofilm growth of different food-borne pathogens in food industry.

In recent years, antimicrobial agents derived from different natural resources gained higher interest. Amongst medicinal plants are the most important as they possess diverse type of phytochemicals, which can significantly target different sites or pathways of the bacterial pathogens hence stop or inhibiting the growth of pathogens [12,24,25,26,27,28,29]. Numerous biologically active molecules from plants proffer a repository of antimicrobial compounds and have drawn substantial research interest. Despite the fact that nature of these compounds is identified as potent antimicrobial agents, the knowledge about the mechanisms of their mode of action is not fully available, nor are the precise individual molecules identified [30]. Although reports on the antimicrobial activities of medicinal plants phytochemicals exist, detailed systematic studies on their antibiofilm potential are scarce. Therefore, the present study, the first of its kind, aimed to evaluate the antibacterial and antibiofilm potential of one of the medicinally important plant *E. sativa* against different food-borne pathogens.

The crude extract of *E. sativa* was found to be effective against all the selected Gram-positive and Gram-negative food pathogenic bacteria. Furthermore, our results showed that the present study is supported well by the previous findings, which reported the antibacterial potential of *E. sativa* [1,2,8,31] by using the agar cup diffusion method. Kauba et al. (2015) reported similar results with *E. sativa* ethanol extract against tested pathogens such as *S. typhimurium* (IZ = 16.7 mm), *B. subtilis* (IZ = 16.6 mm), *E. coli* (IZ = 16.0 mm), and *B. thuringensis* (IZ = 15.6 mm) [32]. Additionally, Khoobchandani et al. (2010) reported the antimicrobial activity of crude extract of different parts of *E. sativa* against two Gram-positive and three Gram-negative bacteria [33]. Among them, the highest activity was reported by seed oil against Gram-positive bacteria compared with Gram-negative bacteria. Additionally, Qaddoumi and El-banna (2019) reported the antagonistic activity of aqueous extract of *E. sativa* towards *E. coli* (IZ = 19.0 mm) and *S. aureus* (IZ = 12.0 mm) [8]. In the same study, antimicrobial activity of crude extract of ethyl acetate had no antimicrobial activity towards the tested pathogens. In another study, Rizwana et al. (2016) reported the antimicrobial activity of ethanol, methanol, and chloroform extract of *E. sativa* against different Gram-positive and Gram-negative bacteria [34]. Among them, higher inhibition activity was found with ethyl acetate and chloroform extract against *S. aureus* (IZ = 25.66 mm, 23.16 mm), respectively followed by methanol and ethanol (IZ = 16 mm, 14.33 mm).

A very well-known curve analysis, i.e., time-kill curve analysis has been more often used to estimate the antimicrobial cumulative effects by monitoring the growth as well as death by measuring the antimicrobial concentrations [35]. The antibacterial drug exerts a time-dependent bactericidal effect when its concentration exceeds the MIC for the respective bacteria, while the concentration-dependent antimicrobial activity is found while the concentration of antibiotics is high at the binding site, leading to death of the bacteria [36]. According to our time-kill kinetics analyses, the crude extract of *E. sativa* presented rapid time-dependent bacterial killing kinetics. All the tested food-borne pathogens had an extended lag phase and a slow log phase when exposed to crude extract of *E. sativa*. The lag phase of the bacterial growth is a response at the adaptation period by first division of the bacterial cell, which might repair macromolecular damage caused by the ambient from which the cell came and synthesize the cellular components required for growth. The synthesis of macromolecules would therefore be a possible target by crude extract of *E. sativa*. The *E. sativa* crude extract was found to be effective in disrupting biofilms and decreasing the adhesion for different tested food-borne pathogens at their respective MICs value. This was additionally validated via LM and SEM. The universal crystal violet assay in LM revealed that crude extract of *E. sativa* was capable of removing biofilms of the selected food-borne pathogens. Furthermore, SEM analysis was evident in the reduction of multilayer biofilms growth as well as planktonic cells by affecting the integrity of cell wall. One more important thing observed during SEM analysis is that in the presence of *E. sativa* crude extract, bacterial strains were not able to keep their usual morphological structure due to the disruption of the cell wall, which causes disruption in the appearance of bacterial cell clusters. Subsequently, the formation of biofilm starts with the initial attachment (adhesion) of the bacterial cell to the surface and then with the help of EPS, a typical shape of biofilm forms [10]. Moreover, quorum sensing (QS) is one more process that is considered as an important one for the formation of biofilms in which bacterial cells can communicate to one another via mode of different signaling molecular pathways, and it has been widely studied for the control of biofilms [37]. However, earlier reports suggest that inhibition of biofilm activity of plants phytochemical constituents via targeting QS mechanism depends on the density and needs the suitable population of bacterial cells [24]. More importantly, results of our study showed that the formation of biofilm by food-borne pathogens could be stopped at the initial step by preventing the adhesion, which might be useful in developing a novel therapeutic approach.

Few studies have documented phytochemicals’ role in preventing biofilms by inhibiting adhesion through different mechanisms. Six bacterial strains have been reported to be inhibited from establishing biofilms when extracts of plants interacted with additional forces like Brownian, Lifshitz-Van der Waals, sedimentation, and electrostatic interactions, which promote bacterial attachment to surfaces [38]. In addition, to prevent the biofilm attachment, plant extracts can also hinder the availability of inorganic as well as organic nutrients, which are considered to be crucial for bacterial adhesion as well as bacterial growth [39]. According to earlier reports, crude extract of *Psidium guajava*, both in ethanol and in acetone, blocked *Streptococcus mutans* from adhering to surfaces [40]. Similarly, the anti-adhesion potential of crude extract of several medicinal plants such as *E. brasiliensis*, *E. myrcianthes*, *E. leitonii* and *E. involucrate* has been also reported against *C. albicans* [32].

In conjunction with our findings on the outcome of *E. sativa* crude extract on biofilm matrix composition, these findings suggested that crude extract of *E. sativa* also inhibits EPS synthesis. Overall, our data suggested that crude extract of *E. sativa* limits the growth of biofilm. The EPS matrix is considered as the most distinguishing attributes of a biofilm that make a gel like three-dimensional structure in which bacteria are mostly immobilized [41]. The *E. sativa* crude extract was also found to influence the bacterial cells’ viability within microbial biofilms. The XTT reduction assay confirmed that biofilm of tested food pathogens was reduced after the treatment of *E. sativa* crude extract. Aside from this, crude extract of *E. sativa* could also affect the cell composition of bacteria within the biofilm. Thoroughly, in vitro analysis of the current study exhibited that crude extract of *E. sativa* controls the development of biofilms of food-borne pathogens.

Furthermore, a molecular docking analysis of identified phytochemical constituents from the crude extract of *E. sativa* was performed to find out a possible mode of action associated with the recorded antibiofilm activity of crude extract in detail. This process was analyzed to the binding affinity of phytochemical constituents to target adhesion proteins. The adhesion of bacterial cell to a food matrix surface is the first and most important step in the process of biofilm development of food-borne pathogens. The well-known adhesion proteins from each tested food-borne bacteria such as *Sortase A (S. aureus), EspA (E. coli), OprD (P. aeruginosa)* and *type IV b pilin (S. enterica ser.* typhi) were used. Subsequently, inhibition of these adhesion proteins led to the inhibition process of biofilm development and finally their virulence factors.

A number of the identified compounds from the crude extract of *E. sativa* via HR-LCMS were reported in our previously conducted study (Unpublished data) to retain broad ranges of antimicrobial properties [42,43,44]. Most of the identified compounds in this study via HR-LC/MS are in accordance with the previously reported chromatographic analysis of *E sativa* crude extract [45,46,47,48,49,50,51]. They might also contribute to the inhibition of bacterial growth and antibiofilm activity in the present studies. Hence, in light of the results obtained in the in vitro study, it was considered worthwhile to perform molecular docking studies that correlate both in vitro and in silico results. In the drug discovery process, docking studies can be used at various stages, for example, to predict a ligand-receptor interaction or to rank compounds by their binding energies [52]. Furthermore, among the identified phytoconstituents, pyropheophorbide-a had highest binding affinity (−9.4) with 1T2P and exhibited the suitable binding approach at the active site of *Sortase A* (Lys136, Asp125, Phe62, Asp126, Asp64, Ile63, and Arg65), Sciadopitysin had highest binding affinity (−10.8) with 1XOU and exhibited a suitable binding approach at the active site of *EspA* (Arg512, Lys516, Thr398, Glu396, Tyr392, Val158, Lys515, Tyr513, and Arg512). Rutin had the highest binding affinity (−9.8) with 3SY7 and exhibited a suitable binding mode at the active site of *OprD* (Tyr97, Phe133, Leu132, Arg39, Tyr26, Gly293, Arg410, Asp295, and Arg131). Pyropheophorbide-a again, the highest binding affinity (−8.8) with 3FHU and exhibited an appropriate binding approach at the active site of *type IV b pilin* (Ala63, Thr40, Trp66, Gln72, Val74, and Lys95). Appropriate intermolecular hydrogen bonding interactions between all the detected phytoconstituents present in *E sativa* extract and the active site of *Sortase A, EspA, OprD*, and 3FHU proteins were observed [53,54]. The residue Glu89, Glu17, Asp126, Asn38, Asp126, Asp64, Asp125, Lys146, and Asp125 from *Sortase A* of *S. aureus*, Lys515, Glu396, Tyr513, Lys516, Pro282, Pro334, Gly136, and Ala175 from *EspA* of *E. coli*, Leu132, Asn218, Gln119, Arg39, Gly293, Arg410, and Asp295 from *OprD* of *P. aeruginosa*, and Ala63, Gln72, Thr149, Gly84, Asp78, Thr92, and Asn94 from *type IV b pilin* of *S. enterica ser.* typhi formed strong interactions with the antibiofilm agents with standard hydrogen binding patterns. Thus, interaction of these key residues with antibiofilm agents might interfere with the crucial step of biofilm formation, namely adhesion.

## 4. Materials and Methods

### 4.1. Sample Collection, Identification and Extraction Process

In this study, plants of *E. sativa* were grown and collected after maturity. The voucher specimen (BVBRC146) was deposited in the herbarium of Bapalal Vaidya Botanical Research Centre, Veer Narmad South Gujarat University, Surat, Gujarat, India. The collected plant material was washed and dried in an oven. The dried whole plants were ground into a fine powder with the help of an electric grinder and transfer into an airtight container for further storage. Later, preparation of crude extract using *E. sativa* plants, powder (25 g) was achieved in 85% ethanol in overnight conditions with vigorous shaking at 120 rpm at 37 °C. Subsequently, overnight soaked powders were filtered using Whatman no. 1 filter paper and the ethanolic phase collected, and sample extract were concentrated using a rotary evaporator. Concentrated residues were dissolved in 10% DMSO (dimethyl sulfoxide) and volume adjusted to 1 mg/mL of plant extract. The crude extract was properly mixed via sonication and sterilized using a syringe filter of 0.25 µm filter pore size before use.

### 4.2. Qualitative Phytochemical Analysis

The qualitative phytochemical study of prepared *E. sativa* extract was carried out by means of standard methods as reported by Arunachalam et al. (2015) [55]. The results were represented qualitatively as positive (+) or negative (−).

### 4.3. Antibacterial Assay

#### 4.3.1. Bacterial Strains

The antibacterial activity of *E. sativa* crude extract was carried out against some of the food-borne pathogens such as, *E. coli* (MTCC 9537), *S. enterica ser.* typhi (MTCC 8767), *P. aeruginosa* (MTCC 741), and *S. aureus* (MTCC 96). All the selected strains of pathogenic bacteria were acquired from the Microbial Type Culture Collection (MTCC), India and maintained on Muller-Hinton Agar (MHA). Fresh bacterial inoculum was grown by inoculating a single pure colony of respective bacterial strain and the turbidity of the bacterial cell culture were made with 0.5 Mc Farland standards (10^8^ colony forming units/mL) by adjusting by means of saline solution.

#### 4.3.2. Agar Well Diffusion Method

The antibacterial activities of *E. sativa* ethanolic extract were determined by using the agar well-diffusion technique on MHA. The active cultures of each bacterial strain (100 µL) were evenly spread onto the MHA plates and wells were created at the center of the plates with the help of a sterile gel puncture. 60 µL of *E. sativa* crude extract (1 mg/mL) was inoculated into each well and incubated at 37 °C for 24 h. The ethanolic extract having antimicrobial activity showed the inhibition of microbial growth and the zone of inhibition was measured. The standard antibiotic solution of chloramphenicol (1 mg/mL) was used as a positive control; however, DMSO (10%) was used as a negative control [56,57].

#### 4.3.3. Estimation of Minimum Inhibitory Concentration (MIC) Values

The MIC value of *E. sativa* crude extract was estimated by using 96-well microtiter plates as per the microdilution method mentioned by Clinical and Laboratory Standards Institute (CLSI, 2014) [58]. The active culture of each bacterial strain was grown freshly from overnight grown culture in MHB plates and the suspended active bacterial cell turbidity were maintained or adjusted at 0.5 Mc Farland standards (10^8^ CFU/mL). The crude extract of *E. sativa* was two-fold diluted ranging from 1000 to 0.48 µg/mL (final volume 80 µL) using DMSO at a concentration <1%. Then, active culture of each bacterial strain (80 µL) was transferred to respective microplate’s wells and incubation was performed at 37 °C for 24 h. Later, absorbance of wells was measured using spectrophotometer at 620 nm. The antibiotic standard used was chloramphenicol as a positive control, and MHB was used as a sterility control. Consequently, the lowest concentrations (MIC) of *E. sativa* crude extract were measured by evaluating the bacterial growth inhibition [15].

#### 4.3.4. Estimation of Minimum Bactericidal Concentration (MBC)

The assessment of MBC values was performed followed by the MIC estimation. 10 µL samples were pipetted from the 96-well plates, where there was no apparent growth of bacterial cells and furthermore pipetted samples were spread onto MHA plates. The plates were incubated at 37 °C for 24 h. The value of MBC was measured, at which there was a minimum growth/colony of bacteria [15].

#### 4.3.5. Effect of *E. sativa* Crude Extract on Growth Kinetics of Bacteria

The effect of crude extract on growth kinetics of the bacteria was investigated in the presence and absence of crude extract in the growth medium. The active fresh cultures of the selected bacterial strain (500 µL) were inoculated into 150 mL of nutrient broth containing 1 mL of crude extract (1 mg/mL). A flask without the sample was kept as the control. The absorbance was read at 600 nm at the interval of each 1 h time to check the effect of crude extract on the growth kinetics of bacteria.

### 4.4. Antibiofilm Assays

#### 4.4.1. Effect of *E. sativa* Crude Extract on Established Biofilms

The effect of *E. sativa* crude extract on the developed biofilms was estimated using Lemos et al.’s (2018) method with certain modifications [59]. To develop biofilms, active culture of each bacterial strain (10^7^ cells/mL) was inoculated in 96-well microtiter plates consisting of MHB and 1% glucose. The plates were incubated at 37 °C for 24 h. At the end of incubation, planktonic cells were removed and the wells were gently washed 3 times with normal saline solution. The crude extract of *E. sativa* (200 µL) (MIC) was added to respective wells and bacterial cell were incubated at 37 °C for 24 h. The absorbance was read at 492 nm at 0 h and after 24 h. The medium (MHB) with each bacterial strain was used as biofilm growth control. The percentage inhibition in the biofilm was calculated via the following formula:

[(OD (control) − OD (test)/OD (control)] × 100

#### 4.4.2. Effect of *E. sativa* Crude Extract on the Adherence of Biofilms

Adherence of biofilms and effect of *E. sativa* crude extract on these biofilms were evaluated by using the method described by Plyuta et al. (2013) [60]. MIC levels of crude extract along with fresh active bacterial strains (100 µL) (108 CFU/mL) were incubated in 96-well microtiter plates at 37 °C for 24 h. After the incubation, planktonic cells were discarded and all the selected wells gently washed with phosphate buffered saline (PBS) (200 µL). Subsequently, developed biofilms along with adherent cells were stained with 0.1% crystal violet (200 µL) and additional incubation was performed at 37 °C for 30 min. Thereafter, excessive stain was gently washed off with the help of PBS solution and microtiter plates were fixed with 95% ethanol, followed by further incubation for 15 min at 37 °C. The absorbance of the treated sample was read at 590 nm using a spectrophotometer. The percentage inhibition was calculated via the following formula:

[(OD (control) − OD (test)/OD (control)] × 100

### 4.5. Microscopic Analysis

#### 4.5.1. Determination of Antibiofilm Activity Using Light Microscopy

The antibiofilm effect of *E. sativa* crude extract against food-borne pathogens was evaluated using light microscopic analysis as per the method described by Musthafa et al. (2010) [61]. In brief, the fresh and active culture of selected bacterial strains was inoculated with freshly prepared 5 mL of MHB media containing 1% glucose. After the inoculation, 1 ml of the inoculated broth having a concentration of 10^8^ CFU/mL was transferred to 6-well microtiter plates consisting of a 1 × 1 cm size of cover slips. Thereafter, 500 µL of crude extract of *E. sativa* (final concentration = MIC) was added. Later, the entire plate was incubated at 37 °C for 24 h under static conditions. After incubation, glass cover slips were removed, gently washed with PBS, and stained with 0.1% crystal violet. Thereafter, excessive stain was washed with the help of deionized water and allowed to dry for 5 min. The stained cover slips were observed under a light microscope (Axioscope A1, ZEISS, Oberkochen, Germany).

#### 4.5.2. Determination of Antibiofilm Activity via Scanning Electron Microscopy

The biofilms of each bacterial strain were developed on 1 × 1 cm size of cover slips with all treatments as described above and all the slides were visualized under SEM. The developed biofilms were fixed on glass cover slips using 2.5% of glutaraldehyde at 37 °C for 30 min. Afterwards, cover slips were rinsed and washed with PBS solution 3 times and dehydrated from a graded series of 30%, 50%, 70%, 90%, and 100% of ethanol for 15 min in each. At the end, ethanol was replaced with isoamyl acetate and the samples were freeze-dried. Coverslips were kept on the aluminum holder, covered with gold via E-1010 ion sputter (Hitachi^®^, Tokyo, Japan), and observed under SEM (S-34002N SEM, Hitachi^®^, Tokyo, Japan).

#### 4.5.3. Determination of Biofilm Metabolic Activity

The viability of bacterial cells within the biofilms was determined via the colorimetric XTT reduction test (2,3-Bis(2-methoxy-4-nitro-5-sulfophenyl)-5-[(phenyl-amino)carbonyl]-2H-tetrazolium hydroxide) via the previously reported procedures [62,63]. The active culture of each bacterial strain was inoculated into MHB (200 µL) at an initial turbidity of 0.1 at 600 nm, and grown in the presence and absence of crude extract of *E. sativa* for 24 h at 37 °C. At the end of incubation, plates were washed with sterile distilled water thrice to take out the planktonic cells. Then, 100 µL of PBS and freshly prepared solution of XTT-menadione were added to each well and incubated for 5 h at 37 °C in the dark. After incubation, 100 µL of colored supernatant from each well was transferred in a new 96-well microtiter plate. The absorbance was taken at 480 nm using a microplate reader and the percentage of surviving bacterial population was measured as follows:

[(OD (crude extract treated sample) − OD (negative control)/OD of untreated control)] × 100(1)

#### 4.5.4. Determination of Cell Damage within Biofilms

The lactate dehydrogenase (LDH) assay was carried out to determine the bacterial cell damage inside the biofilms. The active culture of each bacterial strain (100 µL) with MHB (100 µL) was added into 96-well microtiter plates and the plates were incubated for 24 h at 37 °C. At the end of incubation, planktonic cells were removed by washing with PBS thrice. The crude extract of *E. sativa* (MIC) (100 µL) was then added and further incubated for 24 h at 37 °C. Then, supernatant was collected and utilized for the determination of LDH activity by LDH assay kit (Sigma-Aldrich^®^, Bangalore, India). The absorbance was read at 480 nm using a microplate reader. As a negative control, MHB and bacterial culture was used.

#### 4.5.5. Determination of Extracellular Polysaccharide (EPS) Production

The effect of *E. sativa* crude extract on the production of EPS matrix of all the bacterial strain biofilm was determined via the method described by Borucki et al. (2003) [64]. The crude extract of *E. sativa* (MIC) and active culture of each bacterial strain (100 µL) (10^8^ CFU/mL) was incubated for 24 h at 37 °C. At the end of incubation, planktonic cells were removed and the wells were gently washed with PBS (200 µL). The developed biofilms via adherent cells were then stained with 0.01% ruthenium red (Sigma-Aldrich^®^, Bangalore, India) (200 µL). The solution of ruthenium red (200 µL) was added into the wells without biofilms and used as blank. Then, the reaction was followed via further incubation of 1 h at 37 °C. After, the reaction mixture was transferred into a new microtiter plate and the absorbance was taken at 450 nm. The amount of dye associated with the biofilm was calculated via taking the absorbance for the blanks, controls and treated wells.

### 4.6. Molecular Docking Analysis of Phytochemicals of E. sativa with Adhesion Proteins of Food-Borne Pathogens

Molecular docking of the identified phytochemical components of the *E. sativa* via HR-LCMS analysis was carried out against the adhesion proteins of tested food-borne bacterial strains. The crystal structures of the adhesion proteins such as, *Sortase A* of *S. aureus* (PDB: 1T2P), *EspA* of *E. coli* (PDB: 1XOU), *OprD* of *P. aeruginosa* (PDB: 3SY7), and *type IV b pilin* of *S. enterica ser.* typhi (PDB: 3FHU) were downloaded from the Protein Data Bank (RCSBPDB) [53,65,66,67,68]. The three-dimensional molecular structure of all 40 identified compounds was downloaded by using PubChem database and all the molecular structures were converted into PDB format via Open Babel Software [69]. All these compounds were then individually docked against the receptors using AutoDock Vina. By removing the co-crystallized ligand, selected water molecules, and co-factors from the protein to be prepared, the associated residues with the protein were left in the file being prepared by using auto preparation of target protein via MGL Tools 1.5.7. The graphical user interface software was applied to set the grid box for docking simulations. The grid size was set to 126 × 126 × 126 xyz points for 3SY7, the grid size was set to 126 × 114 × 118 xyz points for 1T2P, 130 × 130 × 130 for 1X0U, and 60 × 70 × 60 xyz points for 3FHU with grid point spacing of 0.375 Å. The grid center for 1T2P was designated at dimensions (x, y, and z) −30.329, −19.713, −0.455; for 3SY7 at dimensions (x, y, and z): 24.439, −13.409, 13.726; for 1X0U at dimensions (x, y, and z): 19.293, −4.237, and 86.963; for 3FHU at dimensions (x, y, and z): 55.812, 32.879, 11.895. The grid box was cantered so as to completely enclose the binding sites of both receptors and provide enough room for ligand translation and rotation. As many as nine conformers could be considered for each ligand during the docking process. For analysis of the interactions between ligands and receptors by Discovery Studio visualizer, the conformations with the least free-binding energy were selected.

## 5. Conclusions

Overall, the current study revealed that crude extract of *E. sativa* comprises of a wide range of phytochemicals that possess a significant antibacterial and antibiofilm potential against Gram-positive and Gram-negative food-borne pathogens. Such potency of *E. sativa* crude extract could be due to its ability to target various physiological components, including the macromolecules production as well as membrane destabilization. *E. sativa* crude extract was also found to inhibit bacteria’s ability to form biofilms by hampering adhesion and EPS production. Furthermore, in silico docking analysis was very much useful in identifying novel compounds against pathogenic food-borne bacterial biofilms, which pose a serious risk to the food industry and ultimately to human health.

## Figures and Tables

**Table 1:** Qualitative phytochemical screening of ethanol crude extract of *E. sativa*.

Phytochemicals	Crude Extract
Alkaloids	Positive
Tannins	Positive
Flavonoids	Positive
Phenolics	Positive
Terpenoids	Positive
Glycosides	Positive
Saponins	Positive
Carbohydrates	Positive

**Table 2:** Antibacterial activity of *E. sativa* crude extract.

Bacterial Strain	*E. sativa* Crude Extract (µg/mL)		Chloramphenicol (µg/mL)	
	MIC	MBC	MIC	MBC
*S. aureus*	125	250	7.812	15.625
*E. coli*	250	500	15.65	31.25
*P. aeruginosa*	250	500	62.5	125
*S. enterica ser.* typhi	500	1000	125	250

**Table 3:** Identified phytochemical constituents from crude extract of *E. sativa* via HR-LCMS and their ligand-receptor binding affinities.

Compound Number	Name	1T2P	1X0U	3SY7	3FHU
1	(+/-)-3-[(2-methyl-3-furyl)thio]-2-butanone	−4.3	−5.1	−4.9	−4.3
2	(10Z,14E,16E)-10,14,16-Octadecatrien-12-ynoic acid	−4.9	−5.8	−6.1	−5.5
3	(6beta,8betaOH)-6,8-Dihydroxy-7(11)-eremophilen-12,8-olide	−7.2	−7.6	−7.7	−6.4
4	(S)-2-(Hydroxymethyl)glutarate	−4.6	−5.3	−4.7	−4.3
5	(±)-Rollipyrrole	−6.2	−6.3	−7.3	−5.5
6	1,4-Dimethoxyglucobrassicin	−6.6	−8.0	−7.4	−6.8
7	1-Methoxy-1H-indole-3-carboxaldehyde	−5.2	−6.4	−5.8	−5.1
8	16-Hydroxy hexadecanoic acid	−4.6	−4.8	−5.2	−3.6
9	2-Deoxy-scyllo-inosose	−5.1	−5.5	−5.2	−4.8
10	3,4’,5,6,8-Pentamethoxyflavone	−7.0	−8.1	−7.0	−6.2
11	4-(3-Hydroxy-7-phenyl-6-heptenyl)-1,2-benzenediol	−7.7	−8.2	−7.1	−5.1
12	4-Amino-2-methyl-1-naphthol	−6.5	−7.2	−6.2	−5.6
13	9Z-Octadecenedioic acid	−5.7	−5.7	−5.9	−4.6
14	Afzelechin	−7.5	−8.5	−7.4	−6.3
15	Artomunoxanthentrione epoxide	−8.3	−10.3	−9.1	−7.4
16	Corchorifatty acid F	−6.0	−5.8	−6.4	−5.2
17	Evoxine	−6.0	−7.8	−6.7	−5.6
18	Fraxidin	−6.5	−6.8	−6.4	−5.8
19	Glucoraphanin	−5.9	−6.5	−6.8	−5.9
20	Indoleacrylic acid	−6.2	−7.1	−6.3	−6.3
21	Lactucin	−6.9	−8.0	−7.5	−6.9
22	Linifolin A	−7.7	−8.0	−7.2	−6.2
23	Methyl N-methylanthranilate	−5.3	−5.9	−5.2	−4.9
24	N-(6-Oxo-6H-dibenzo[b,d]pyran-3-yl)maleamic acid	−7.4	−9.1	−8.6	−6.9
25	N-trans-Feruloyl-4-O-methyldopamine	−7.4	−7.9	−7.1	−6.6
26	N6-cis-p-Coumaroylserotonin	−7.8	−8.5	−7.7	−6.8
27	Nopaline	−5.5	−6.6	−6.0	−5.2
28	Oleamide	−5.1	−5.2	−5.4	−4.0
29	Palmitic amide	−4.6	−4.9	−4.9	−4.0
30	Petasitenine	−7.4	−9.0	−8.4	−7.1
31	Pheophorbide a	−8.1	−9.1	−8.5	−8.8
32	Pubesenolide	−6.0	−7.6	−7.7	−6.1
33	Pyrafoline D	−8.8	−9.4	−8.7	−7.7
34	Pyropheophorbide a	−9.4	−10.0	−8.7	−8.0
35	Rutin	−8.5	−9.8	−9.8	−6.8
36	Sciadopitysin	−9.0	−10.8	−9.1	−8.4
37	Serinyl-Hydroxyprolinen	−5.7	−7.0	−5.9	−5.5
38	Terminaline	−7.4	−8.0	−7.9	−6.7
39	Thalidasine	−8.1	−9.1	−8.8	−7.0
40	Trilobolide	−7.2	−8.4	−7.7	−6.6

## Data Availability

All data generated or analyzed during this study are included in this article.

## Supplementary Materials

The following supporting information can be downloaded at: https://www.mdpi.com/article/10.3390/plants11050610/s1, Figure S1: Identified phytocompounds with their mass spectra details; Table S2: Interacting active site residues of receptors with different antibiofilm phytochemical constituents.
