# Supplementary material for: Anti-Adhesion and Antibiofilm Activity of Eruca sativa Miller Extract Targeting Cell Adhesion Proteins of Food-Borne Bacteria as a Potential Mechanism: Combined In Vitro-In Silico Approach
Source: Plants (Basel). 2022 Feb 24;11(5):610. doi: 10.3390/plants11050610 (PMC8912376; doi:10.3390/plants11050610)
Supplement: Supplementary file 1 [file plants-11-00610-s001.zip › S2.pdf]

Table (S2). Interacting active site residues of receptors with different antibiofilm phytochemical constituents.

Receptor–ligand	Receptor ligand interactions	Distance in angstroms	Receptor–ligand	Receptor ligand interactions	Distance in angstroms
**1T2P-Artomunoxanthentrione epoxide**	(ASN38) HD21-O (Ligand)	2.58	**1X0U-Rutin**	(ILE205) Pi-Alkyl interaction	4.25
	(ARG65) HH21-O (Ligand)	2.86		(ILE205) Pi-Alkyl interaction	5.14
	(ARG65) HH11-O (Ligand)	2.05		(AGLU441) OE1-Pi interaction	4.25
	(ARG65) HH11-O (Ligand)	2.43		(THR438) HG1-O (Ligand)	2.30
	(TYR15) HH-O (Ligand)	2.43		(GLY136) O-H (Ligand)	2.59
	(ASP125) OD2-C (Ligand)	3.58		(ALA138) HN-H (Ligand)	2.69
	(ASP126) HN-O (Ligand)	2.57		(ALA175) O-H (Ligand)	3.00
	(ASP126) OD2-C (Ligand)	3.58		(ALA175) O-H (Ligand)	2.52
**1T2P-Pheophorbide a**	(ASN67) HD22 –O (Ligand)	2.45		(GLY177) HN-O (Ligand)	2.11
	(GLU17) OE1-H (Ligand)	2.46		(GLY371) CA -O (Ligand)	2.85
	(LYS24) HZ1-O (Ligand)	2.20		(TYR372) HN-O (Ligand)	2.38
	(THR90) HN-O (Ligand)	2.07		(PRO370) O-O (Ligand)	2.85
	(THR90) HN-O (Ligand)	2.37		(GLY439) HN-O (Ligand)	2.74
	(LYS92) HZ1-O (Ligand)	2.20		(GLU204) OE2-O (Ligand)	2.72
	(LYS92) NZ -pi Cation interaction	4.14	**1X0U-Sciadopitysin**	(ARG512) Pi-Alkyl interaction	4.85
	(GLU89) OE1-H (Ligand)	2.97		(ARG512) Pi-Alkyl interaction	3.89

	(ASP22) OD1 - pi anion interaction	3.20		(ARG512) Pi-Alkyl interaction	5.42
	(ASP22) OD1 - pi Anion interaction	3.48		(LYS516 Pi-Alkyl interaction	4.69
**1T2P-Pyrafoline D**	(TYR127) phenyl ring-CC (Ligand) (Pi- interaction	4.11		(LYS516) CE-O (Ligand)	3.38
	(PRO34) Alkyl-CC (Ligand)	4.40		(LYS516) O-C (Ligand)	3.77
	(ASN38) HD21-OC (Ligand)	2.29		(THR398) HG1-O (Ligand)	2.76
	(TRP134) pi-pi interaction	5.63		(GLU396) O-C (Ligand)	3.49
	(TRP134) pi-pi interaction	5.87		(GLU396) OE2-C (Ligand)	3.68
	(PHE62) pi-pi interaction	4.66		(TYR392) Pi-Alkyl -C (Ligand)	4.98
	(PHE62) pi-pi interaction	4.36		(VAL158) Alkyl-C (Ligand)	5.34
	(TYR15) Pi-Alkyl interaction	5.47		(LYS515) Pi-Alkyl interaction	4.34
	(TYR15) phenyl ring-CC (Ligand) Pi- interaction	5.11		(LYS515) Pi-Alkyl interaction	4.66
	(LYS24) - C (Ligand) Pi alkyl interaction	3.85		(TYR513) O-H (Ligand)	2.95
**1T2P-Pyropheophorbidea**	(LYS136) NZ-Pi interaction	4.94	**1X0U-Terminaline**	(ILE446) Alkyl-Alkyl interaction (Ligand)	4.82
	(ASP125) OD2-Pi interaction	3.76		(GLY411) CA-O (Ligand)	3.30
	(ASP126) OD2-HN (Ligand)	2.33		(GLY412) HN-O (Ligand)	2.43

	(ASP126) OD2-HN (Ligand)	2.33		(GLY412) HN-O (Ligand)	2.11
	(ASP126) OD2-HN (Ligand)	2.18		(GLU204) OE1-C (Ligand)	3.56
	(ASP126) OD2-H (Ligand)	2.56		(MET159) Alkyl-C (Ligand)	4.17
	(ASP126) OD1-Pi interaction	3.72		(TYR513) Pi-Alkyl-C (Ligand)	4.97
	(ASP64) HN-O (Ligand)	2.61		(LYS420) O-C (Ligand)	3.78
	(ILE63) HN-O (Ligand)	2.34	**1X0U-Thalidasine**	(GLU120) OE1-Pi interaction	4.67
	(ARG65) HH12-O (Ligand)	2.42		(GLU120) OE1-C (Ligand)	3.68
	(PHE62) phenyl ring-CC (Ligand) Pi-Sigma interaction	3.89		(GLU120) OE1- Pi interaction	4.12
	(ARG65) HH21-O (Ligand)	2.32		(GLY442) CA-O (Ligand)	3.51
	(ARG65) HH21-H (Ligand)	2.66		(TYR196) Pi-Alkyl-C (Ligand)	5.24
	(TYR68) HH-H (Ligand)	2.39		(PHE199) Pi-Alkyl-C (Ligand)	4.90
	(ASN38) HD22 -O (Ligand)	2.70		(PHE216) Pi-Alkyl-C (Ligand)	4.56
**1T2P-Rutin**	(ASN38) HD21-H (Ligand)	1.01	**1X0U-Trilobolide**	(ALA175) Alkyl-C (Ligand)	3.99
	(GLU129) OE1 -Pi interaction	4.59		(THR438) HG1-O (Ligand)	2.56
	(LYS146) OXT-HC (Ligand)	2.43		(TYR372) HN-O (Ligand)	2.24
	(TYR127) CB-Pi-Sigma interaction	3.63		(GLY371) CA-O (Ligand)	3.12

	(ASP126) HN-O (Ligand)	3.09	3FHU-Pheophorbide a	(ALA63) O-H (Ligand)	2.44
	(ASP126) OD2-H (Ligand)	2.46		(THR40) CB-O (Ligand)	3.57
	(ASP64) OD1-H (Ligand)	1.96		(TRP66) Pi-Pi interaction (Ligand)	5.40
	(ASP125) OD1-H (Ligand)	2.13		(TRP66) Pi-Alkyl interaction	4.94
1T2P-Sciadopitysin	(ASN38) HD21-O (Ligand)	1.85		(GLN72) HE22-O (Ligand)	2.40
	(ARG65) HH21-H (Ligand)	2.03		(GLN72) OE1-H (Ligand)	2.31
	(PHE62) Pi-Pi interaction	5.46		(GLN72) OE1-HN (Ligand)	2.31
	(PHE62) CA -O (Ligand)	3.10		(GLN72) OE1-Pi interaction	2.00
	(ILE63) HN-O (Ligand)	2.54		(GLN72) OE1-HN (Ligand)	2.16
	(ASP125) OD1-Pi interaction	3.89		(GLN72) OE1-HN (Ligand)	2.29
	(ASP125) OD1-Pi interaction	4.63		(VAL74) Pi-Alkyl interaction	5.02
	(ASP125) OD2-H (Ligand)	2.41		(LYS95) Alkyl-C (Ligand)	4.30
	(TYR15) Pi-Pi interaction	5.90	3FHU-Pyropheophorbide a	(ALA76) Alkyl-C (Ligand)	3.69
	(TYR15) Pi-Pi interaction	5.56		(VAL74) Alkyl-C (Ligand)	3.96
	(ASP126) OD2-Pi interaction	4.86		(THR92) HG1-Pi interaction	3.10
	(ASP126) OD2-Pi interaction	4.00		(VAL151) Alkyl-C (Ligand)	4.73
	(PRO26) Alkyl-C (Ligand)	4.36		(ASN94) OD1-H (Ligand)	2.96
1T2P-Thalidasine	(LYS85) HZ3-O (Ligand)	2.29		(THR149)HG1 (Ligand)	3.11

	(TYR15) HH-O (Ligand)	2.79		(THR149) OG1-H (Ligand)	1.74
	(TYR15) Pi-Sigma-CC (Ligand)	3.75		(GLY81) HN-O (Ligand)	2.89
	(LYS24) Alkyl-CC (Ligand)	4.66		(GLY84) O-H (Ligand)	2.14
	(LYS24) Pi-Alkyl Interaction	5.48		(ASN86) HD22- Pi interaction	2.37
	(PRO26) Pi-Alkyl Interaction	4.83		(ASP78) OD2-H (Ligand)	2.50
	(TYR15) Pi-Pi interaction	5.50	**3FHU-Sciadopitysin**	(VAL151) CG1- Pi interaction	3.82
				(VAL151) CG2- Pi-Sigma interaction	3.57
	(GLY412) HN-O (Ligand)	2.10		(LYS118) Alkyl-C (Ligand)	4.94
	(GLY412) HN-O (Ligand)	2.12		(ASN120) O-C (Ligand)	3.78
	(THR438) OG1-OC (Ligand)	2.96		(THR92) HG1-H (Ligand)	1.30
	(GLY439) HN-O (Ligand)	2.17		(THR92) HG1-O (Ligand)	2.37
**1X0U-1,4-Dimethoxyglucobrassicin**	(GLU204) OE2-C (Ligand)	3.68			
	(GLU204) OE2-Pi interaction	3.56		(LYS114) NZ-Pi interaction	4.06
	(ALA175) Pi-Alkyl interaction	5.18	**3SY7-Artomunoxanthentrioneepoxide**	(TYR68) HH-O (Ligand)	2.43
	(ALA175) Alkyl-C (Ligand)	4.16		(THR49) HG1-O (Ligand)	2.08
	(PHE216) Pi-AlkyL-C (Ligand)	4.97		(SER200) HG-Pi interaction	2.62

	(PHE199) Pi-Alkyl-C (Ligand)	4.95	**3SY7-N-(6-Oxo-6H-dibenzo[b,d]pyran-3-yl)maleamicacid**	(PRO123) Pi-Alkyl interaction	4.99
	(ILE446) Pi-Alkyl interaction	5.19		(ARG190) Pi-Alkyl interaction	5.32
**1X0U-3,4',5,6,8-Pentamethoxyflavone**	(TYR372) HN-O (Ligand)	2.41		(TYR214) HH-O (Ligand)	2.39
	(PRO370) Alkyl-C (Ligand)	5.03		(SER198) CB-O (Ligand)	3.53
	(ILE205) CG2-Pi-Sigma interaction	3.63		(SER198) HN-O (Ligand)	2.70
	(ILE205) Pi-Alkyl interaction	4.42		(ASN218) OD1-H (Ligand)	2.20
	(ILE205) Alkyl-C (Ligand)	4.03	**3SY7-Petasitenine**	(PHE133) HN-O (Ligand)	2.23
	(ALA175) Alkyl-C (Ligand)	4.13		(LEU132) HN-H (Ligand)	2.50
	(THR201) OG1-O (Ligand)	2.90		(ASP96) O-C (Ligand)	3.76
**1X0U-Afzelechin**	(GLU396) OE2-H (Ligand)	1.94	**3SY7-Pheophorbidea**	(ASN218) O-H (Ligand)	2.18
	(LYS515) O-H (Ligand)	2.66		(ASN218) HD21- Pi interaction	3.18
	(LYS515) Pi-Alkyl interaction	4.12		(ALA259) Alkyl-C (Ligand)	3.74
	(LYS515) O-C (Ligand)	3.62		(PHE298) Pi-Alkyl-C (Ligand)	4.86
	(LYS515) Pi-Alkyl interaction	4.34		(ASP232) OD2- Pi interaction	4.17
	(ARG512 Pi-Alkyl interaction	5.23		(ASP232) OD1- Pi interaction	3.17
**1X0U-4-(3-Hydroxy-7-phenyl-6-**	(GLU396) OE2-H (Ligand)	2.33		(ASP232) OD2- Pi interaction	4.88

**heptenyl)-1,2-benzenediol**	(LYS515) O-H (Ligand)	2.65		(SER200) HG-O (Ligand)	2.32
	(LYS515) NZ-Pi interaction	4.87	**3SY7-PyrafolineD**	(PHE409) Pi-Pi interaction (Ligand)	5.60
	(LYS515) HZ3-Pi interaction	2.81		(PHE409) Pi-Pi interaction (Ligand)	4.19
	(ARG512) Pi-Alkyl interaction	4.87		(PHE409) Pi-Pi interaction (Ligand)	3.82
**1X0U-Artomunoxanthentrione epoxide**	(GLY411) CA-O (Ligand)	3.67		(PHE409) Pi-Alkyl interaction	5.32
**1X0U-Lactucin**	(GLY177) HN-O (Ligand)	2.57		(TYR27) Pi-Alkyl interaction	4.24
	(GLY412) HN-O (Ligand)	2.36		(TYR27) Pi-Alkyl-C (Ligand)	4.92
	(GLY137) CA-O (Ligand)	3.46		(LEU411) Alkyl-Alkyl interaction (Ligand)	5.27
**1X0U-LinifolinA**	(ALA138) HN-O (Ligand)	2.22		(LEU411) Alkyl -C (Ligand)	4.94
	(GLY177) HN-O (Ligand)	2.04		(TRP42) Pi-Sigma-C (Ligand)	3.79
	(THR438) HG1-O (Ligand)	2.50		(TRP42) Pi-Alkyl-C (Ligand)	4.09
**1X0U-N-(6-Oxo-6H-dibenzo[b,d]pyran-3-yl)maleamicacid**	(TYR392) HH-O (Ligand)	2.22		(TRP42) Pi-Alkyl-C (Ligand)	4.45
	(GLY518) HN-O (Ligand)	2.34	**3SY7-Pyropheophorbide a**	(GLN119) OE1-HN (Ligand)	2.82
	(HIS517) CA-O (Ligand)	3.35		(GLN119) OE1-HN (Ligand)	2.54
	(TYR513) O-H (Ligand)	2.78		(GLN119) OE1- Pi interaction (Ligand)	2.45

	(LYS515) O-H (Ligand)	2.29		(GLN119) OE1-HN(Ligand)	2.64
	(LYS515) Pi-Alkyl interaction	4.16		(GLN119) OE1-H(Ligand)	2.68
	(LYS515) NZ-Pi interaction	4.43		(LYS114) HZ1-O (Ligand)	2.16
	(LYS515) NZ-Pi interaction	3.62		(LEU22) Alkyl-Alkyl interaction (Ligand)	5.22
	(ARG512) Pi-Alkyl interaction	4.86		(LEU47) Alkyl-Alkyl interaction (Ligand)	4.88
**1X0U-N6-cis-p-Coumaroylserotonin**	(TYR392) HH-O (Ligand)	2.66		(PRO134) Pi-Alkyl interaction	4.12
	(LYS515) NZ-Pi interaction	4.10		(PRO134) Alkyl-Alkyl interaction (Ligand)	4.51
	(LYS515) Pi-Alkyl interaction	4.62	**3SY7-Rutin**	(TYR97) Pi-Pi interaction (Ligand)	5.04
	(ARG512) Pi-Alkyl interaction	4.31		(TYR97) Pi-Pi T-shaped interaction (Ligand)	5.00
	(LYS516) O-H (Ligand)	2.36		(PHE133) Pi-Pi T-shaped interaction (Ligand)	4.83
	(LYS515) NZ-Pi interaction	3.94		(PHE133) CB-Pi- Sigma interaction (Ligand)	3.87
	(ARG512) Pi-Alkyl interaction	4.95		(PHE133) HN-O (Ligand)	2.48
**1X0U-Petasitenine**	(GLY439) HN-O (Ligand)	2.97		(LEU132) Pi-Alkyl interaction (Ligand)	5.23

	(GLY411) CA-O (Ligand)	3.44		(LEU132) HN-O (Ligand)	2.82
	(GLY202) HN-O (Ligand)	2.07		(ARG131) HH22-O (Ligand)	2.19
	(THR201) CA-O (Ligand)	3.35		(ARG131) HE-H (Ligand)	1.92
	(GLU204) OE1-C (Ligand)	3.49		(ASP295) OD2-H (Ligand)	2.39
**1X0U-Pheophorbidea**	(PRO282) O-H (Ligand)	1.94		(ARG410) HH11-O (Ligand)	2.45
	(ARG470) HE-O (Ligand)	2.43		(GLY293) O-H (Ligand)	2.49
	(ARG470) HH22-O (Ligand)	2.21		(SER130) O-C (Ligand)	3.49
	(TYR477) Pi Alkyl-C (Ligand)	4.98		(ARG39) HH21-H (Ligand)	2.53
	(LEU11) Pi-Alkyl interaction	5.47	**3SY7-Sciadopitysin**	(ALA300) O-C (Ligand)	3.53
	(LYS5) HT2- Pi interaction (Ligand)	3.12		(PHE298) Pi-Pi T-shaped interaction (Ligand)	5.66
	(LYS5) N- Pi interaction (Ligand)	3.88		(ASP232) OD1- Pi interaction (Ligand)	4.30
	(LYS5) CO-(PRO6) N-Amide-Pi interaction	4.74		(ASN218) HD22-O (Ligand)	3.03
	(PRO6) Pi-Alkyl interaction	4.84		(TYR214) HH-Pi interaction (Ligand)	3.12
	(LYS471) Pi-Alkyl interaction	4.44		(PRO123) Alkyl-C (Ligand)	4.23
	(LYS471) Alkyl-C (Ligand)	3.65		(TYR202) Pi-Alkyl-C (Ligand)	4.93

	(ASP284) OD1-C (Ligand)	3.53		(ALA259) Alkyl-C (Ligand)	3.81
	(ASN283) CA-O (Ligand)	3.52		(ALA259) Pi-Alkyl interaction (Ligand)	5.21
**1X0U-PyrafolineD**	(VAL158) Alkyl-C (Ligand)	5.23		(ALA257) Alkyl-C (Ligand)	4.25
	(MET159) Alkyl-C (Ligand)	5.43		(ALA257) Pi-Alkyl interaction (Ligand)	4.69
	(TYR392) HH-O (Ligand)	2.62		(ARG319) NH1- Pi interaction (Ligand)	4.08
	(ARG512) Pi-Alkyl interaction	4.94		(ARG319) HH21-O (Ligand)	2.05
	(ARG512) Pi-Alkyl interaction	5.19	**3SY7-Thalidasine**	(GLY293) O-C (Ligand)	3.74
	(ARG512) Pi-Alkyl interaction	5.22		(PHE28) Pi-Alkyl-C (Ligand)	4.15
	(LYS515) NZ-Pi interaction	3.70		(TYR26) Pi-Sigma-C (Ligand)	3.90
	(LYS515) Pi-Alkyl interaction	4.17		(LEU132) Alkyl-C (Ligand)	3.82
	(LYS515) Pi-Alkyl interaction	4.70		(ASP41) OD2-C (Ligand)	3.27
	LYS516) Alkyl-C (Ligand)	4.03		(ASP41) OD2-Pi interaction (Ligand)	4.44
	ARG512) Alkyl-C (Ligand)	3.69		(TYR97) Pi-Pi interaction (Ligand)	4.06
**1X0U-Pyropheophorbide a**	VAL209) Pi-Alkyl interaction	4.72	**1X0U-Pyropheophorbide a**	PRO334) O-H (Ligand)	2.37

	VAL209) Pi-Alkyl interaction	5.14		ARG407) HH12-O (Ligand)	2.37
	ILE205) Alkyl-Alkyl interaction	4.97		LYS408) HZ1-O (Ligand)	2.07
	PRO370) Pi-Alkyl interaction	4.89		PHE337) C,O;(GLY338) N-Amide Pi interaction (Ligand)	4.21
	PRO370) Pi-Alkyl interaction	4.51		PHE337) Pi-Alkyl- C (Ligand)	5.33
	PRO370) Pi-Alkyl interaction	4.95		VAL373) Alkyl-C (Ligand)	3.68
