# Supplementary material for: Anti-Adhesion and Antibiofilm Activity of Eruca sativa Miller Extract Targeting Cell Adhesion Proteins of Food-Borne Bacteria as a Potential Mechanism: Combined In Vitro-In Silico Approach
Source: Plants (Basel). 2022 Feb 24;11(5):610. doi: 10.3390/plants11050610 (PMC8912376; doi:10.3390/plants11050610)
Supplement: Supplementary file 1 [file plants-11-00610-s001.zip › Supplementary File (S1).pdf]

Positive Analysis

1. (+/-)-3-[(2-methyl-3- furyl)thio]-2-butanone

Compound Label	Name	*m/z*	RT	Algorithm	Mass
Cpd 1: (+/-)-3-[(2-methyl-3-furyl)thio]-2-butanone	**(+/-)-3-[(2-methyl-3-furyl)thio]-2-butanone**	185.0649	0.816	Auto MS/MS	184.0576

MS Spectrum

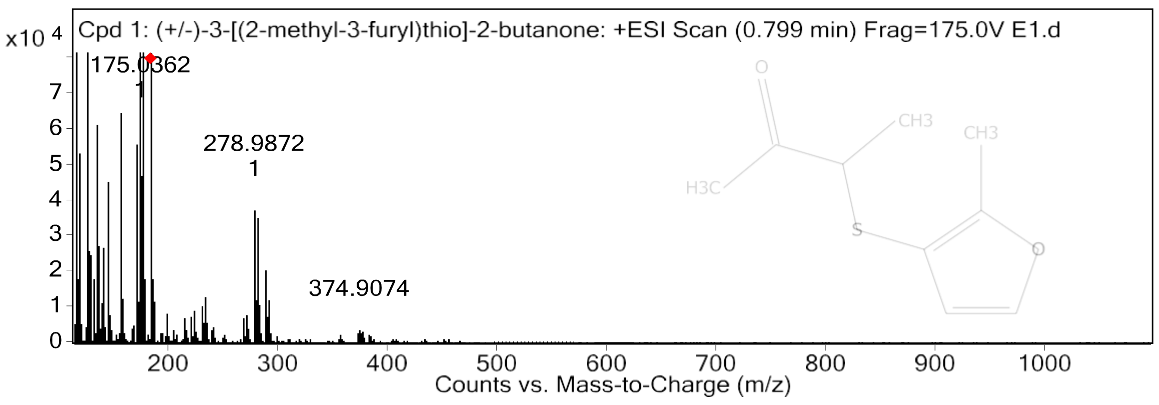

MS Zoomed Spectrum

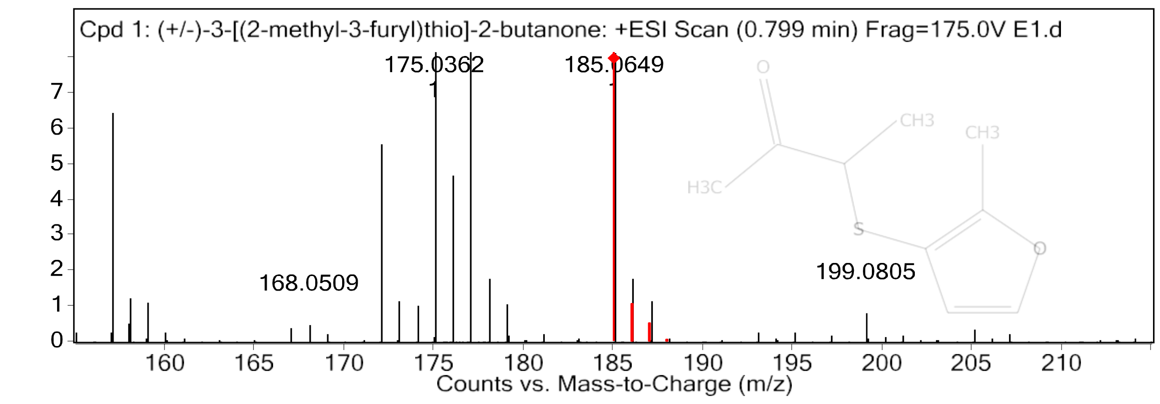

MS Spectrum Peak List

m/z	Calc m/z	Diff(ppm)	z	Abund	Formula	Ion
116.9947			1	110012.21		
127.0232			1	158665.36		
135.005			1	61114.48		
157.0701			1	64821.2		
175.0362			1	209358.02		
177.0334			1	111970.17		
185.0649	185.0631	-9.94	1	81394.4	C9 H12 O2 S	(M+H)+
186.0666	186.0662	-2.07	1	17901.9	C9 H12 O2 S	(M+H)+
187.0636	187.0605	-16.45	1	11618.34	C9 H12 O2 S	(M+H)+
188.0677	188.0633	-23.31	1	861.09	C9 H12 O2 S	(M+H)+

MSMS Spectrum

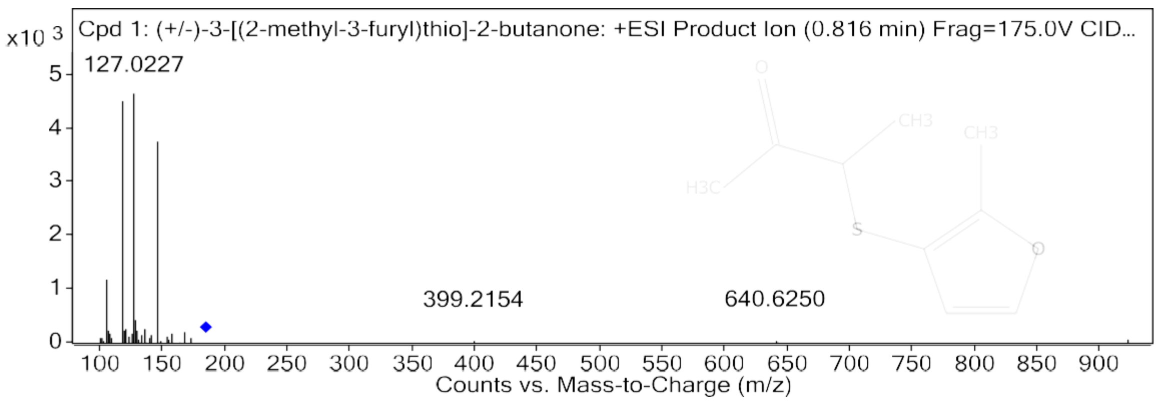

MS/MS Spectrum Peak List

m/z	z	Abund
105.0023	1	1196.47
117.039	1	4526.39
118.041	1	620.45
119.5702		267.18
127.0227	1	4663.13
128.0243	1	437.18
129.0214	1	256.6
135.0051		290.05
145.0336	1	3758.91
146.0345	1	312.31

Compound Structure

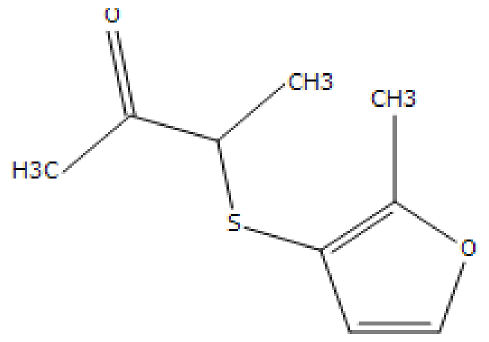

2. Methyl N-methylanthranilate

Compound Label	Name	*m/z*	RT	Algorithm	Mass
Cpd 8: Methyl N-methylanthranilate	**Methyl N-methylanthranilate**	166.0852	2.06	Auto MS/MS	165.078

MS Spectrum

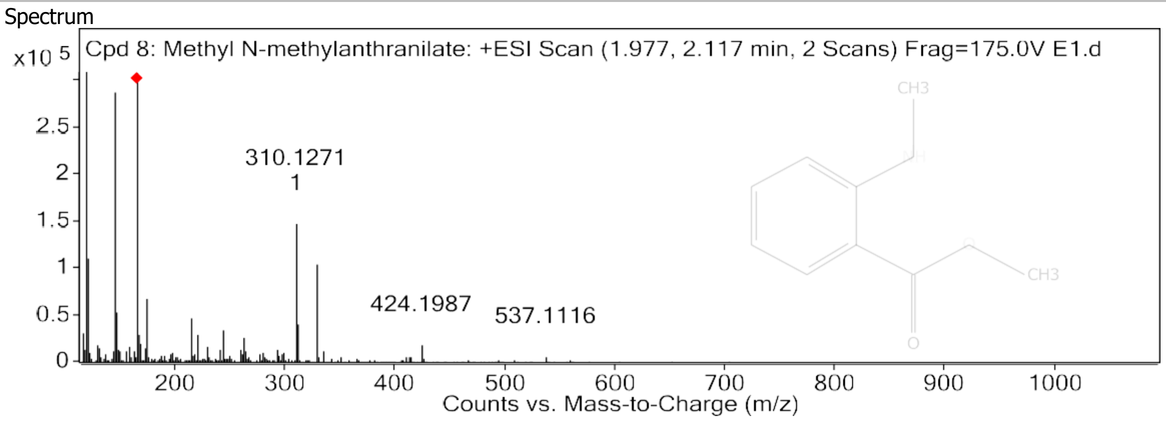

MS Zoomed Spectrum

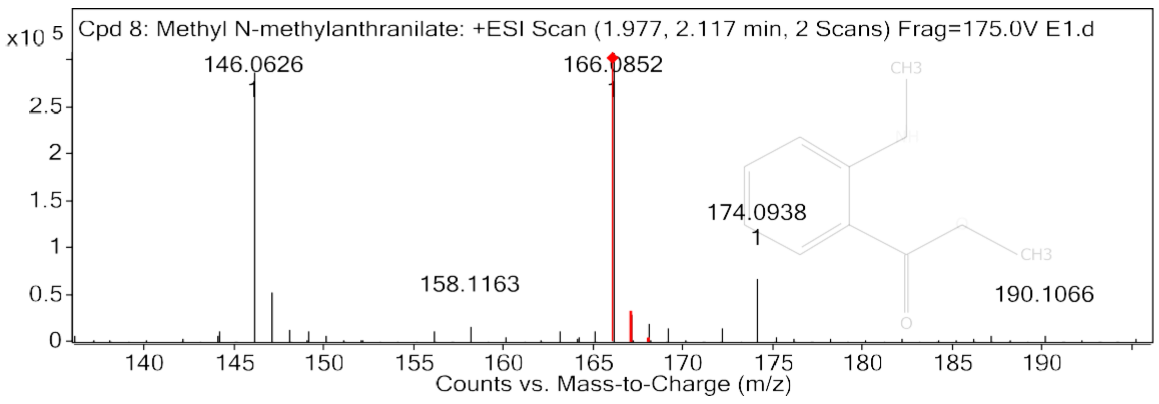

MS Spectrum Peak List

MSMS Spectrum

m/z	Calc m/z	Diff(ppm)	z	Abund	Formula	Ion
119.0518				56329.89		
120.0802			1	1174467.5		
121.0835			1	111715.97		
146.0626			1	288672.16		
166.0852	166.0863	6.32	1	308575.47	C9 H11 N O2	(M+H)+
167.0886	167.0894	4.93	1	30060.38	C9 H11 N O2	(M+H)+
168.0931	168.0916	-8.76	1	3461.47	C9 H11 N O2	(M+H)+
174.0938			1	67972.09		
310.1271			1	147865.09		
328.1377			1	105372.38		

MS/MS Spectrum Peak List

m/z	z	Abund
103.0536	1	362.83
104.1058		90.32
120.0799	1	3076.68
121.083	1	329.44
146.063		266.01
147.0478		146.39
156.0992	1	83.49
163.0855		82.34
166.0835		319.03
708.2548		88.55

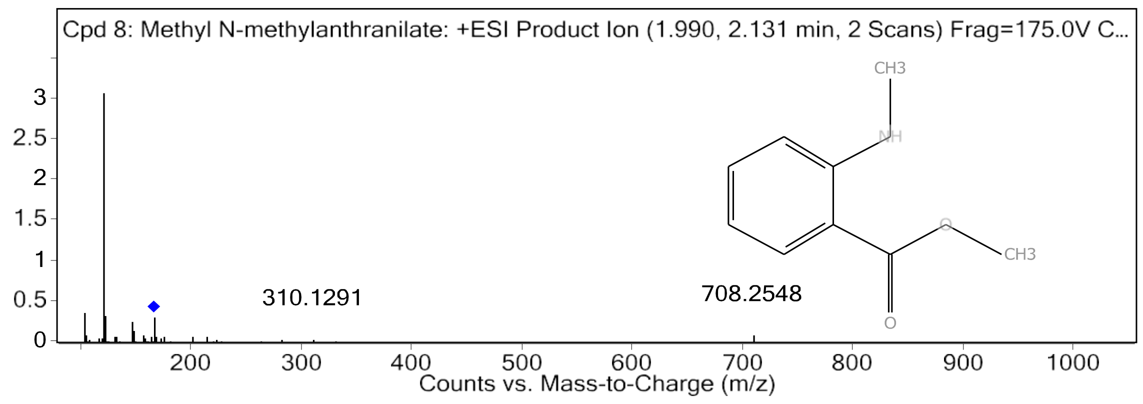

Compound Structure

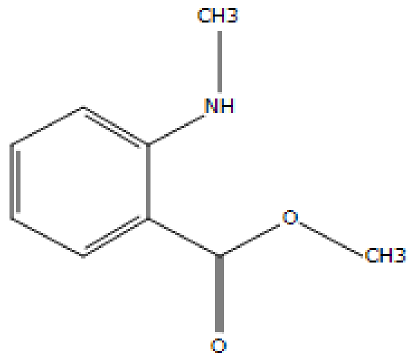

3. Indoleacrylic acid

Compound Label	Name	*m/z*	RT	Algorithm	Mass
Cpd 12: Indoleacrylic acid	**Indoleacrylic acid**	188.0696	3.189	Auto MS/MS	187.0623

MS Spectrum

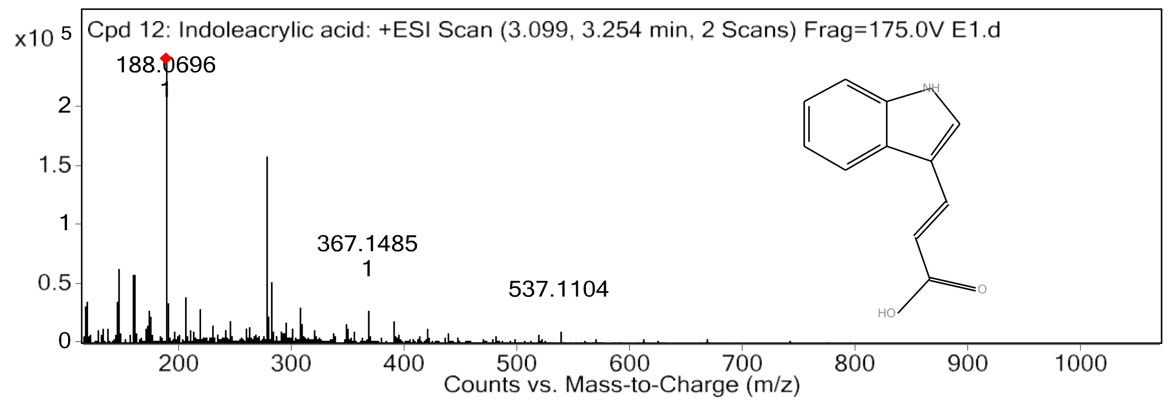

MS Zoomed Spectrum

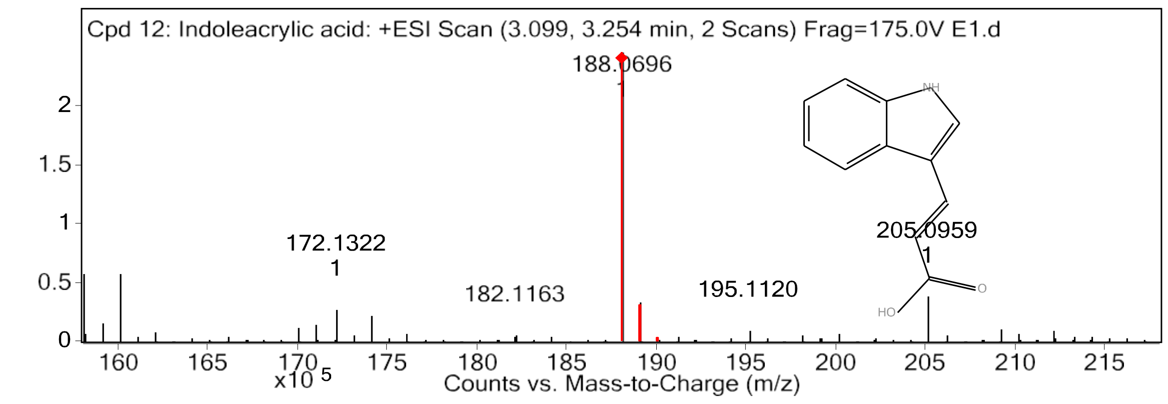

MS Spectrum Peak List

m/z	Calc m/z	Diff(ppm)	z	Abund	Formula	Ion
146.0599			1	63107.44		
158.0802			1	58685.86		
160.0779			1	58120.28		
188.0696	188.0706	5.31	1	245927.19	C11 H9 N O2	(M+H)+
189.0724	189.0738	7.46	1	33734.57	C11 H9 N O2	(M+H)+
190.0769	190.0762	-3.65	1	2726.04	C11 H9 N O2	(M+H)+
205.0959			1	38747.41		
277.1203			1	158999.75		
277.1557			1	76581.17		
281.159			1	52007.66		

MSMS Spectrum

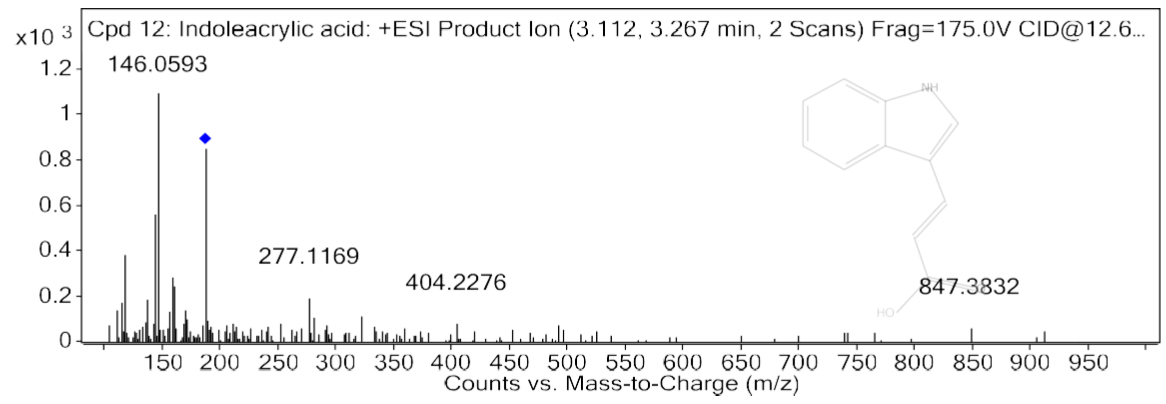

MS/MS Spectrum Peak List

m/z	z	Abund
115.0519		180.68
118.0642		388.08
137.105		193.65
143.0726		235.59
144.0793		565.64
146.0593	1	1102.29
158.0803		292.75
160.0726	1	247.65
188.0688	1	858.54
277.1169		198.72

Compound Structure

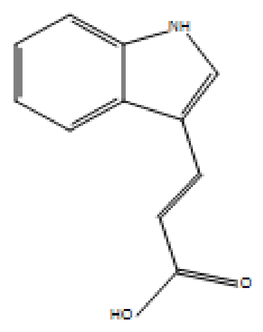

4. Pyrafoline D

Compound Label	Name	*m/z*	RT	Algorithm	Mass
Cpd 15: Pyrafoline D	**Pyrafoline D**	348.2001	3.705	Auto MS/MS	347.1928

MS Spectrum

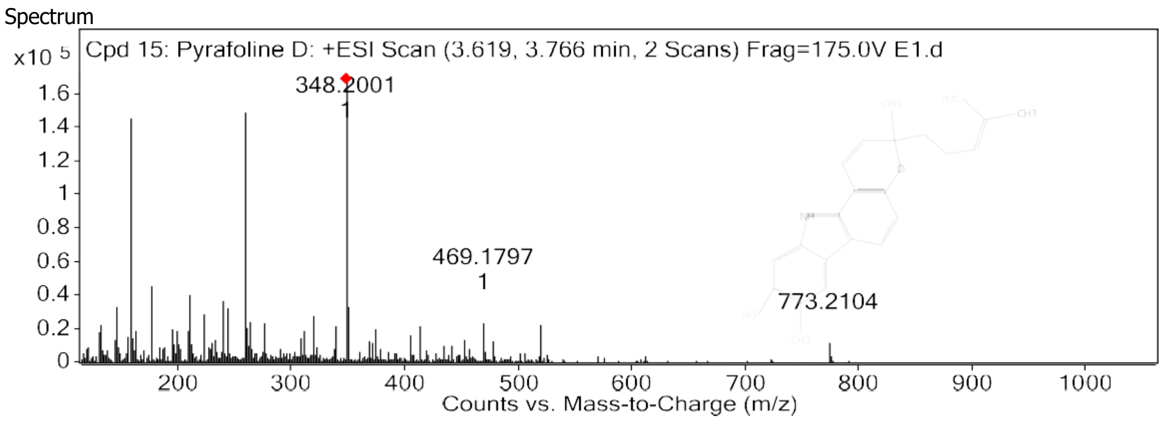

MS Zoomed Spectrum

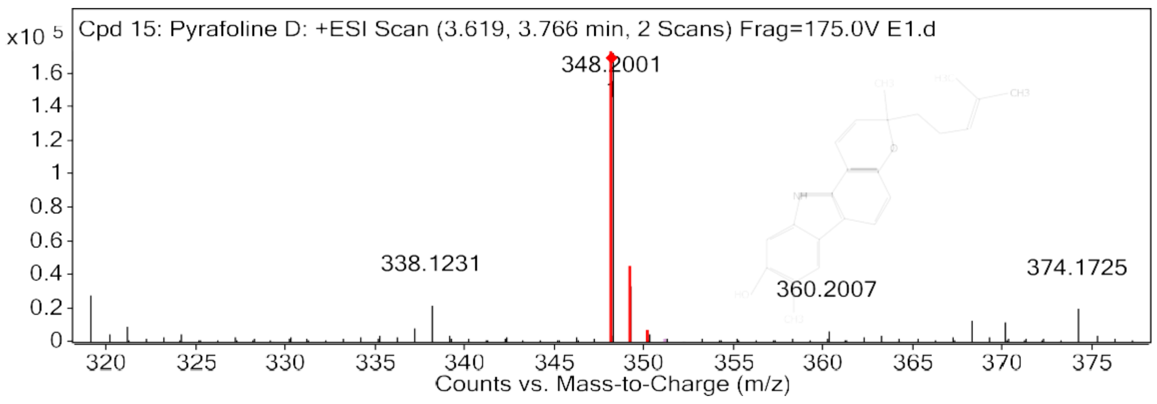

MS Spectrum Peak List

m/z	Calc m/z	Diff(ppm)	z	Abund	Formula	Ion
146.0604				33641.77		
158.0805			1	146882.72		
176.0695				46682.13		
210.1112				41401.32		
240.071				37430.91		
244.153				33074.08		
259.1096			1	149736.44		
348.2001	348.1958	-12.45	1	173245.17	C23 H25 N O2	(M+H)+
349.2035	349.1991	-12.5	1	34117.08	C23 H25 N O2	(M+H)+
350.2029	350.2021	-2.1	1	5685.56	C23 H25 N O2	(M+H)+

MSMS Spectrum

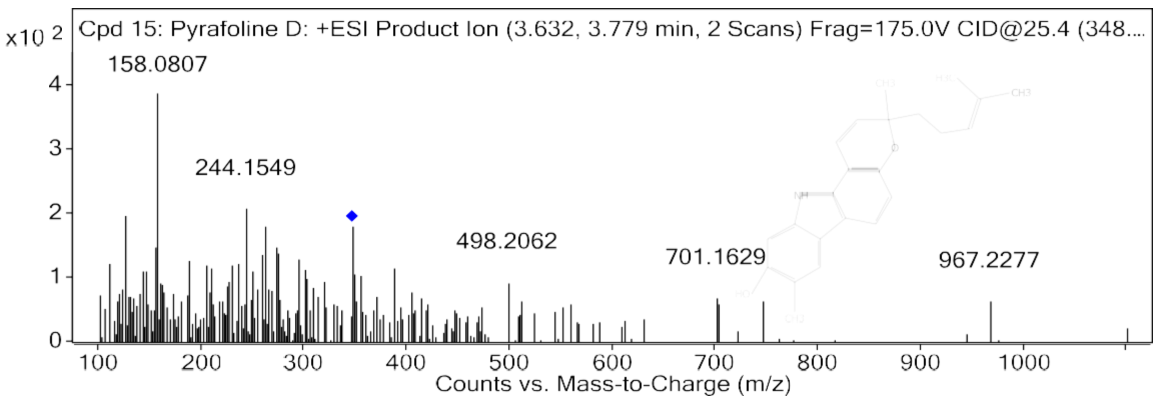

MS/MS Spectrum Peak List

m/z	Abund
127.0379	196.93
156.1001	149.07
158.0807	388.59
244.1549	208.11
259.1114	137.2
263.1368	180.15
273.1203	148.49
275.1368	139.65
295.1375	129.33
348.1976	180.69

Compound Structure

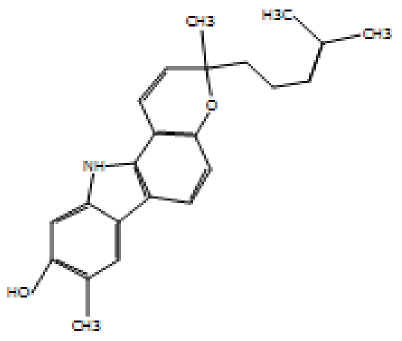

5. Petasitenine

Compound Label	Name	*m/z*	RT	Algorithm	Mass
Cpd 20: Petasitenine	**Petasitenine**	382.1846	4.232	Auto MS/MS	381.1773

MS Spectrum

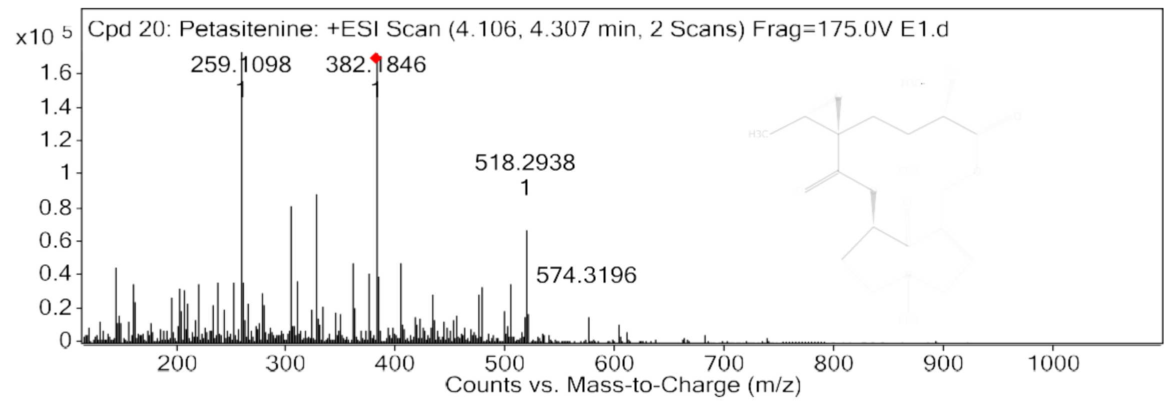

MS Zoomed Spectrum

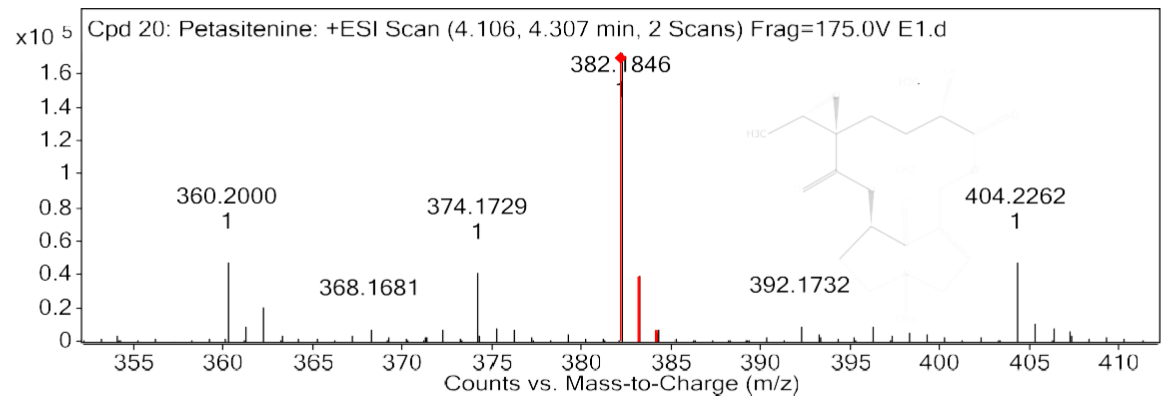

MS Spectrum Peak List

m/z	Calc m/z	Diff(ppm)	z	Abund	Formula	Ion
144.0797				45903.2		
259.1098			1	242151.56		
303.1326			1	82336.95		
327.1029			1	89596.5		
360.2			1	48061.79		
382.1846	382.186	3.77	1	173587.88	C19 H27 N O7	(M+H)+
383.1878	383.1893	3.93	1	40120.95	C19 H27 N O7	(M+H)+
384.19	384.1917	4.53	1	8303.24	C19 H27 N O7	(M+H)+
404.2262			1	47920.5		
518.2938			1	68385.55		

MSMS Spectrum

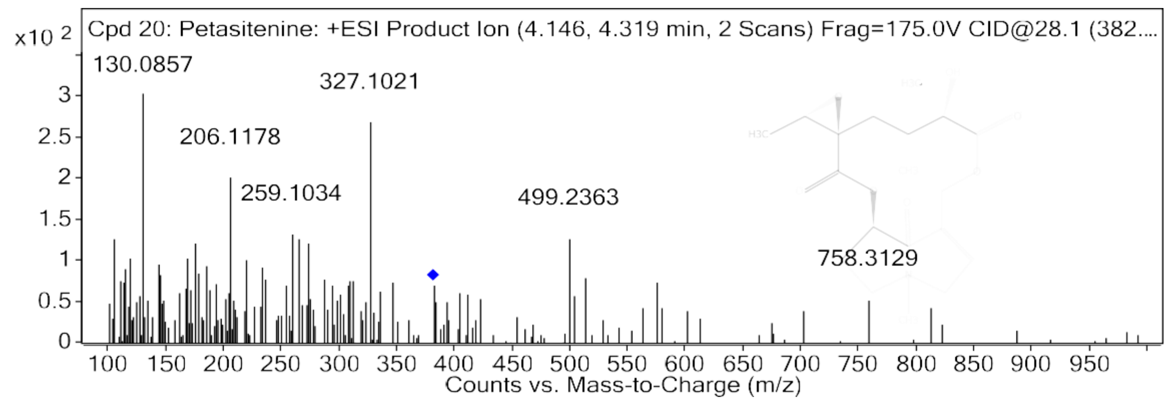

MS/MS Spectrum Peak List

m/z	Abund
105.0698	128.07
130.0857	304.6
168.1006	103.73
175.0748	121.44
206.1178	201.29
259.1034	132.65
264.1551	128.11
273.1219	121.65
327.1021	268.63
499.2363	127.54

Compound Structure

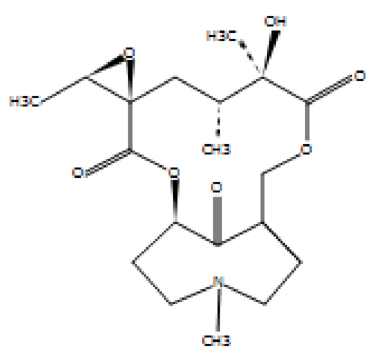

6. Nopaline

Compound Label	Name	*m/z*	RT	Algorithm	Mass
Cpd 23: Nopaline	**Nopaline**	305.152	5.05	Auto MS/MS	304.1444

MS Spectrum

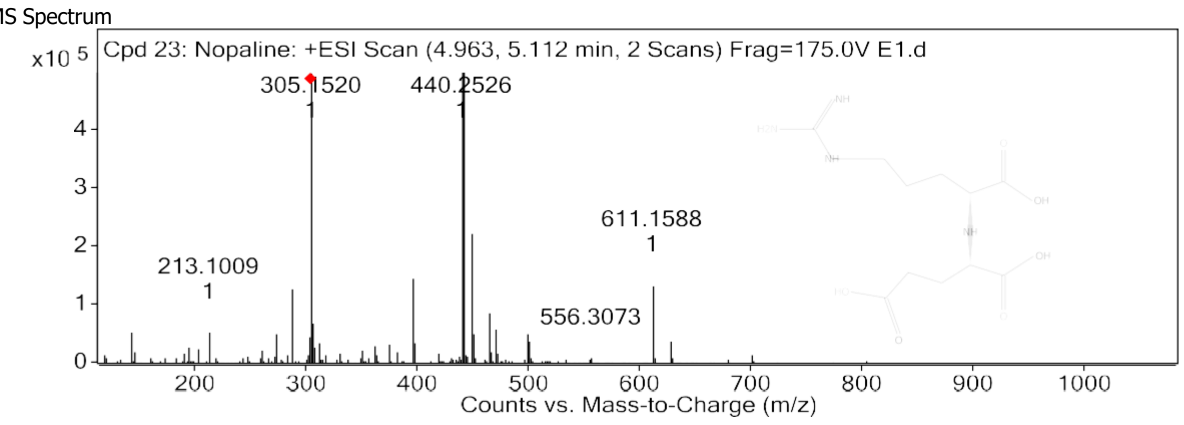

MS Zoomed Spectrum

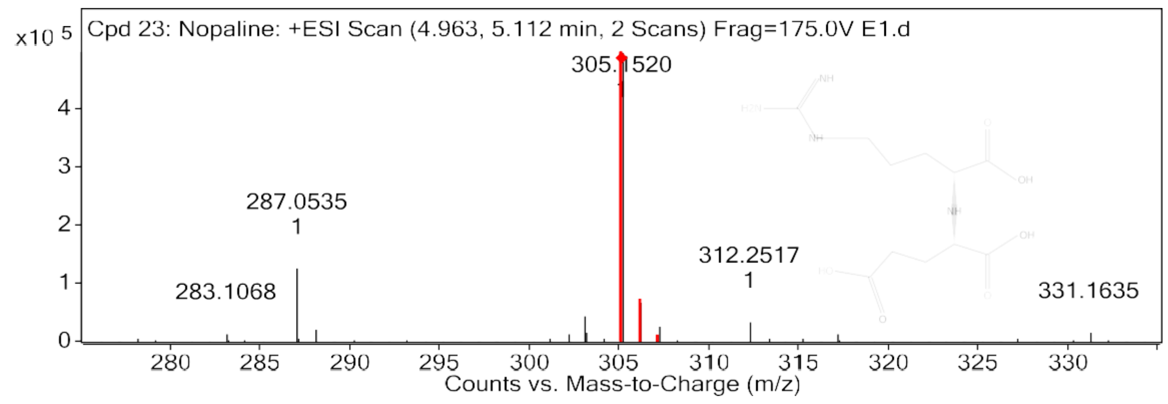

MS Spectrum Peak List

m/z	Calc m/z	Diff(ppm)	z	Abund	Formula	Ion
287.0535			1	128043.7		
305.152	305.1456	-20.99	1	497112.03	C11 H20 N4 O6	(M+H)+
306.1546	306.1483	-20.44	1	69755.84	C11 H20 N4 O6	(M+H)+
307.1505	307.1503	-0.68	1	27771.19	C11 H20 N4 O6	(M+H)+
396.1999			1	145300.48		
440.2526			1	2542112		
441.2558			1	684534.38		
442.2592			1	108497.92		
449.1061			1	224013.75		
611.1588			1	134280.39		

MSMS Spectrum

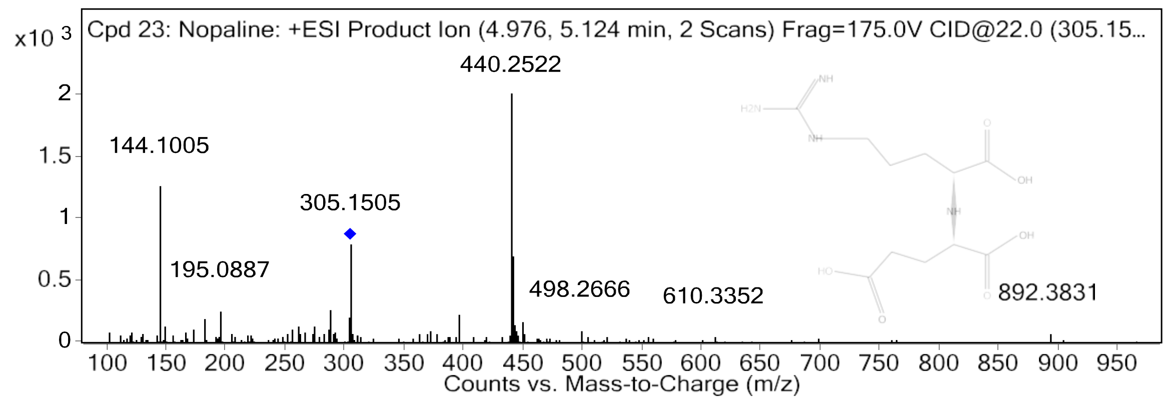

MS/MS Spectrum Peak List

m/z	z	Abund
144.1005		1259.69
182.1536		193.97
195.0887		254.36
287.0534		268.14
304.0525		202.63
305.1505	1	796.83
396.1993		232.85
440.2522	1	2015.27
441.2547	1	695.31
449.1042	1	167.43

Compound Structure

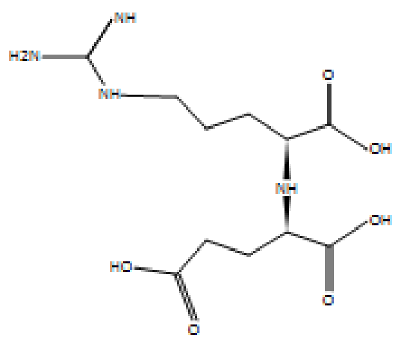

7. Serinyl-Hydroxyproline

Compound Label	Name	*m/z*	RT	Algorithm	Mass
Cpd 25: Serinyl-Hydroxyproline	**Serinyl-Hydroxyproline**	219.0974	5.443	Auto MS/MS	218.0894

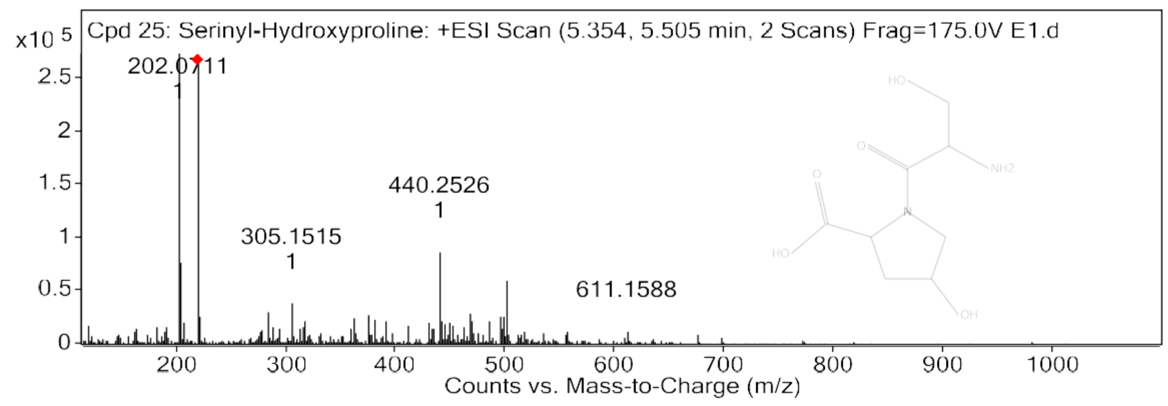

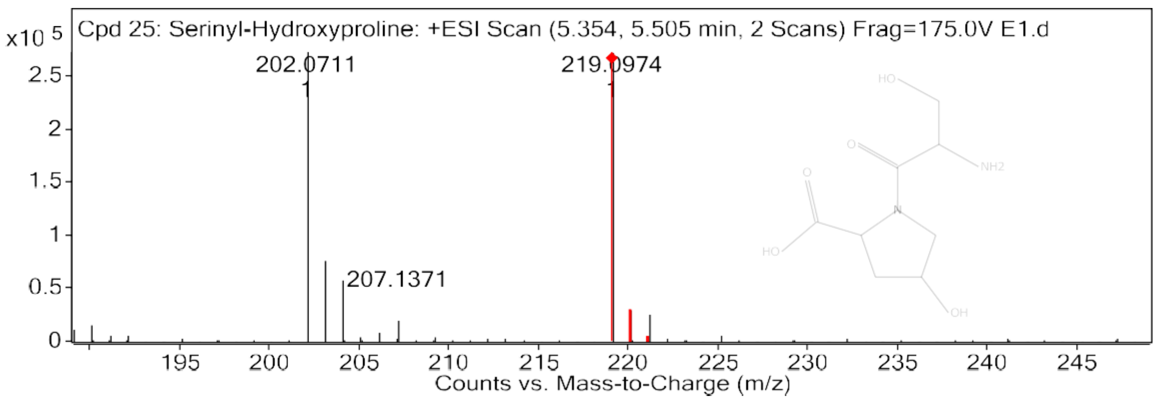

MS Spectrum

MS Zoomed Spectrum

MS Spectrum Peak List

m/z	Calc m/z	Diff(ppm)	z	Abund	Formula	Ion
202.0711			1	671245.63		
203.0739			1	77077.11		
204.0669			1	58465.06		
219.0974	219.0975	0.68	1	272534.03	C8 H14 N2 O5	(M+H)+
220.1003	220.1005	0.84	1	31596.74	C8 H14 N2 O5	(M+H)+
221.0936	221.1023	39.2	1	26596.18	C8 H14 N2 O5	(M+H)+
222.0971	222.1049	35.19	1	3723.83	C8 H14 N2 O5	(M+H)+
305.1515			1	38970.19		
440.2526			1	87439.47		
500.2837			1	60787.64		

MSMS Spectrum

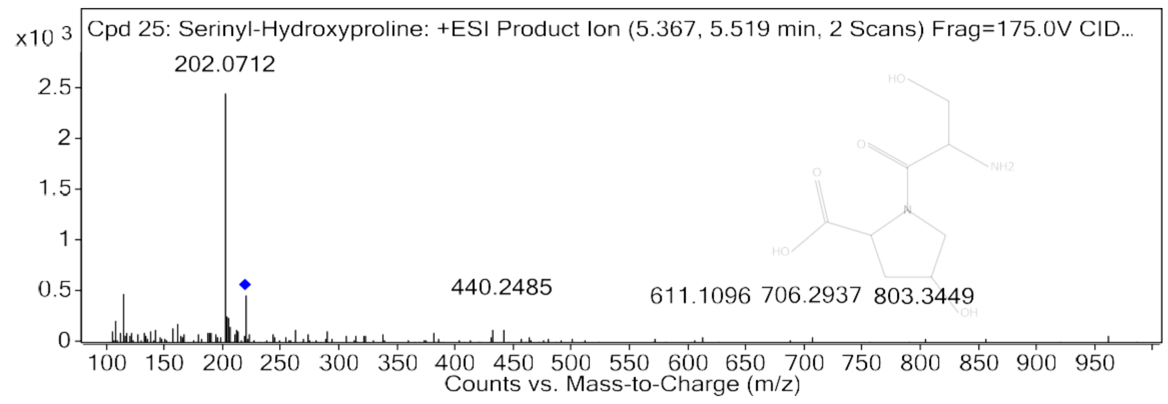

MS/MS Spectrum Peak List

m/z	z	Abund
107.084		214.26
114.0366		484.46
156.0822		144.5
160.0228		183.58
202.0712	1	2452.78
203.0746	1	263.33
204.0657	1	249.74
206.0781		156.23
219.0993		475.83
261.1173		138.41

Compound Structure

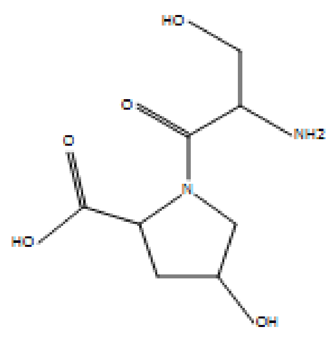

8. Afzelechin

Compound Label	Name	*m/z*	RT	Algorithm	Mass
Cpd 27: Afzelechin	**Afzelechin**	275.0873	6.004	Auto MS/MS	274.079

MS Spectrum

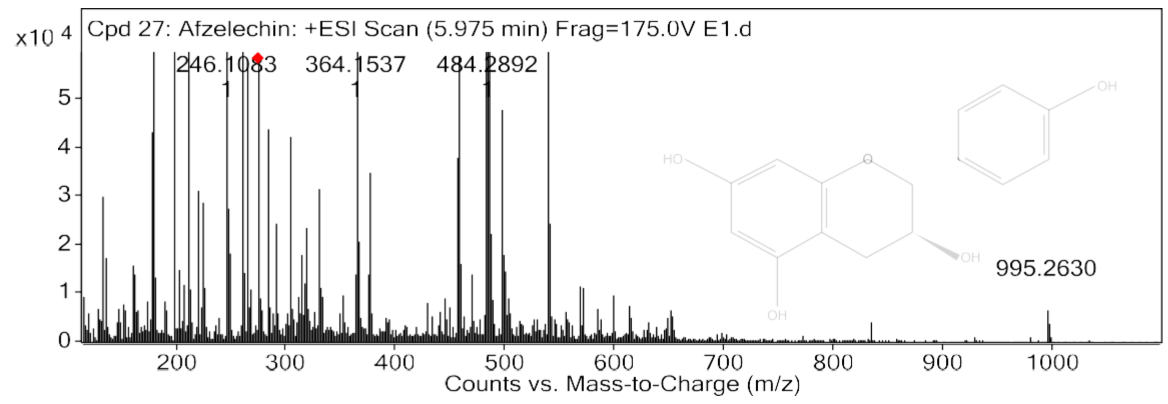

MS Zoomed Spectrum

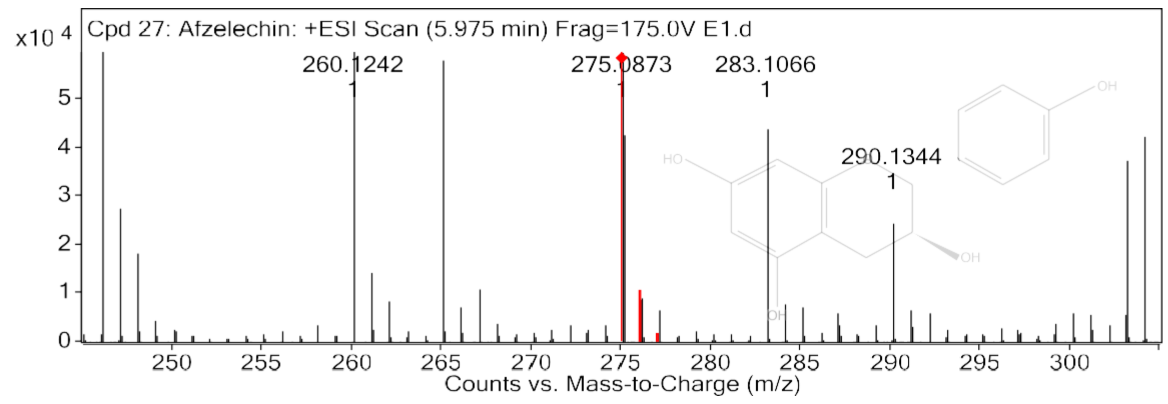

MS Spectrum Peak List

m/z	Calc m/z	Diff(ppm)	z	Abund	Formula	Ion
197.1164			1	218860.67		
246.1083			1	231836.08		
275.0873	275.0914	14.82	1	59687	C15 H14 O5	(M+H)+
276.0902	276.0948	16.56	1	8943.13	C15 H14 O5	(M+H)+
277.0827	277.0971	51.81	1	6801.76	C15 H14 O5	(M+H)+
364.1537			1	145810.13		
482.2733			1	253623.7		
484.2892			1	402137.13		
485.2924			1	112582.14		
538.2994			1	157812.19		

MSMS Spectrum

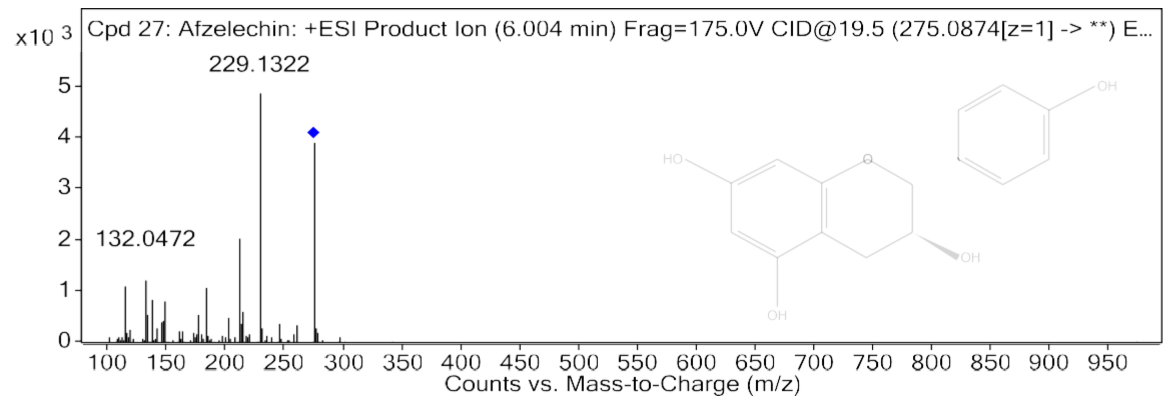

MS/MS Spectrum Peak List

m/z	z	Abund
114.0359	1	1101.94
132.0472		1223.56
137.0594		842.76
148.0734		824.45
176.0701	1	568.87
183.0936		1090.31
211.0878	1	2052.14
214.1088		616.13
229.1322	1	4885.18
275.1378	2	3902.95

Compound Structure

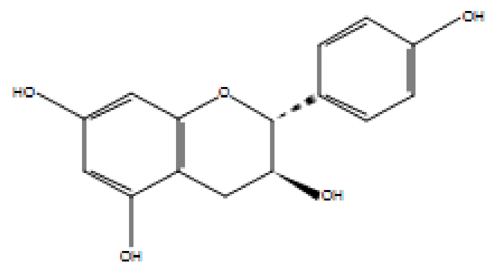

9. N-trans-Feruloyl-4-O- methyldopamine

Compound Label	Name	m/z	RT	Algorithm	Mass
Cpd 31: N-trans-Feruloyl-4-O-methyldopamine	**N-trans-Feruloyl-4-O-methyldopamine**	344.145	6.22	Auto MS/MS	343.1368

MS Spectrum

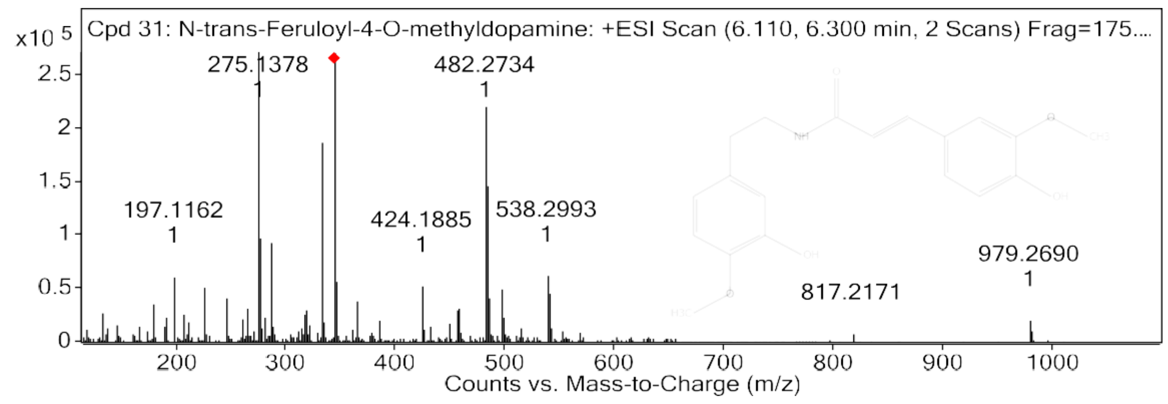

MS Zoomed Spectrum

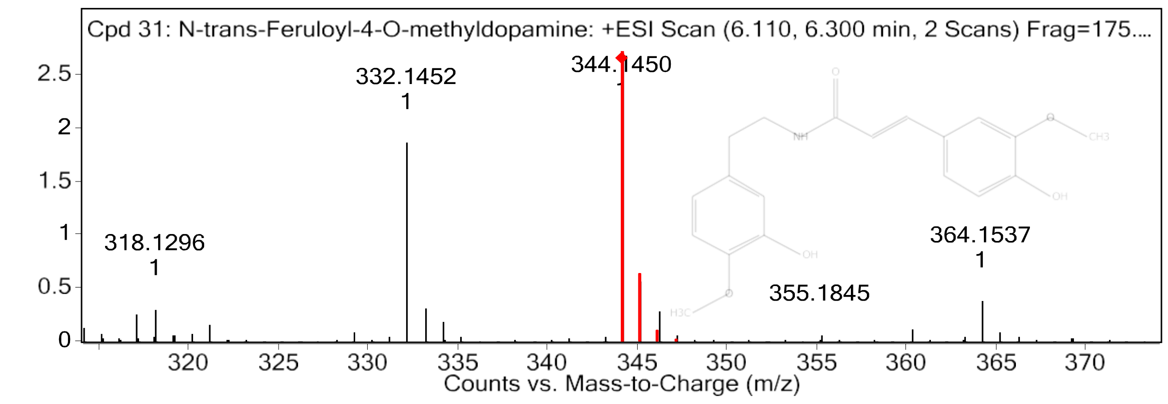

MS Spectrum Peak List

m/z	Calc m/z	Diff(ppm)	z	Abund	Formula	Ion
275.1378			1	571047.75		
276.1412			1	97709.88		
287.0537			1	93658.8		
332.1452			1	186949.41		
344.145	344.1492	12.27	1	270902.28	C19 H21 N O5	(M+H)+
345.1478	345.1525	13.86	1	56634.5	C19 H21 N O5	(M+H)+
346.1423	346.1551	36.78	1	29624.39	C19 H21 N O5	(M+H)+
347.1467	347.1577	31.56	1	6722.36	C19 H21 N O5	(M+H)+
482.2734			1	220230.81		
484.2887			1	146356.28		

MSMS Spectrum

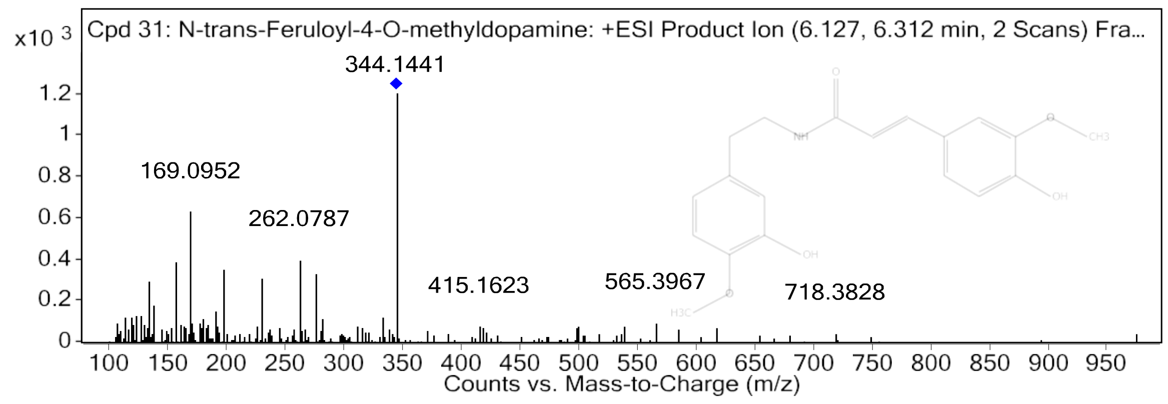

MS/MS Spectrum Peak List

m/z	z	Abund
133.101		298.63
137.0937	1	176.7
156.0867		389.06
169.0952	1	631.5
197.1251		354.49
229.0977		312.37
262.0787	1	399.99
275.136	1	328.15
344.1441	2	1205.86
345.1472	2	234.76

Compound Structure

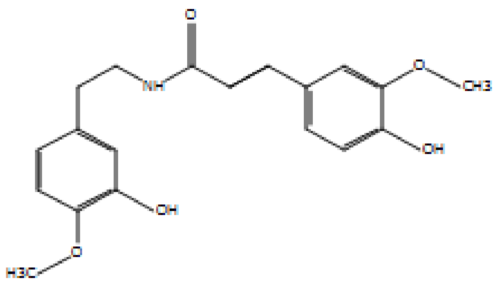

10.(±)-Rollipyrrole

Compound Label	Name	*m/z*	RT	Algorithm	Mass
Cpd 34: (±)-Rollipyrrole	**(±)-Rollipyrrole**	289.1536	6.853	Auto MS/MS	288.1463

MS Spectrum

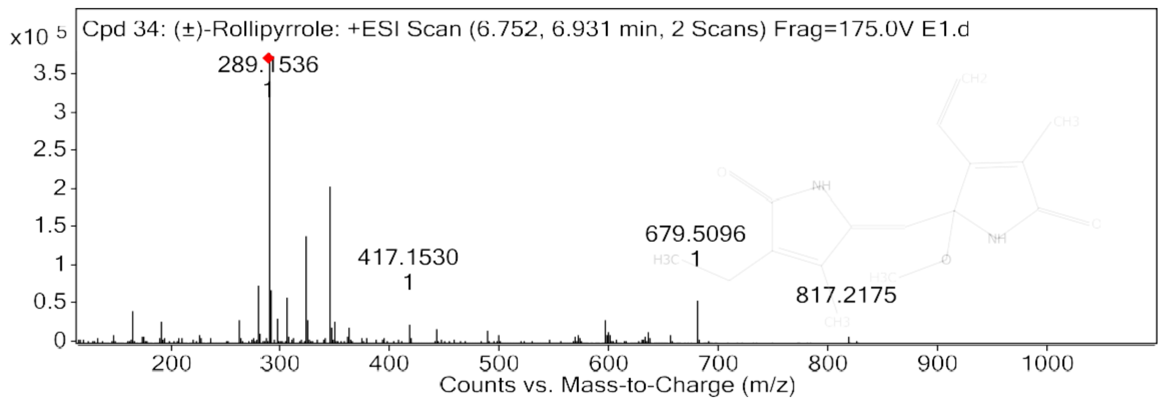

MS Zoomed Spectrum

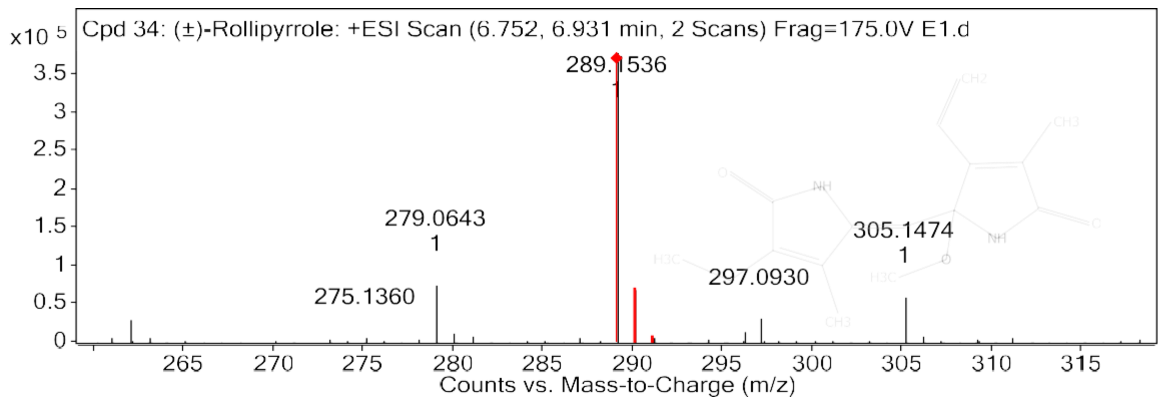

MS Spectrum Peak List

m/z	Calc m/z	Diff(ppm)	z	Abund	Formula	Ion
164.0189				43488.3		
279.0643			1	76725		
289.1536	289.1547	3.84	1	380285.22	C16 H20 N2 O3	(M+H)+
290.1567	290.1578	3.69	1	71316.08	C16 H20 N2 O3	(M+H)+
291.1585	291.1604	6.57	1	8650.25	C16 H20 N2 O3	(M+H)+
305.1474			1	61204.59		
323.1377			1	141345.27		
344.1448			1	205775.28		
345.1477			1	38102.88		
679.5096			1	56228.59		

MSMS Spectrum

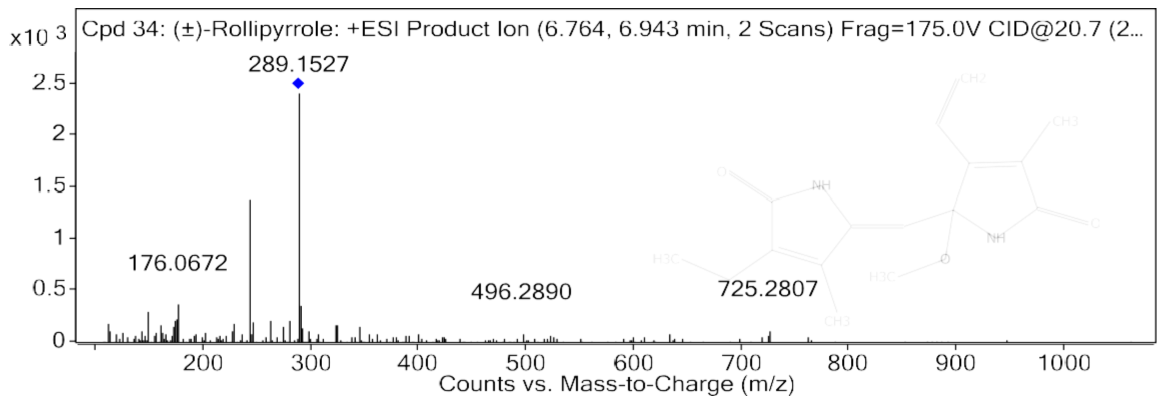

MS/MS Spectrum Peak List

m/z	z	Abund
148.0745		297.49
174.0526		211.85
175.0853		235.96
176.0672		368.08
243.1479	1	1381.61
245.0875	1	199.73
262.1033		218.4
279.0594		222.05
289.1527	1	2409.6
290.157	1	356.01

Compound Structure

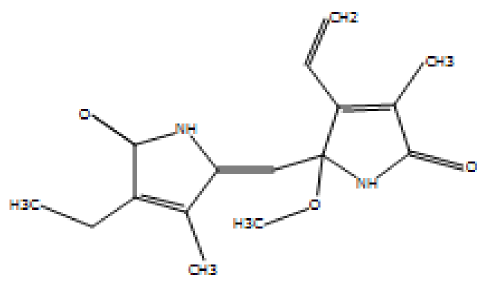

11.N6-cis-p- Coumaroylserotonin

Compound Label	Name	*m/z*	RT	Algorithm	Mass
Cpd 36: N6-cis-p-Coumaroylserotonin		323.1379	7.024	Auto MS/MS	322.1306

MS Spectrum

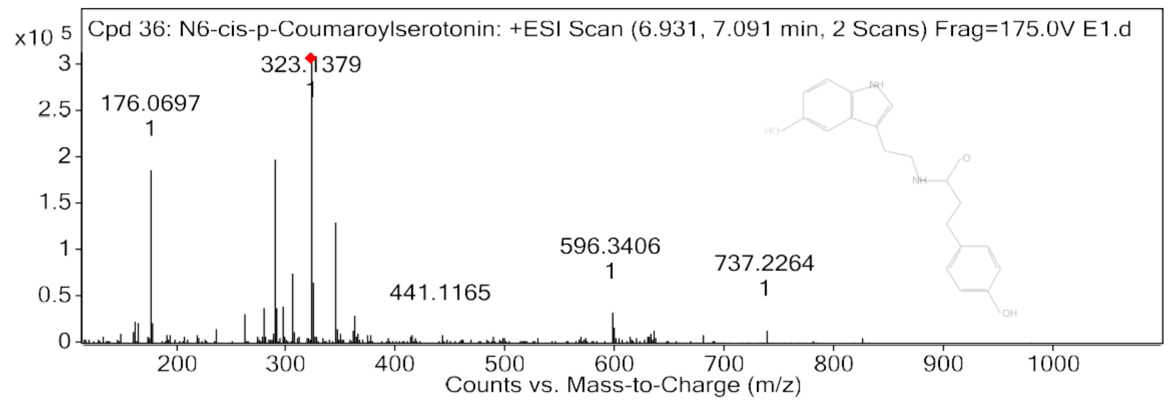

MS Zoomed Spectrum

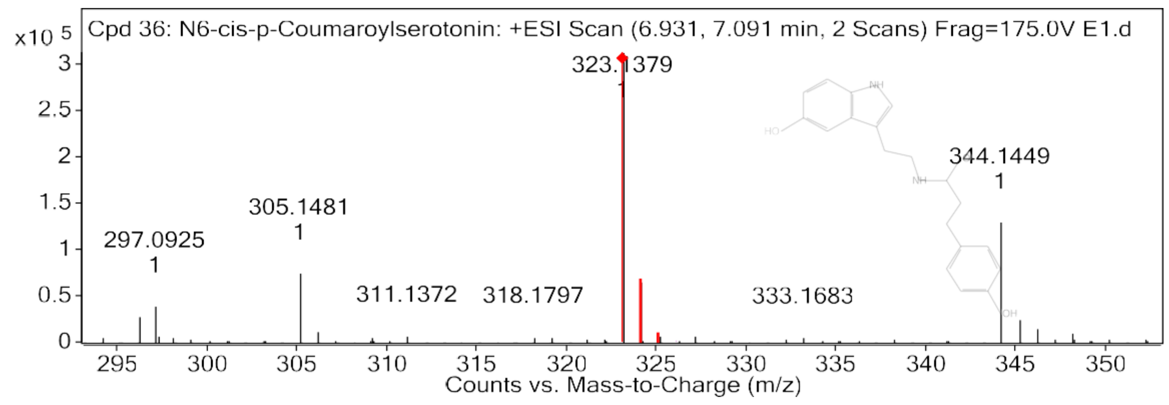

MS Spectrum Peak List

m/z	Calc m/z	Diff(ppm)	z	Abund	Formula	Ion
176.0697			1	187442.75		
279.0644			1	38416.14		
289.1536			1	198736.88		
290.1566			1	38135.68		
297.0925			1	39906.24		
305.1481			1	75507.7		
323.1379	323.139	3.57	1	313297.84	C19 H18 N2 O3	(M+H)+
324.1408	324.1422	4.39	1	66862.52	C19 H18 N2 O3	(M+H)+
325.1439	325.1449	3.08	1	8663.55	C19 H18 N2 O3	(M+H)+
344.1449			1	130385.67		

MSMS Spectrum

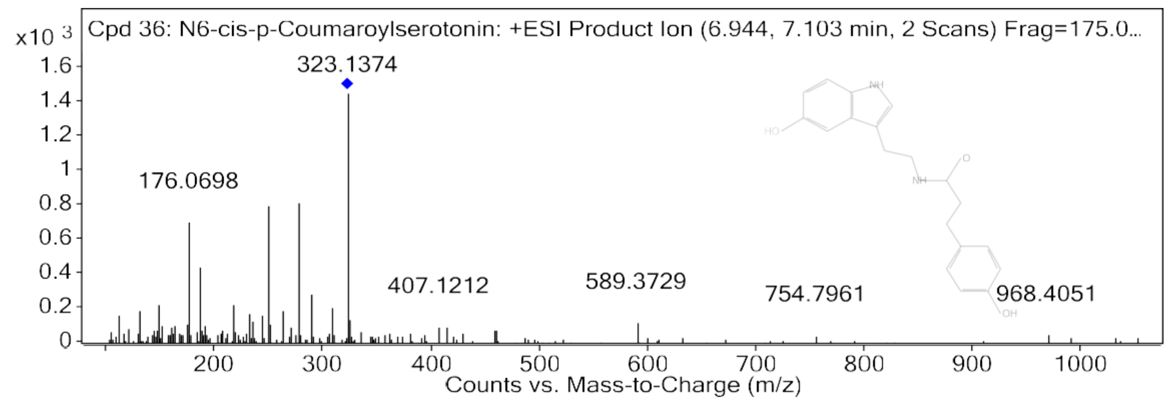

MS/MS Spectrum Peak List

m/z	z	Abund
130.0639		194.98
148.074		222.42
176.0698		703.97
186.0792	1	444.4
217.0598	1	222.92
250.1206	1	800.58
277.1309	1	814.05
289.1553	1	290.36
308.1156		209.24
323.1374	1	1451.81

Compound Structure

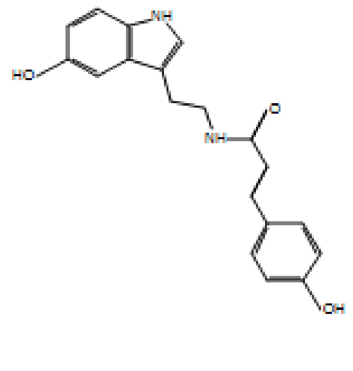

12. 1-Methoxy-1H-indole- 3-carboxaldehyde

Compound Label	Name	*m/z*	RT	Algorithm	Mass
Cpd 37: 1-Methoxy-1H-indole-3-carboxaldehyde	**1-Methoxy-1H-indole-3-carboxaldehyde**	176.0697	7.09	Auto MS/MS	175.0625

MS Spectrum

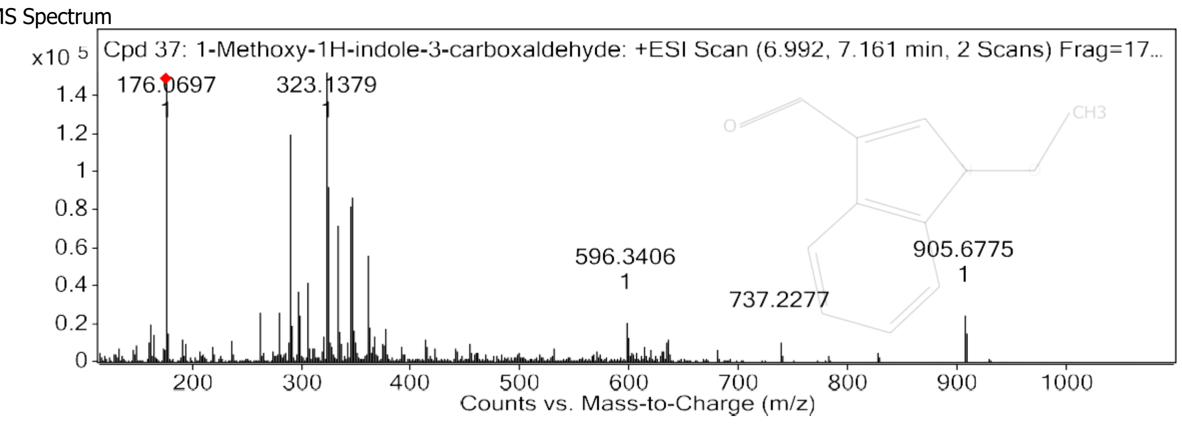

MS Zoomed Spectrum

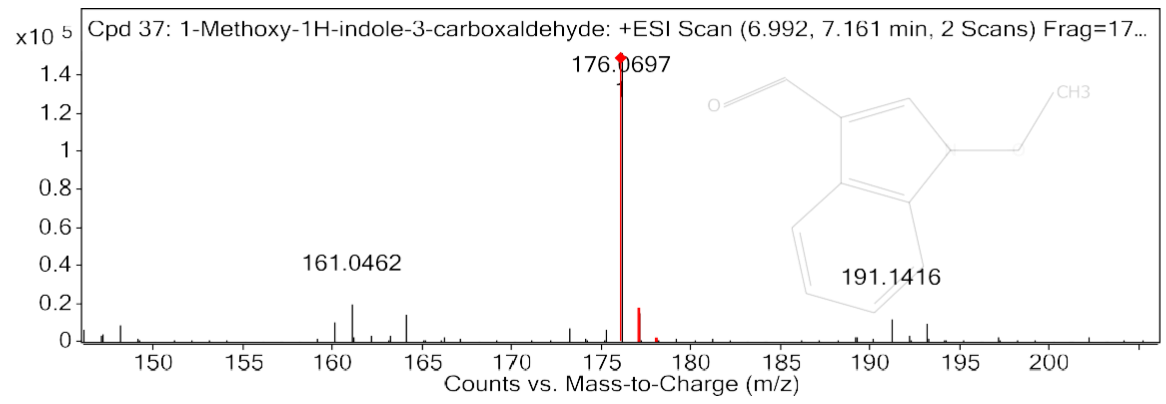

MS Spectrum Peak List

m/z	Calc m/z	Diff(ppm)	z	Abund	Formula	Ion
176.0697	176.0706	4.87	1	152657.19	C10 H9 N O2	(M+H)+
177.0728	177.0738	5.73	1	15654.78	C10 H9 N O2	(M+H)+
178.0773	178.0761	-6.98	1	1771.43	C10 H9 N O2	(M+H)+
289.1535			1	120112.23		
323.1379			1	448656.88		
324.1411			1	92780.34		
333.1651			1	72210.33		
344.145			1	82523.17		
346.1603			1	87347.2		
360.2155			1	56769.48		

MSMS Spectrum

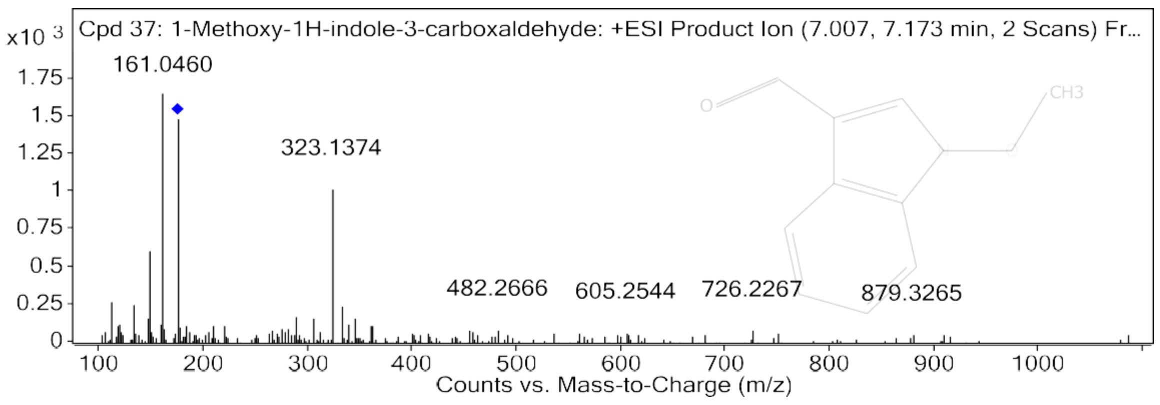

MS/MS Spectrum Peak List

m/z	z	Abund
111.0429		279.72
133.052	1	261.78
146.0623		173.42
148.0743	1	617.08
160.0384		474.19
161.046	1	1658.19
176.0691	1	1490.61
289.1558		180.66
323.1374	1	1024.93
333.1673		249.48

Compound Structure

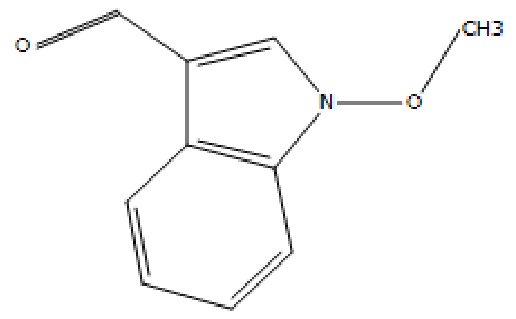

13.(10Z,14E,16E)- 10,14,16-Octadecatrien-12- ynoic acid

Compound Label	Name	*m/z*	RT	Algorithm	Mass
Cpd 41: (10Z,14E,16E)-10,14,16-Octadecatrien-12-ynoic acid	**(10Z,14E,16E)-10,14,16-Octadecatrien-12-ynoic acid**	275.1991	8.672	Auto MS/MS	274.192

MS Spectrum

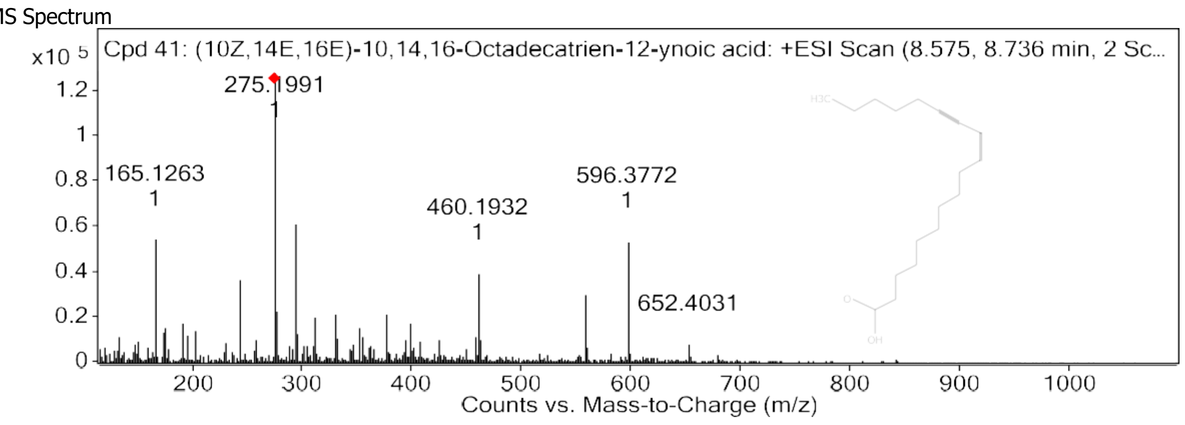

MS Zoomed Spectrum

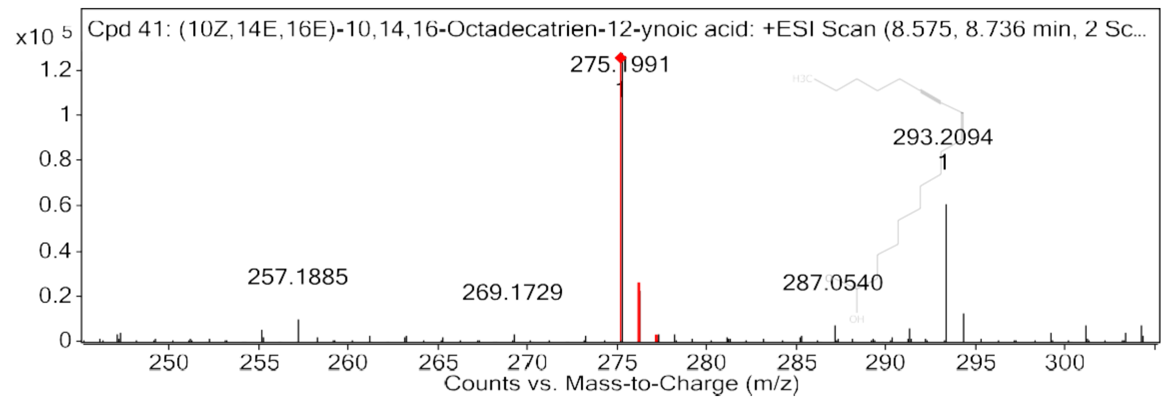

MS Spectrum Peak List

m/z	Calc m/z	Diff(ppm)	z	Abund	Formula	Ion
165.1263			1	54683.75		
242.2828			1	37290.71		
275.1991	275.2006	5.32	1	128396.67	C18 H26 O2	(M+H)+
276.2027	276.204	4.51	1	23482.82	C18 H26 O2	(M+H)+
277.2097	277.2069	-10.14	1	3975.47	C18 H26 O2	(M+H)+
293.2094			1	61671.11		
376.1166			1	22008.62		
460.1932			1	39823.35		
557.1457			1	30466.36		
596.3772			1	53871.45		

MSMS Spectrum

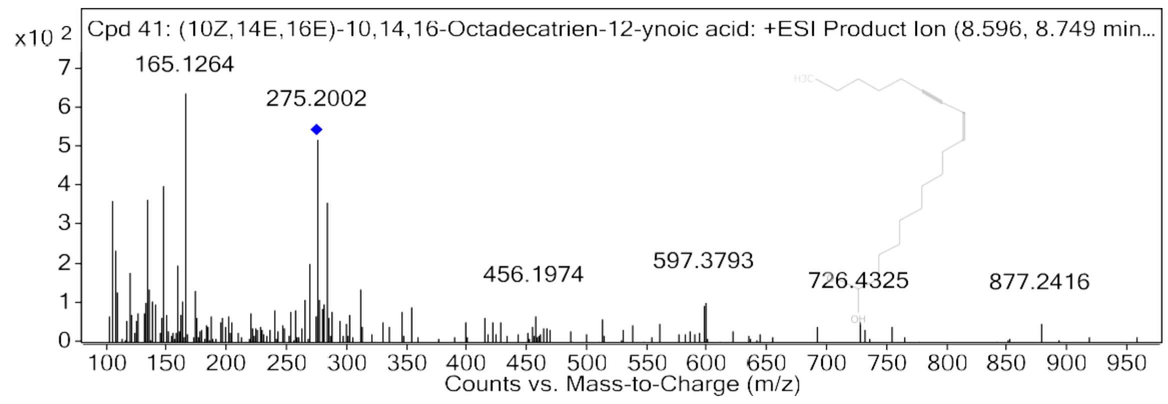

MS/MS Spectrum Peak List

m/z	z	Abund
105.0697		362.65
107.0846		236.65
119.0838		179.48
133.1001		366.1
147.1165		402.09
159.1155		198.02
165.1264		638.17
268.0705		203.78
275.2002	1	518.97
283.094	1	359.94

Compound Structure

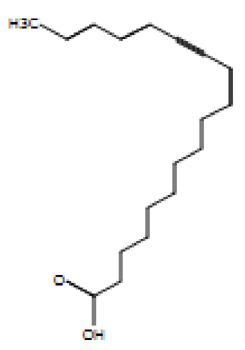

14.3,4',5,6,8- Pentamethoxyflavone

Compound Label	Name	*m/z*	RT	Algorithm	Mass
Cpd 47: 3,4',5,6,8-Pentamethoxyflavone	**3,4',5,6,8-Pentamethoxyflavone**	373.1264	11.085	Auto MS/MS	372.1191

MS Spectrum

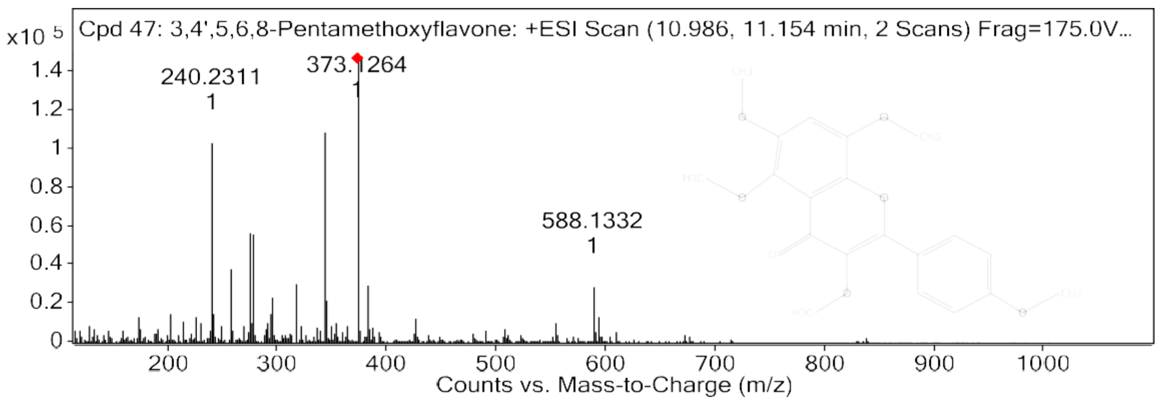

MS Zoomed Spectrum

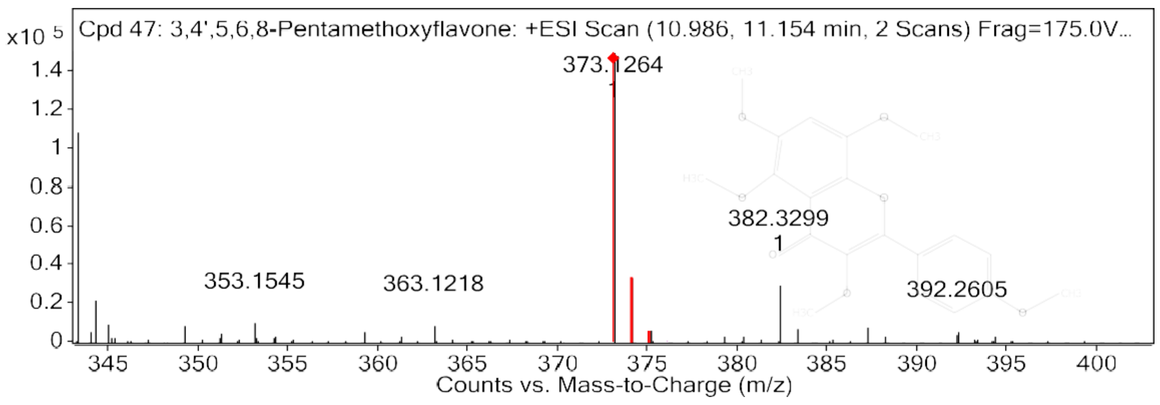

MS Spectrum Peak List

m/z	Calc m/z	Diff(ppm)	z	Abund	Formula	Ion
240.2311			1	103634.71		
257.1887			1	38998.97		
275.1988			1	57326.45		
277.2144			1	56825.9		
316.2828			1	30835.76		
343.0076			1	39962.27		
343.2939			1	108983.25		
373.1264	373.1282	4.86	1	150299.31	C20 H20 O7	(M+H)+
374.1296	374.1316	5.36	1	34222.18	C20 H20 O7	(M+H)+
375.1334	375.134	1.54	1	7030.86	C20 H20 O7	(M+H)+

MSMS Spectrum

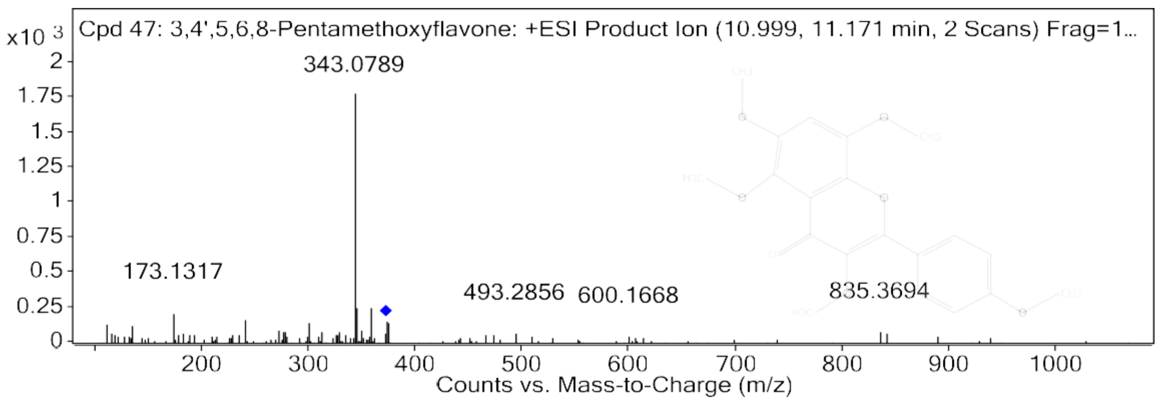

MS/MS Spectrum Peak List

m/z	z	Abund
111.0802		144.01
135.1156		130.71
173.1317		218.86
240.2287		174.64
300.0633		152.52
343.0789	1	1786.33
344.0843	1	261.44
358.101		254.49
373.1273		163.04
374.1289		144.27

Compound Structure

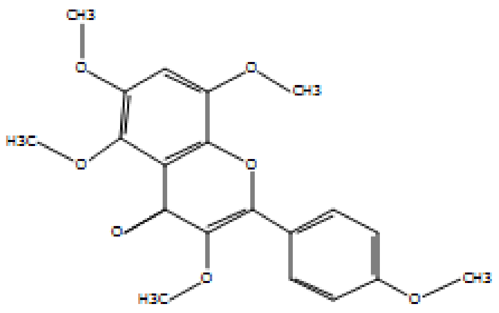

15.Terminaline

Compound Label	Name	*m/z*	RT	Algorithm	Mass
Cpd 48: Terminaline	**Terminaline**	364.3195	12.545	Auto MS/MS	363.3122

MS Spectrum

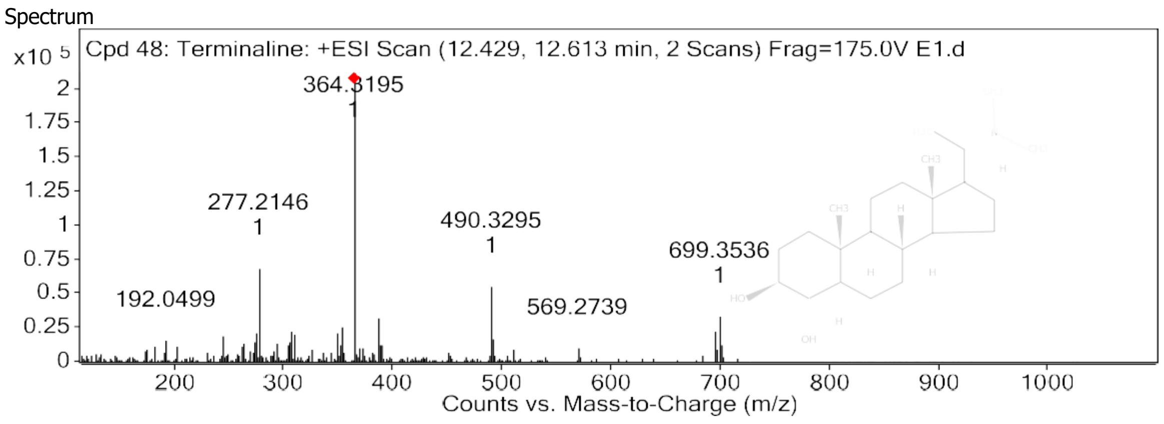

MS Zoomed Spectrum

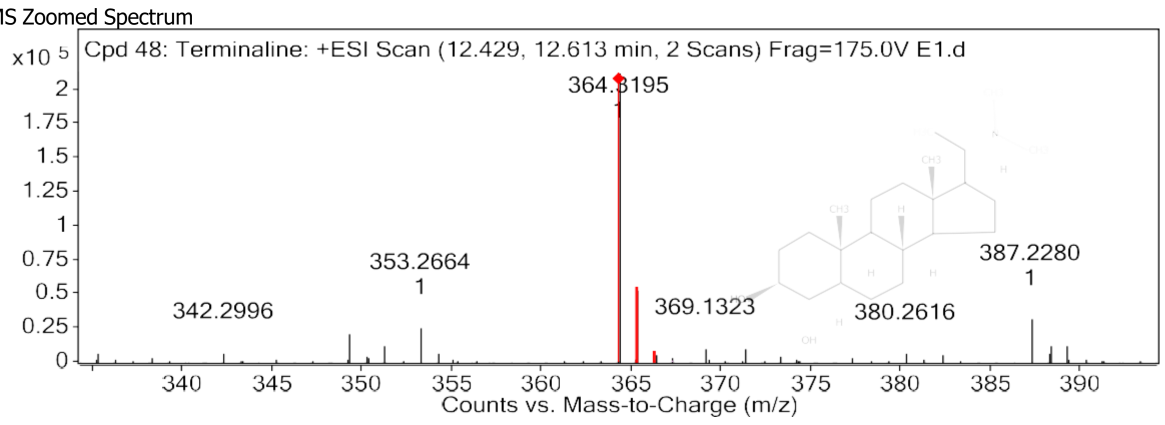

MS Spectrum Peak List

m/z	Calc m/z	Diff(ppm)	z	Abund	Formula	Ion
277.2146			1	69223.59		
307.0951				23004.31		
353.2664			1	25965.64		
364.3195	364.321	4.2	1	212781.45	C23 H41 N O2	(M+H)+
365.3228	365.3243	4.07	1	53703.81	C23 H41 N O2	(M+H)+
366.3256	366.3274	4.82	1	6519.13	C23 H41 N O2	(M+H)+
387.228			1	32502.81		
490.3295			1	55913.27		
694.3981			1	22894.61		
699.3536			1	33799.69		

MSMS Spectrum

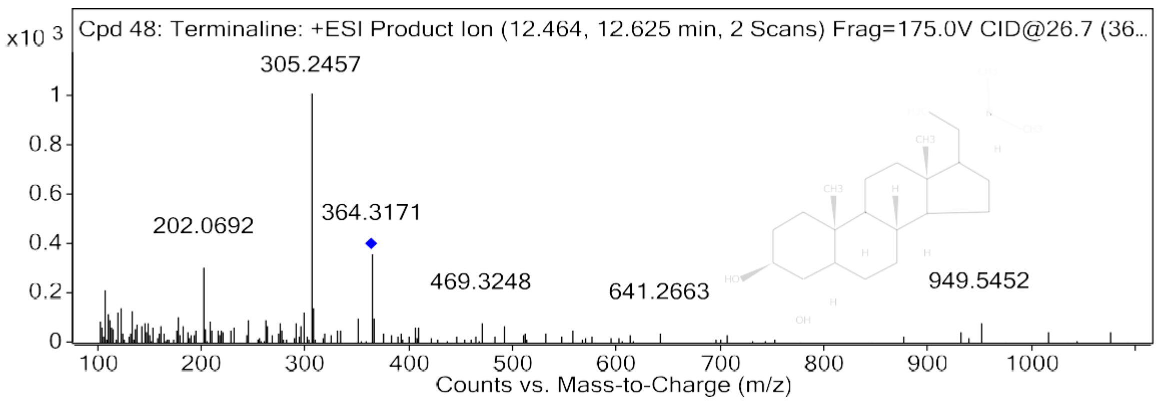

MS/MS Spectrum Peak List

m/z	z	Abund
107.0851		217.03
109.0626		121.84
119.0843		128.35
121.0946		143
133.1011		135.12
202.0692		309.36
298.2733		128.07
305.2457	1	1014.99
306.2541	1	146.23
364.3171	1	363.77

Compound Structure

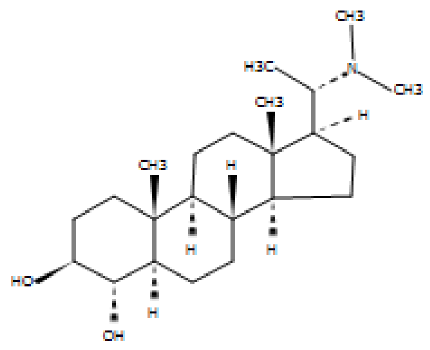

16.Palmitic amide

Compound Label	Name	*m/z*	RT	Algorithm	Mass
Cpd 69: Palmitic amide	**Palmitic amide**	256.2627	16.877	Auto MS/MS	255.2554

MS Spectrum

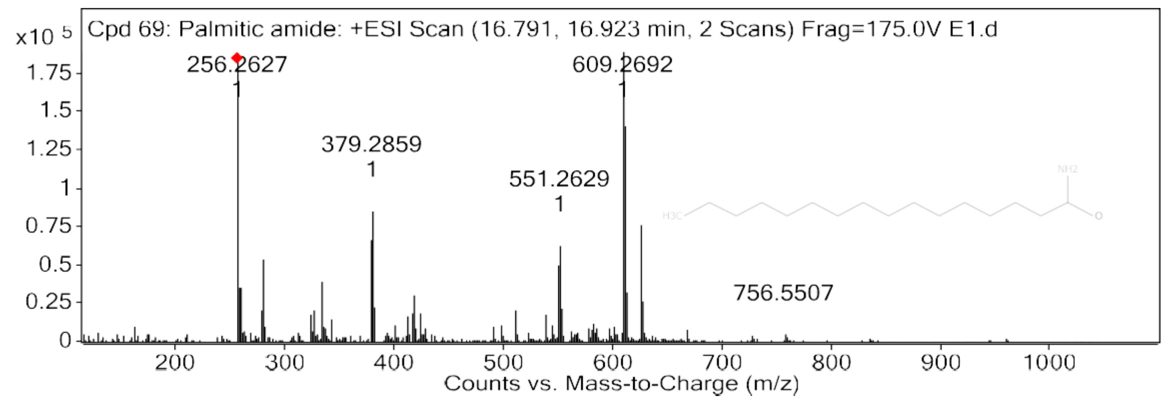

MS Zoomed Spectrum

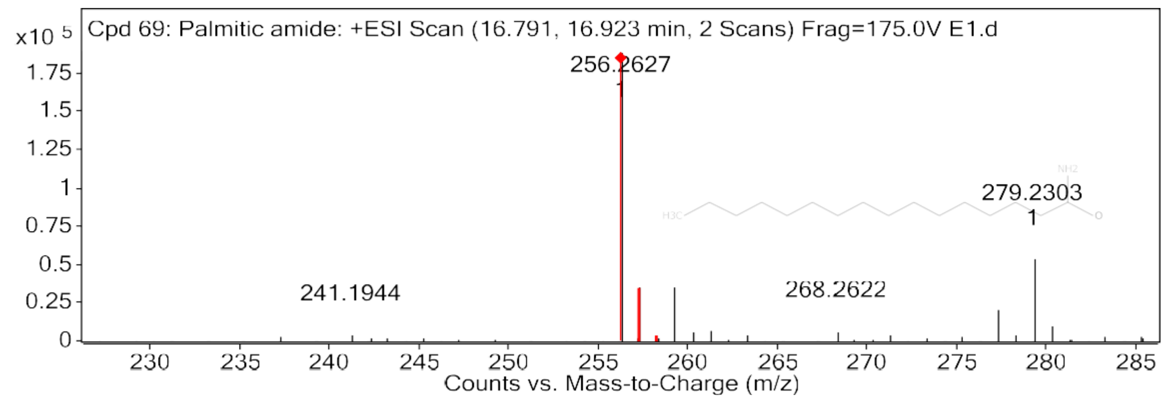

MS Spectrum Peak List

m/z	Calc m/z	Diff(ppm)	z	Abund	Formula	Ion
256.2627	256.2635	3.15	1	189479.02	C16 H33 N O	(M+H)+
257.266	257.2668	3.03	1	35867.96	C16 H33 N O	(M+H)+
258.2691	258.2698	2.75	1	3374.17	C16 H33 N O	(M+H)+
279.2303			1	54571.22		
378.2984				67069.73		
379.2859			1	86364.88		
551.2629			1	63690.75		
609.2692			1	372537.75		
610.2721			1	141414.7		
625.2639			1	77114.96		

MSMS Spectrum

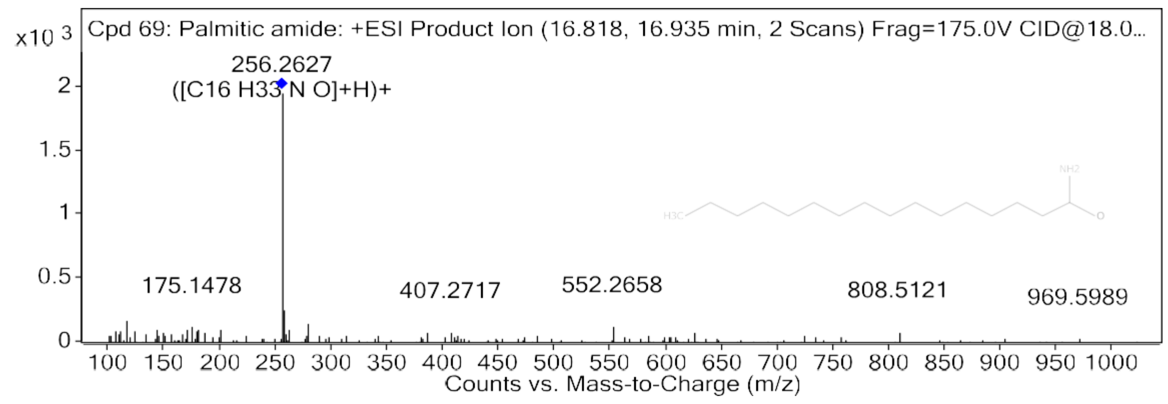

MS/MS Spectrum Peak List

m/z	Calc m/z	Diff (ppm)	z	Abund	Formula	Ion
117.0695				180.95		
143.0816				101.38		
175.1478				123.04		
181.1167				104.46		
201.1615				103.02		
256.2627	256.2635	3.19	1	1953.54	C16 H33 N O	(M+H)+
257.2666			1	261.55		
261.2145				104.45		
279.2341				151.07		
552.2658				128.32		

Compound Structure

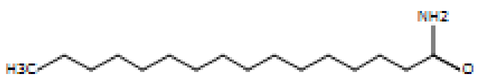

17.Oleamide

Compound Label	Name	*m/z*	RT	Algorithm	Mass
Cpd 71: Oleamide	**Oleamide**	282.2778	17.242	Auto MS/MS	281.2705

MS Spectrum

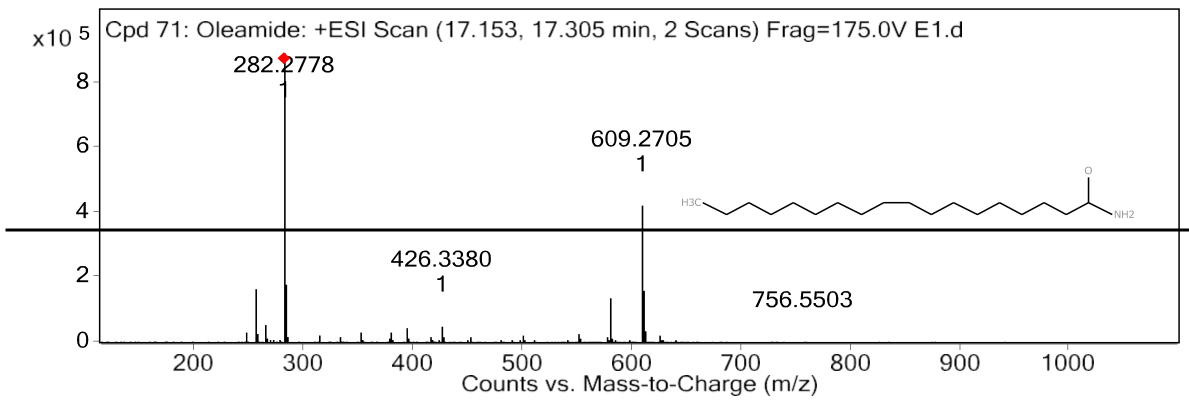

MS Zoomed Spectrum

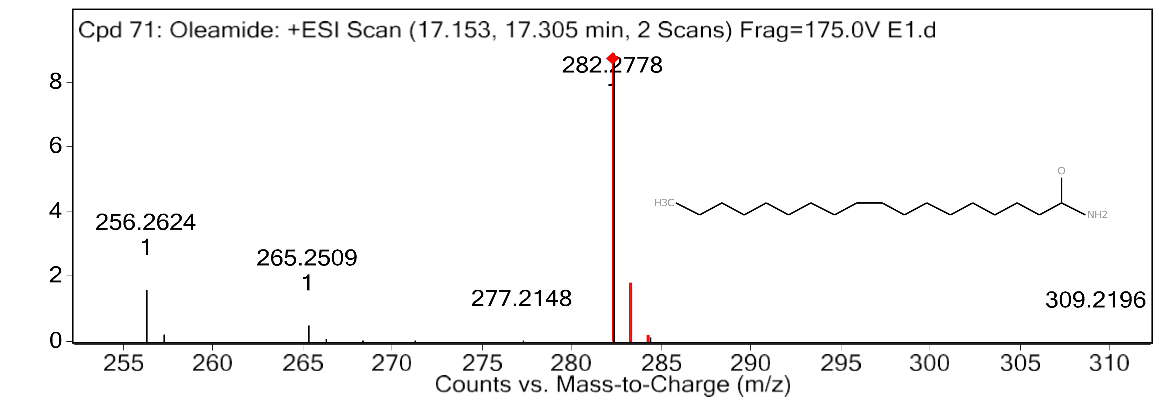

MS Spectrum Peak List

m/z	Calc m/z	Diff(ppm)	z	Abund	Formula	Ion
256.2624			1	167086.52		
265.2509			1	57173.25		
282.2778	282.2791	4.84	1	889493.5	C18 H35 N O	(M+H)+
283.2813	283.2824	3.96	1	177573.92	C18 H35 N O	(M+H)+
284.2847	284.2855	2.8	1	18589.79	C18 H35 N O	(M+H)+
426.338			1	52614.42		
579.2945			1	137876.38		
580.2973			1	52851.59		
609.2705			1	422861.88		
610.2732			1	162752.48		

MSMS Spectrum

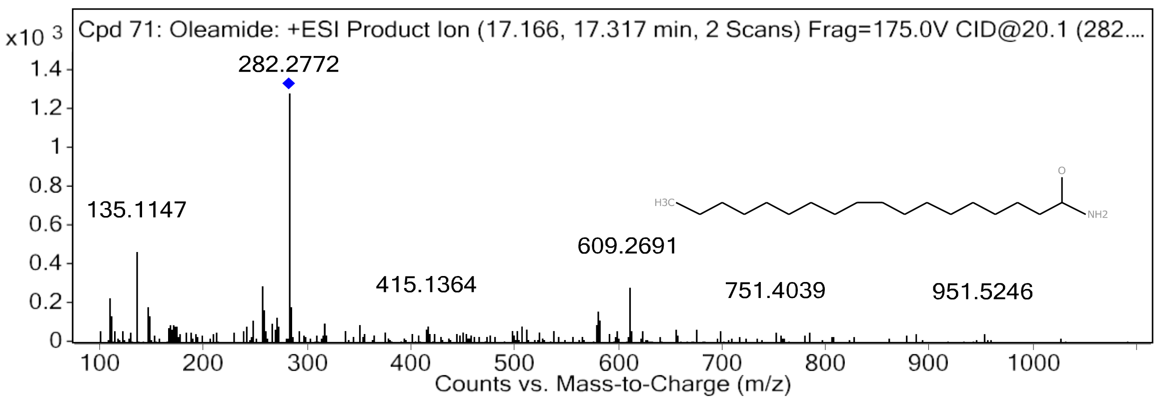

MS/MS Spectrum Peak List

m/z	z	Abund
109.1005		233.63
135.1147		470.83
145.1005		187.43
256.2627		293.62
257.2685		170.13
282.2772	1	1284.93
283.2797	1	182.72
579.2953	1	164.03
609.2691		284.43
610.2661		214.43

Compound Structure

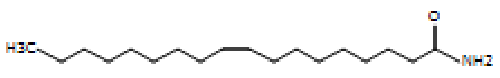

18.Pheophorbide a

Compound Label	Name	*m/z*	RT	Algorithm	Mass
Cpd 73: Pheophorbide a	**Pheophorbide a**	593.2738	17.474	Auto MS/MS	592.2665

MS Spectrum

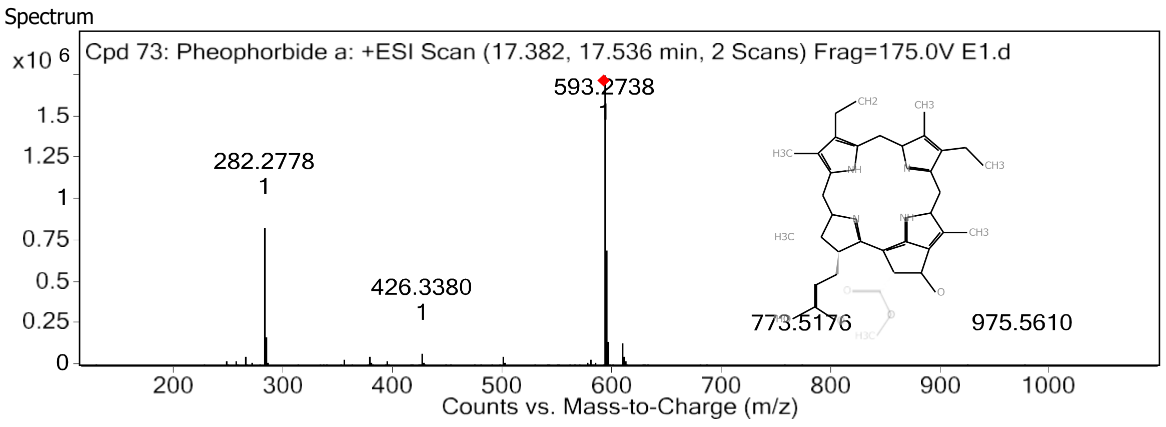

MS Zoomed Spectrum

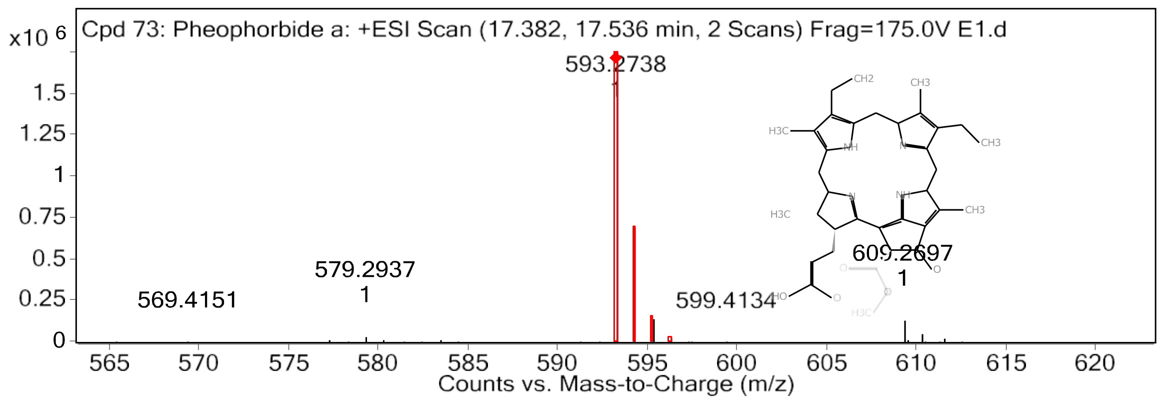

MS Spectrum Peak List

m/z	Calc m/z	Diff(ppm)	z	Abund	Formula	Ion
282.2778			1	830542.13		
283.2812			1	173045.11		
378.3348			1	55495.14		
426.338			1	69321.65		
500.4441			1	57623.04		
593.2738	593.2758	3.49	1	1750006.63	C35 H36 N4 O5	(M+H)+
594.2768	594.279	3.65	1	692155	C35 H36 N4 O5	(M+H)+
595.2802	595.2819	2.86	1	140096.31	C35 H36 N4 O5	(M+H)+
596.284	596.2847	1.19	1	24364.79	C35 H36 N4 O5	(M+H)+
609.2697			1	138188.14		

MSMS Spectrum

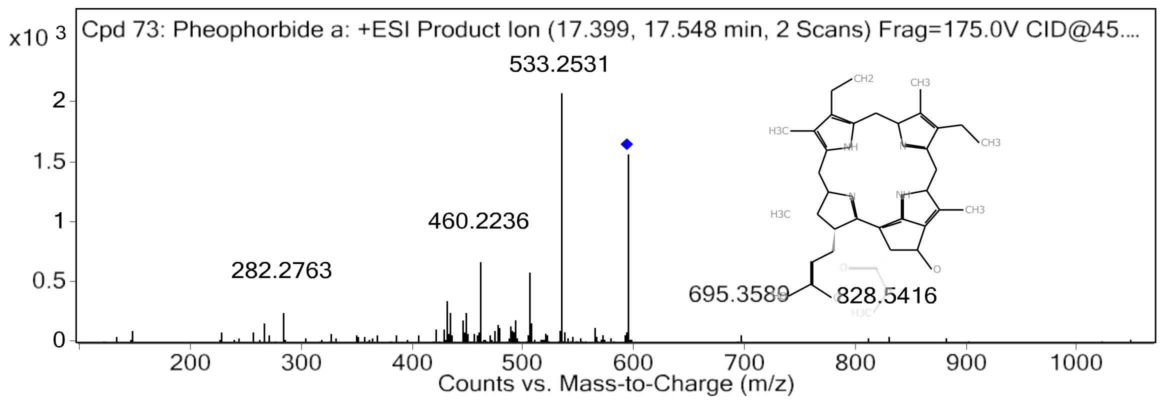

MS/MS Spectrum Peak List

m/z	z	Abund
282.2763		254.82
431.1817	1	348.57
433.2377	1	244.44
447.2127		249.51
460.2236		667.9
461.2307		434.85
505.2187		588.91
533.2531	1	2081.81
593.2724	1	1570.28
594.2767	1	193.14

Compound Structure

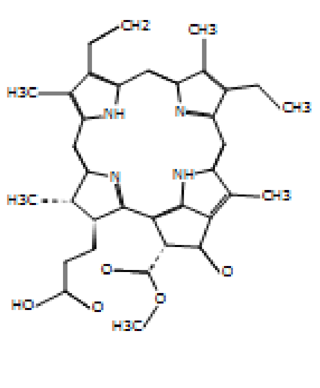

19.Pyropheophorbide a

Compound Label	Name	*m/z*	RT	Algorithm	Mass
Cpd 76: Pyropheophorbide a	**Pyropheophorbide a**	535.2688	17.918	Auto MS/MS	534.2615

MS Spectrum

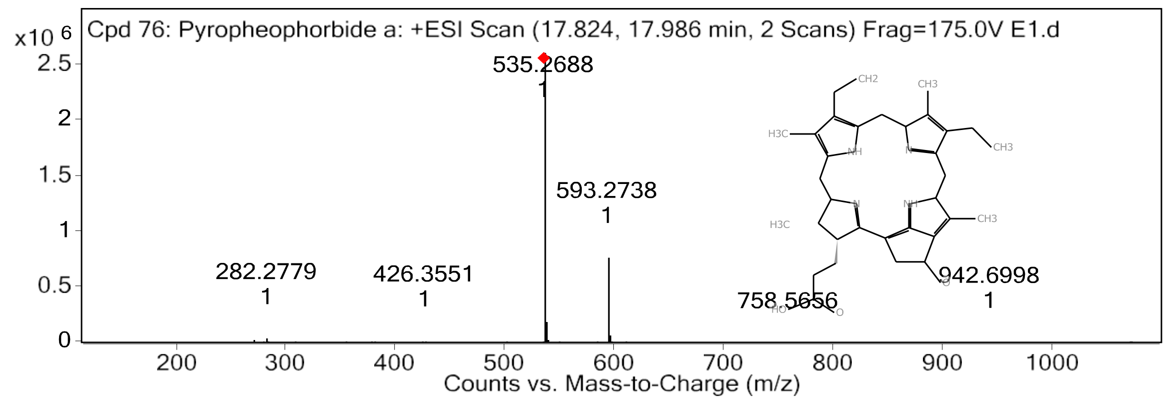

MS Zoomed Spectrum

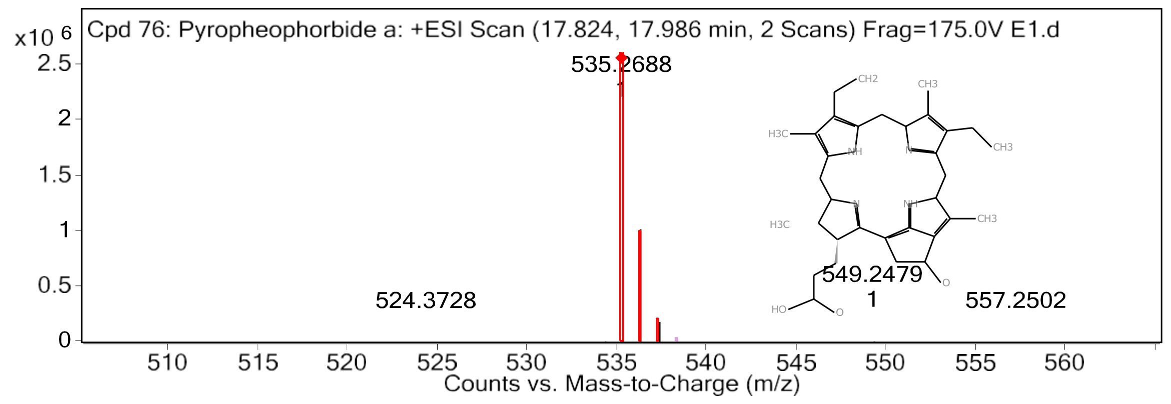

MS Spectrum Peak List

m/z	Calc m/z	Diff(ppm)	z	Abund	Formula	Ion
270.2776			1	22432.38		
282.2779			1	39346.35		
500.4437			1	18362.78		
535.2688	535.2704	2.88	1	2610777.5	C33 H34 N4 O3	(M+H)+
536.2717	536.2735	3.44	1	1026844.75	C33 H34 N4 O3	(M+H)+
537.2751	537.2765	2.59	1	188548.89	C33 H34 N4 O3	(M+H)+
538.2777			1	25045.81		
593.2738			1	773113.69		
594.2775			1	300161.22		
595.2804			1	62518.8		

MSMS Spectrum

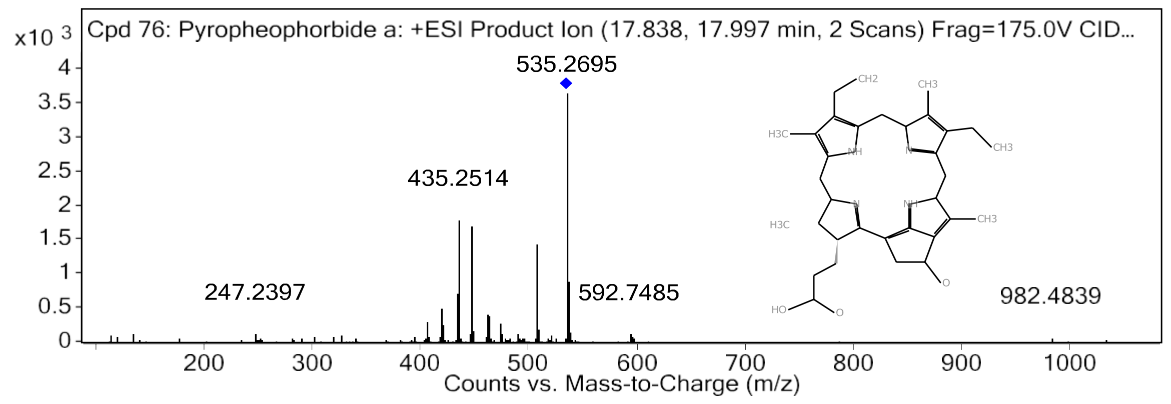

MS/MS Spectrum Peak List

m/z	z	Abund
419.2234	1	511.17
433.1992	1	712.74
433.2335		425.71
434.2451		899.33
435.2514	1	1802.99
447.216	1	1709.91
461.2314	1	423.19
507.2728	1	1454.36
535.2695	1	3658.77
536.2718	1	905.38

Compound Structure

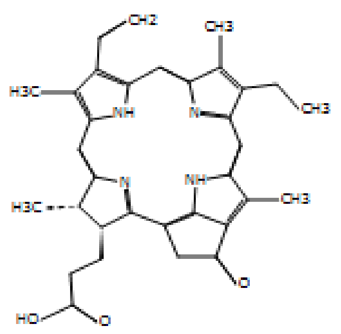

Negative Analysis

20.Glucoraphanin

Compound Label	Name	*m/z*	RT	Algorithm	Mass
Cpd 2: Glucoraphanin	**Glucoraphanin**	436.0367	1.124	Auto MS/MS	437.0439

MS Spectrum

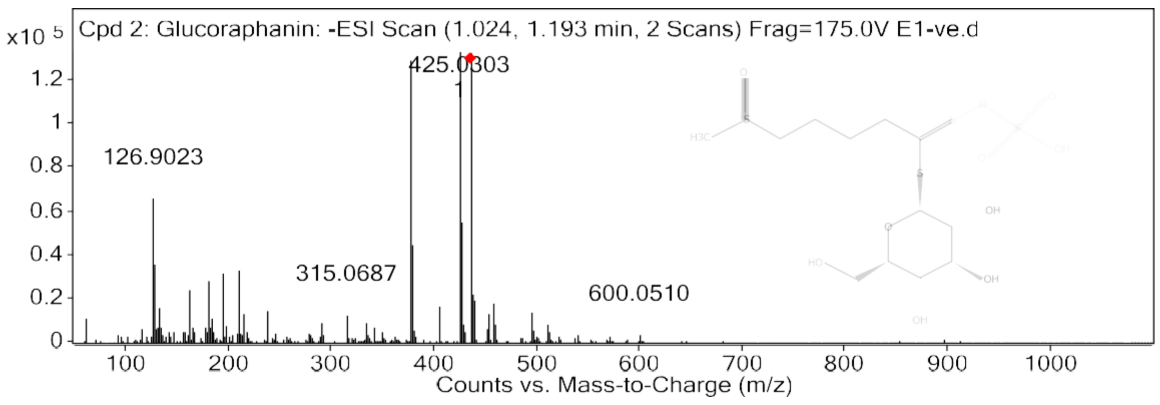

MS Zoomed Spectrum

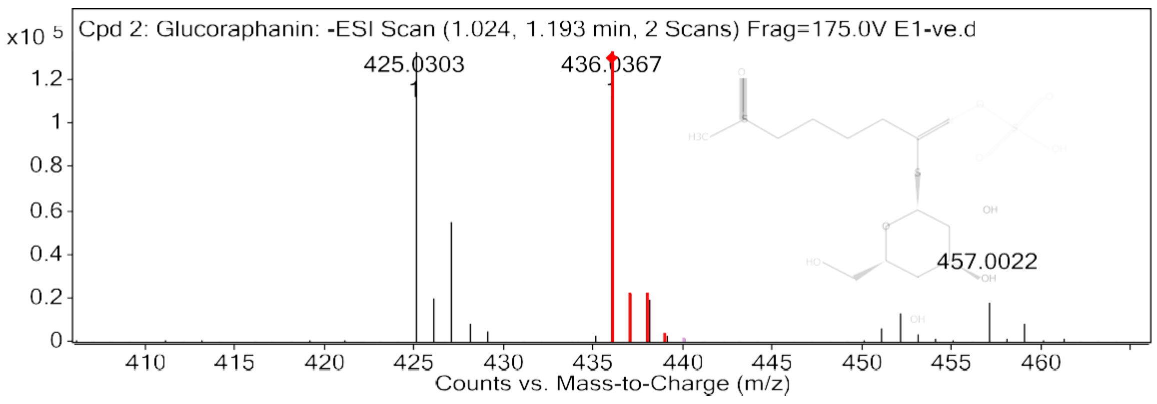

MS Spectrum Peak List

m/z	Calc m/z	Diff(ppm)	z	Abund	Formula	Ion
126.9023				66650.58		
128.0328				36052.26		
377.0812			1	129898.73		
379.0791			1	45399.72		
425.0303			1	134423.22		
427.0273			1	55857.03		
436.0367	436.0411	10.18	1	133187.59	C12 H23 N O10 S3	(M-H)-
437.0388	437.0438	11.51	1	22653.03	C12 H23 N O10 S3	(M-H)-
438.0339	438.0387	10.84	1	20003.11	C12 H23 N O10 S3	(M-H)-
439.0378	439.0411	7.67	1	3646.8	C12 H23 N O10 S3	(M-H)-

MSMS Spectrum

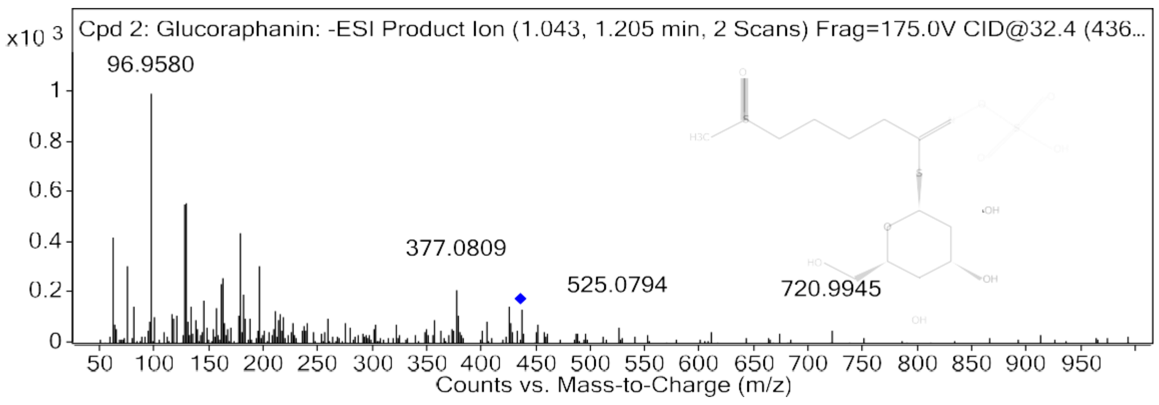

MS/MS Spectrum Peak List

m/z	z	Abund
61.9856		420.4
74.9894		311.93
95.9485		459.41
96.958		995.19
126.9015		555.99
128.032		557.71
161.0436		235.41
162.0201	1	264.12
178.0128	1	442.75
195.0461		311.4

Compound Structure

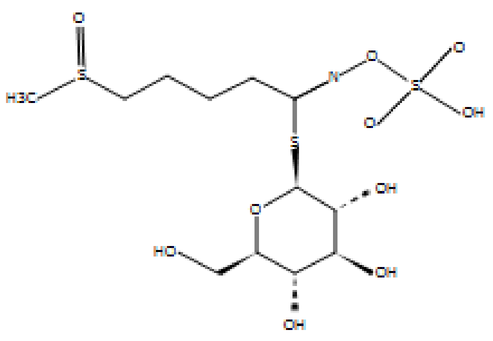

21.(S)-2-(Hydroxymethyl)glutar ate

Compound Label	Name	m/z	RT	Algorithm	Mass
Cpd 3: (S)-2-(Hydroxymethyl)glutarate	**(S)-2-(Hydroxymethyl)glutarate**	161.0429	1.202	Auto MS/MS	162.0501

MS Spectrum

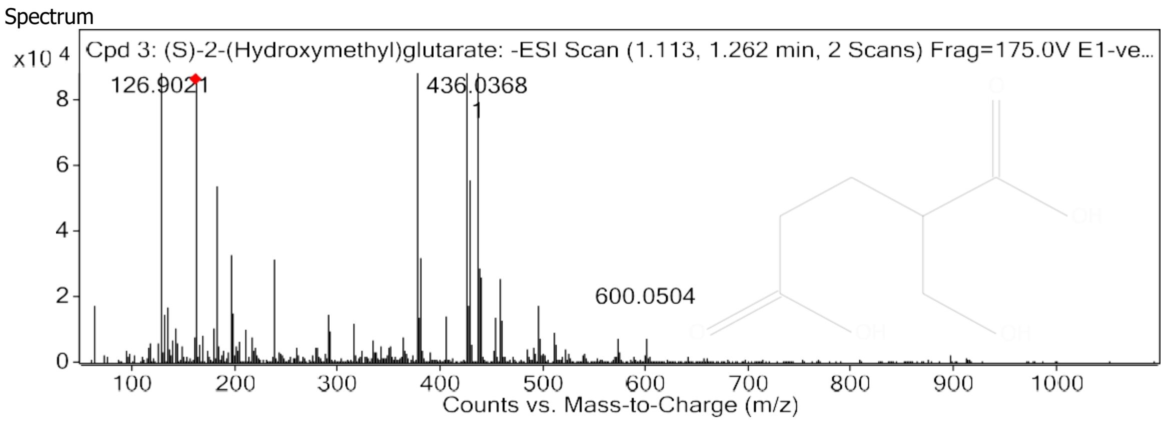

MS Zoomed Spectrum

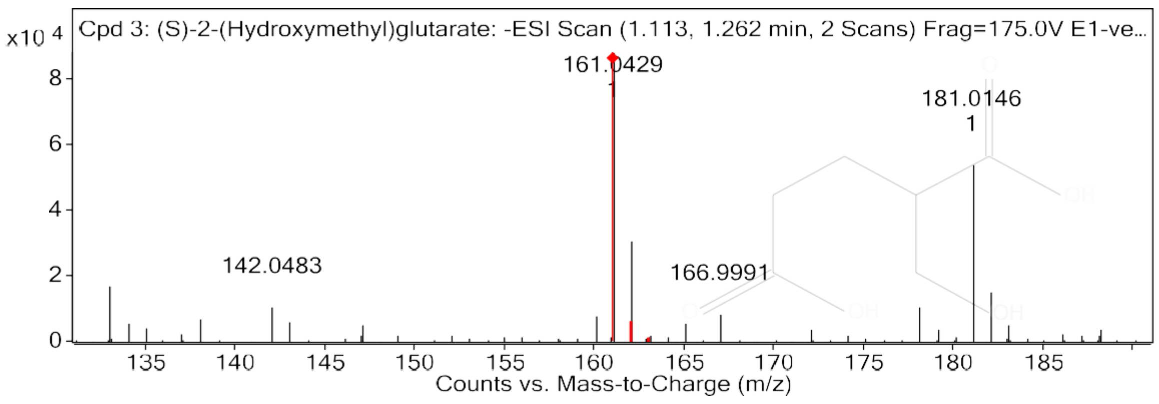

MS Spectrum Peak List

m/z	Calc m/z	Diff(ppm)	z	Abund	Formula	Ion
126.9021				99789.22		
128.0328			1	59425.54		
161.0429	161.0455	16.2	1	88201.73	C6 H10 O5	(M-H)-
162.0449	162.049	24.98	1	7235.87	C6 H10 O5	(M-H)-
163.0461	163.0502	25.05	1	2053.09	C6 H10 O5	(M-H)-
181.0146			1	54181.4		
377.0814			1	93757.94		
425.0301			1	125552.94		
427.0272			1	55959.16		
436.0368			1	179343.88		

MSMS Spectrum

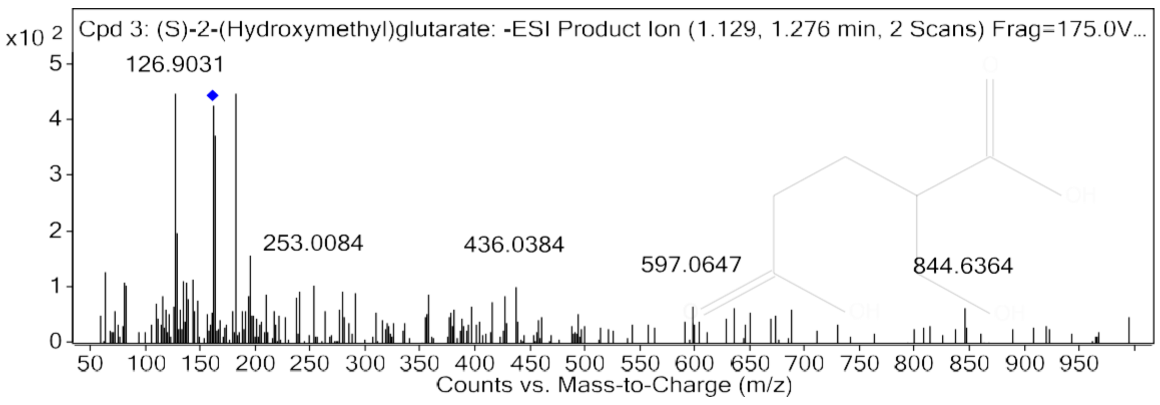

MS/MS Spectrum Peak List

m/z	z	Abund
61.9864		127.81
126.9031		449.42
128.0326		198.91
134.0441		112.95
142.0449		115.21
161.0423		426.26
162.0216		372.88
162.0431		142
181.0157	1	448.17
195.0462		158.81

Compound Structure

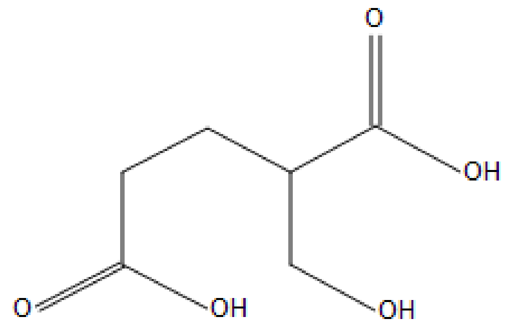

22. 2-Deoxy-scyllo- inosose

Compound Label	Name	m/z	RT	Algorithm	Mass
Cpd 5: 2-Deoxy-scyllo-inosose	**2-Deoxy-scyllo-inosose**	161.0431	1.485	Auto MS/MS	162.0502

MS Spectrum

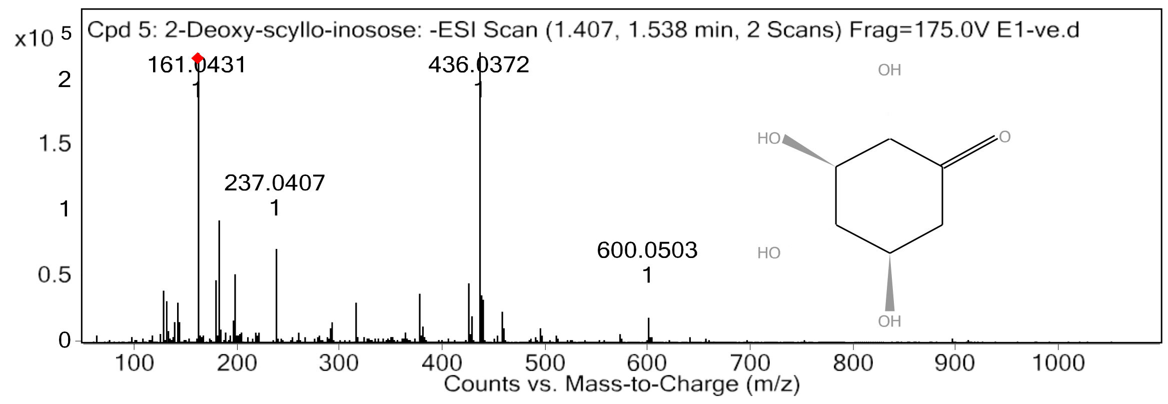

MS Zoomed Spectrum

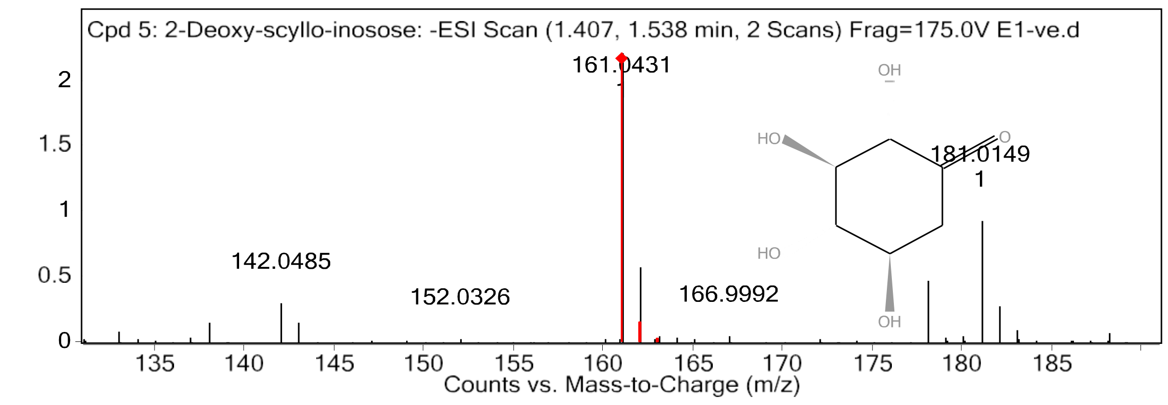

MS Spectrum Peak List

m/z	Calc m/z	Diff(ppm)	z	Abund	Formula	Ion
161.0431	161.0455	15.32	1	221169.16	C6 H10 O5	(M-H)-
162.0206			1	58622.91		
162.0455	162.049	21.26	1	15831.35	C6 H10 O5	(M-H)-
163.0449	163.0502	32.86	1	5208.62	C6 H10 O5	(M-H)-
**m/z**	**Calc m/z**	**Diff(ppm)**	**z**	**Abund**	**For**	**Ion**
177.9974			1	48121.68		
181.0149			1	93657.05		
196.992				52145.2		
237.0407			1	71376.71		
425.0307			1	45480.8		
436.0372			1	226115.59		

MSMS Spectrum

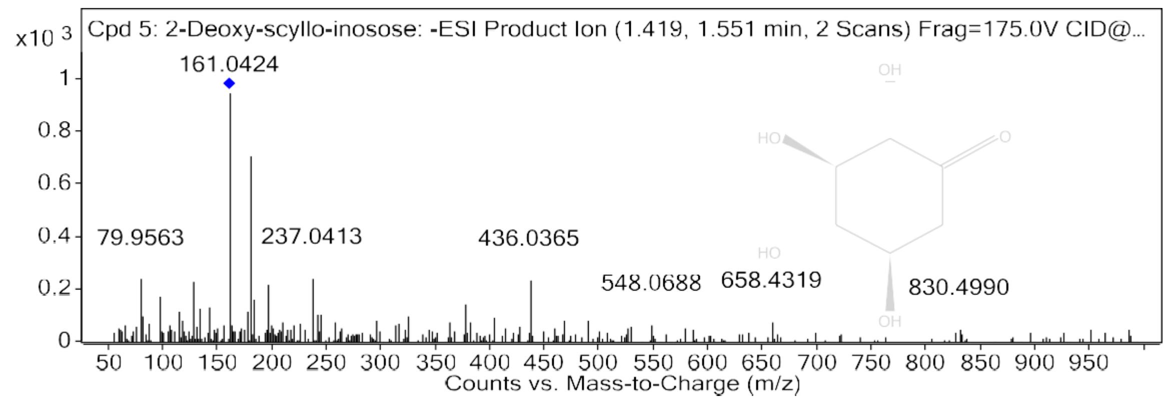

MS/MS Spectrum Peak List

m/z	z	Abund
79.9563		242.11
96.9582		175.65
128.031		229.99
161.0424	1	948.92
162.0189		480.26
181.0151		708.62
183.0251		165.56
196.9927		218.9
237.0413		246.77
436.0365	1	239.92

Compound Structure

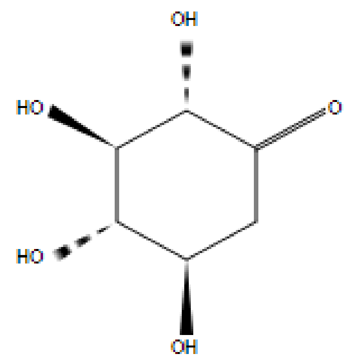

23.Artomunoxanthentrion e epoxide

Compound Label	Name	m/z	RT	Algorithm	Mass
Cpd 11: Artomunoxanthentrione epoxide	**Artomunoxanthentrion e epoxide**	461.1265	3.084	Auto MS/MS	462.1337

MS Spectrum

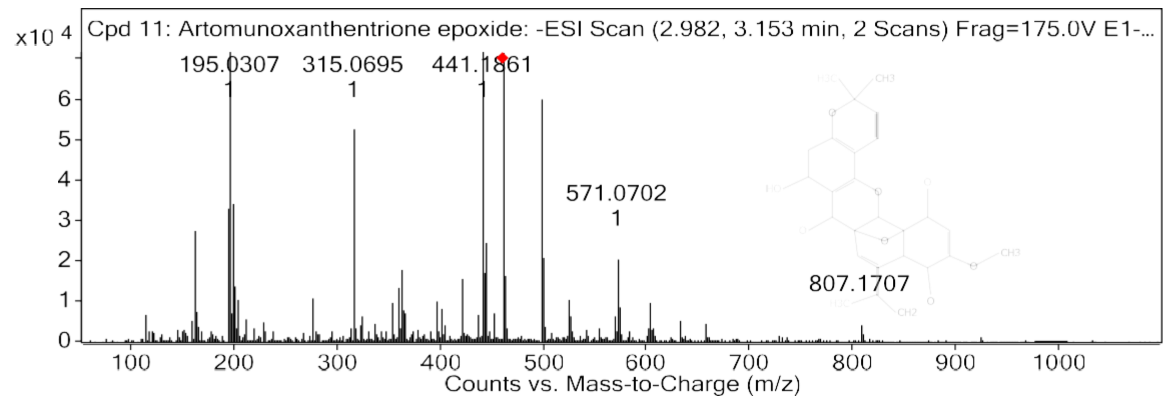

MS Zoomed Spectrum

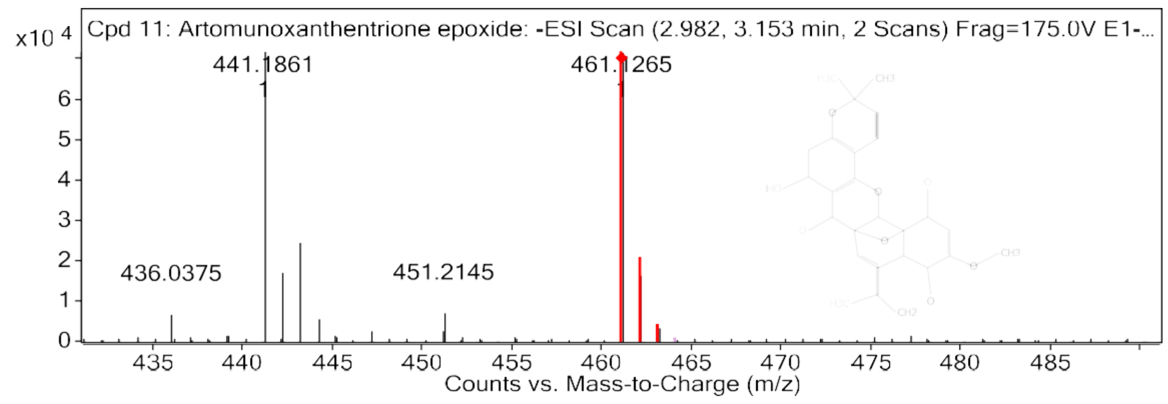

MS Spectrum Peak List

m/z	Calc m/z	Diff(ppm)	z	Abund	Formula	Ion
161.0795				27804.68		
193.9928			1	33198.33		
195.0307			1	85880.09		
197.8056				34383.05		
315.0695			1	52939.7		
441.1861			1	77303.88		
461.1265	461.1242	-5.11	1	71648.73	C26 H22 O8	(M-H)-
462.1294	462.1276	-4	1	16556.97	C26 H22 O8	(M-H)-
463.132	463.1302	-3.74	1	3725.32	C26 H22 O8	(M-H)-
497.1027			1	60200.13		

MSMS Spectrum

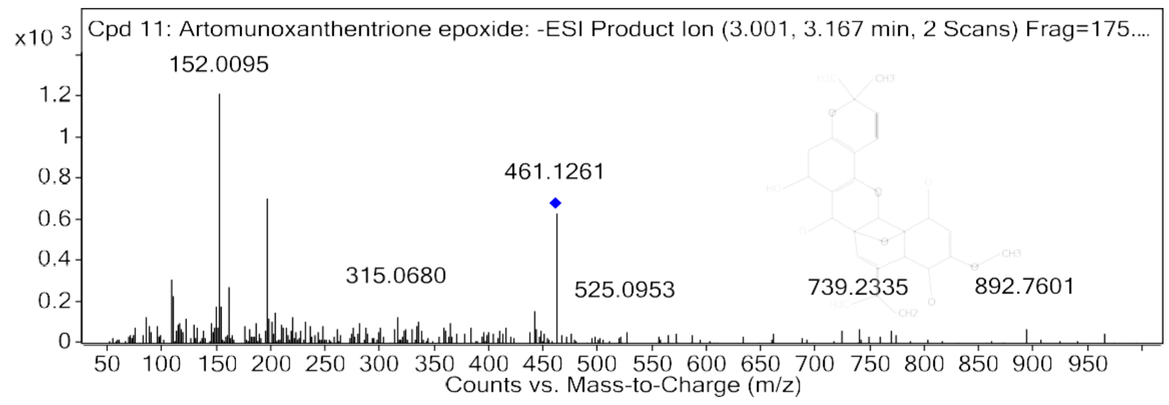

MS/MS Spectrum Peak List

m/z	z	Abund
108.02		312.27
109.0276		232.95
148.9893		181.53
152.0095	1	1216.47
153.0168	1	182.41
161.0785		275.94
195.0314		708.16
203.0803		152.62
441.1832		162.6
461.1261		634.62

Compound Structure

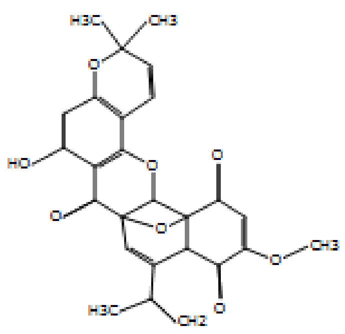

24. Eletriptan

Compound Label	Name	*m/z*	RT	Algorithm	Mass
Cpd 12: Eletriptan	**Eletriptan**	441.186	3.307	Auto MS/MS	382.1715

MS Spectrum

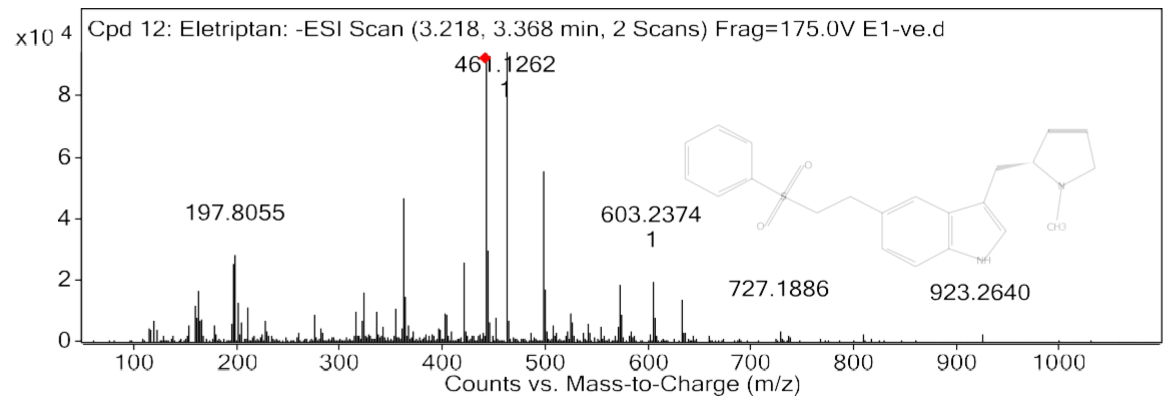

MS Zoomed Spectrum

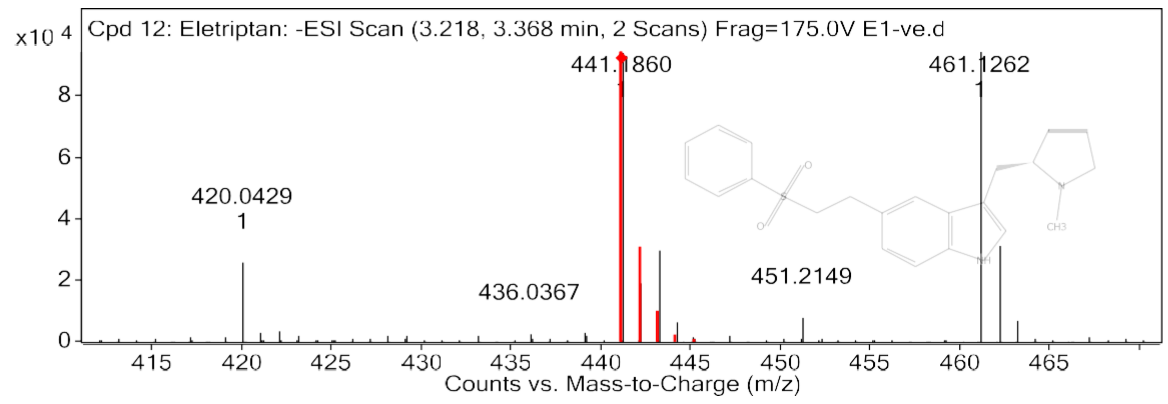

MS Spectrum Peak List

m/z	Calc m/z	Diff(ppm)	z	Abund	Formula	Ion
197.8055				28861.24		
361.0664			1	46950.53		
441.186	441.1854	-1.48	1	94318.3	C22 H26 N2 O2 S	(M+CH3COO)-
442.1893	442.1885	-1.81	1	19301.6	C22 H26 N2 O2 S	(M+CH3COO)-
443.1842	443.1862	4.67	1	30365.72	C22 H26 N2 O2 S	(M+CH3COO)-
444.1862	444.1873	2.52	1	6751.78	C22 H26 N2 O2 S	(M+CH3COO)-
445.1878	445.1888	2.37	1	1541.24	C22 H26 N2 O2 S	(M+CH3COO)-
461.1262			1	147876.52		
462.1298			1	31671.24		
497.1029			1	56111.63		

MSMS Spectrum

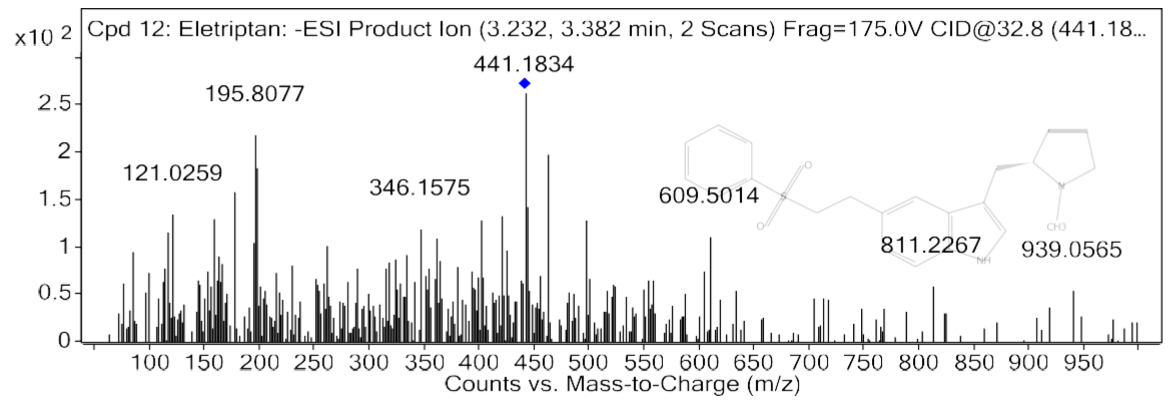

MS/MS Spectrum Peak List

m/z	z	Abund
121.0259		135.38
158.076		130.83
177.0182		159.63
195.8077		219.2
197.8064		185.07
420.0442	1	134.61
441.1834	1	263.74
442.1884	1	137.64
443.1732	1	142.77
461.1232		198.02

Compound Structure

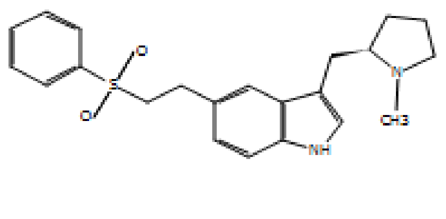

25.Fraxidin

Compound Label	Name	*m/z*	RT	Algorithm	Mass
Cpd 14: Fraxidin	**Fraxidin**	221.0463	3.883	Auto MS/MS	222.0532

MS Spectrum

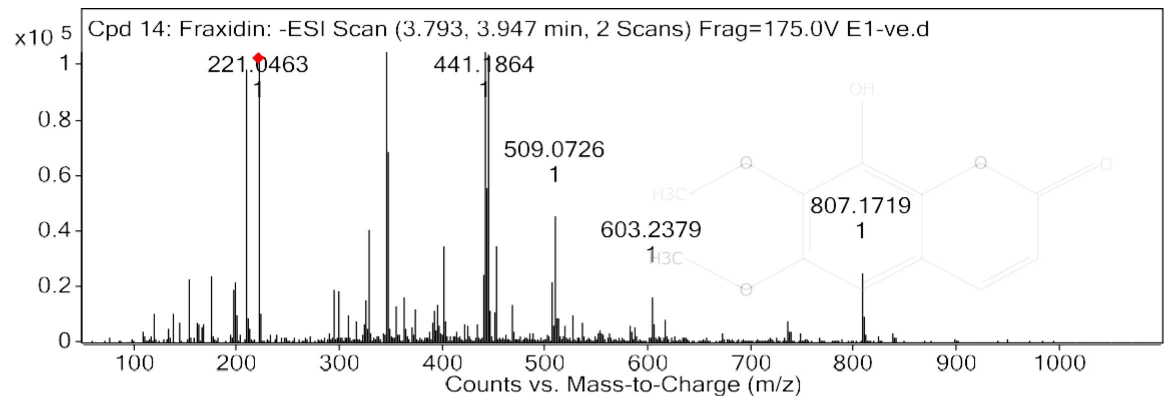

MS Zoomed Spectrum

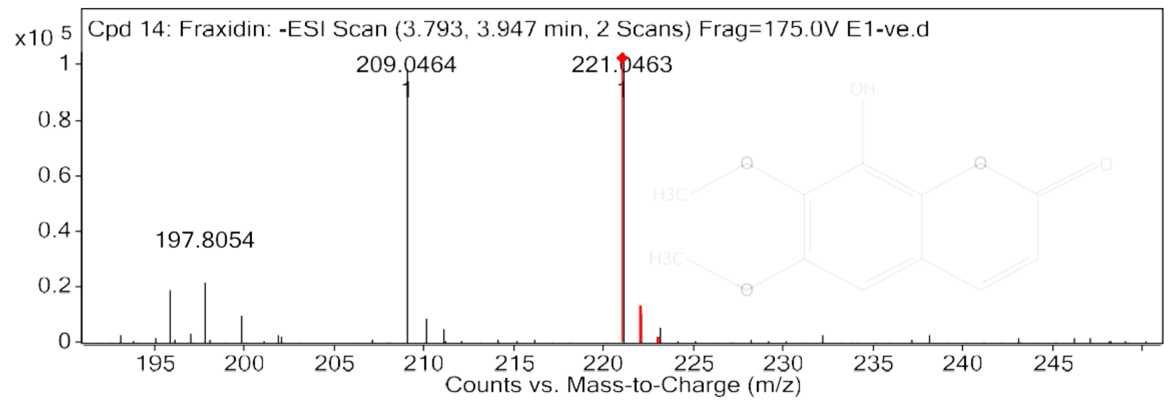

MS Spectrum Peak List

m/z	Calc m/z	Diff(ppm)	z	Abund	Formula	Ion
209.0464			1	98559.92		
221.0463	221.0455	-3.52	1	104564.13	C11 H10 O5	(M-H)-
222.0497	222.0489	-3.64	1	10962.22	C11 H10 O5	(M-H)-
223.0433	223.0508	33.53	1	5891.25	C11 H10 O5	(M-H)-
344.037			1	166517.48		
346.034			1	68745.25		
441.1864			1	262928.13		
442.1891			1	56320.05		
443.1849			1	104119.79		
509.0726			1	45852.65		

MSMS Spectrum

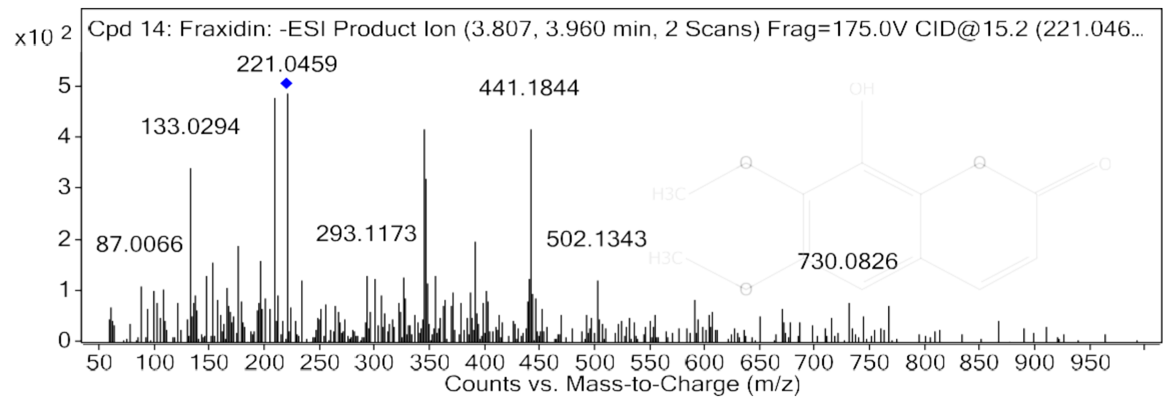

MS/MS Spectrum Peak List

m/z	z	Abund
133.0294		342.31
152.0104		156.6
175.0594		188.55
195.8017		161.11
209.0457		478.5
221.0459		488.97
344.0349	1	416.57
346.0275	1	320.72
391.0729	1	197.41
441.1844	1	418.13

Compound Structure

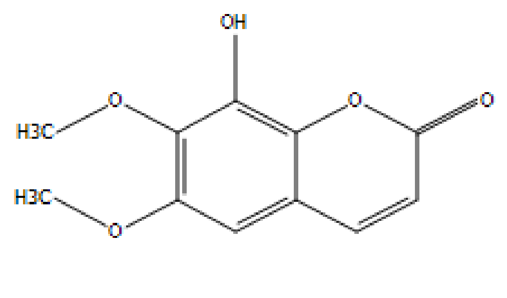

26.N-(6-Oxo-6H-dibenzo[b,d]pyran-3- yl)maleamic acid

Compound Label	Name	*m/z*	RT	Algorithm	Mass
Cpd 17: N-(6-Oxo-6H-dibenzo[b,d]pyran-3-yl)maleamic acid	**N-(6-Oxo-6H-dibenzo[b,d]pyran-3-yl)maleamic acid**	354.0659	4.262	Auto MS/MS	309.067

MS Spectrum

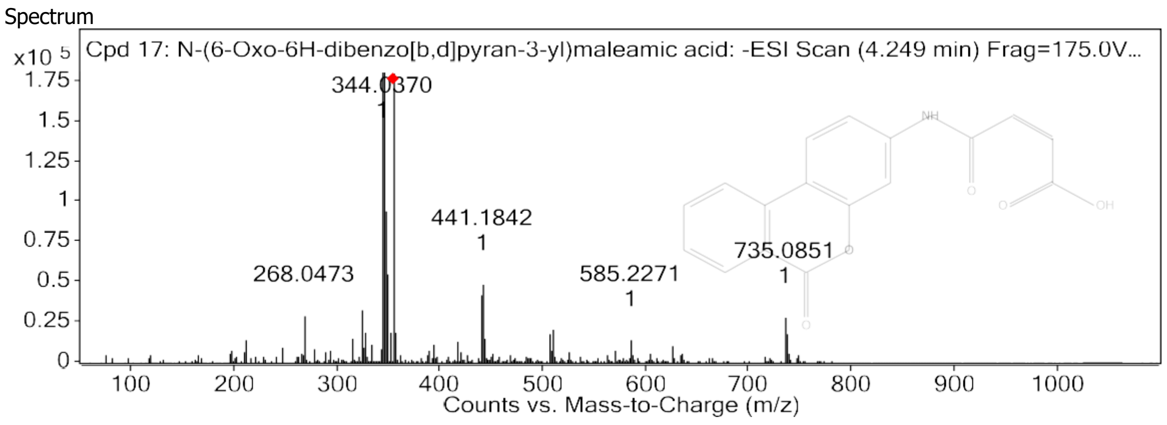

MS Zoomed Spectrum

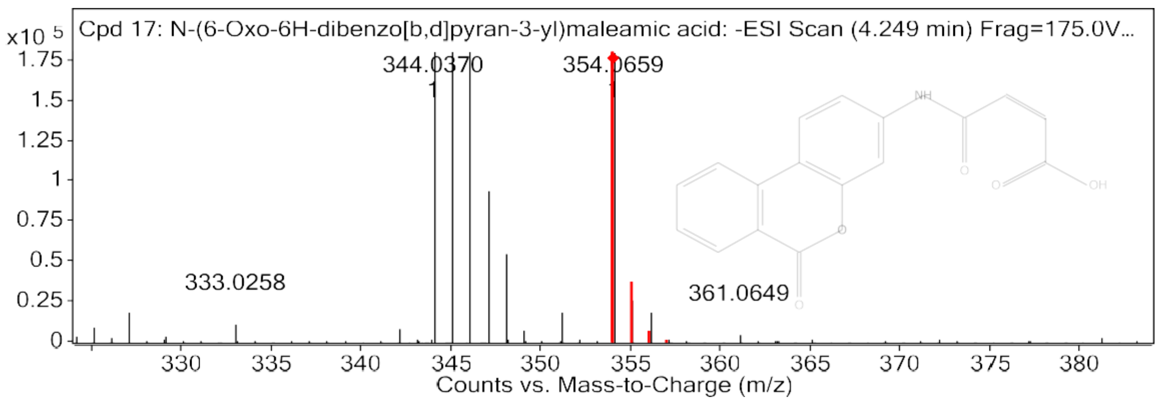

MS Spectrum Peak List

m/z	Calc m/z	Diff(ppm)	z	Abund	Formula	Ion
344.037			1	1781311.25		
345.0401			1	235815.33		
346.0342			1	744729.81		
347.037			1	95294.2		
348.0315			1	56077.88		
354.0659	354.0619	-11.09	1	180457.14	C17 H11 N O5	(M+HCOO)-
355.0691	355.0652	-11.11	1	27037.23	C17 H11 N O5	(M+HCOO)-
356.0636	356.0675	10.81	1	19933.35	C17 H11 N O5	(M+HCOO)-
357.0728	357.0701	-7.55	1	3110	C17 H11 N O5	(M+HCOO)-
441.1842			1	49406		

MSMS Spectrum

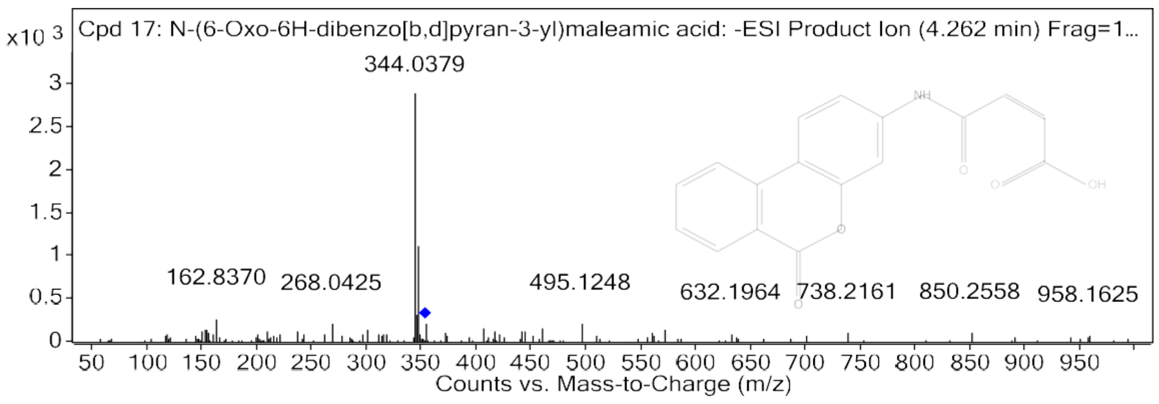

MS/MS Spectrum Peak List

m/z	z	Abund
162.837		282
268.0425		217.72
344.0379	1	2906.67
345.038	1	328.92
346.0337	1	1132.69
347.0355	1	223.25
354.0649		224.53
406.0283		170.22
460.1032		180.87
495.1248		221.7

Compound Structure

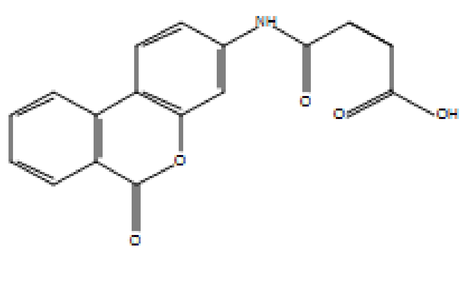

27.Sciadopitysin

Compound Label	Name	m/z	RT	Algorithm	Mass
Cpd 20: Sciadopitysin	**Sciadopitysin**	625.1373	5.015	Auto MS/MS	580.1389

MS Spectrum

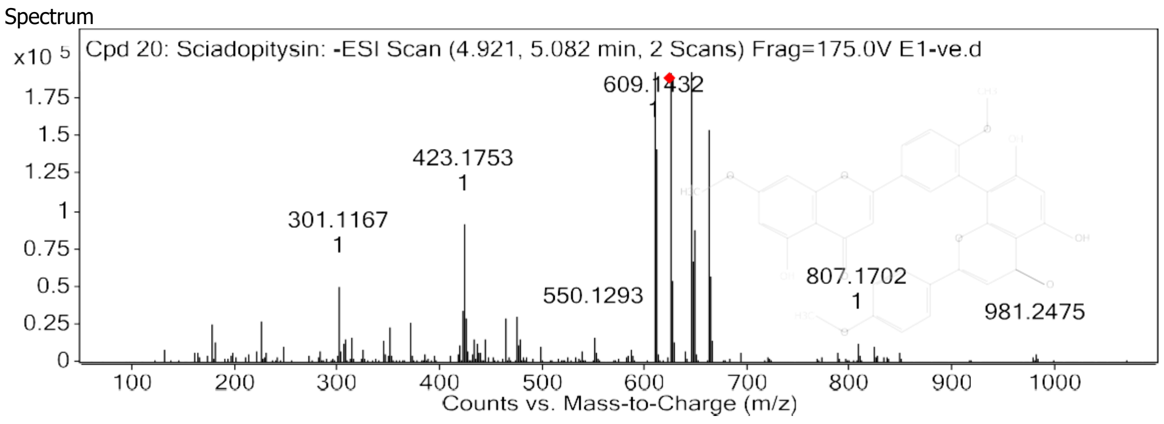

MS Zoomed Spectrum

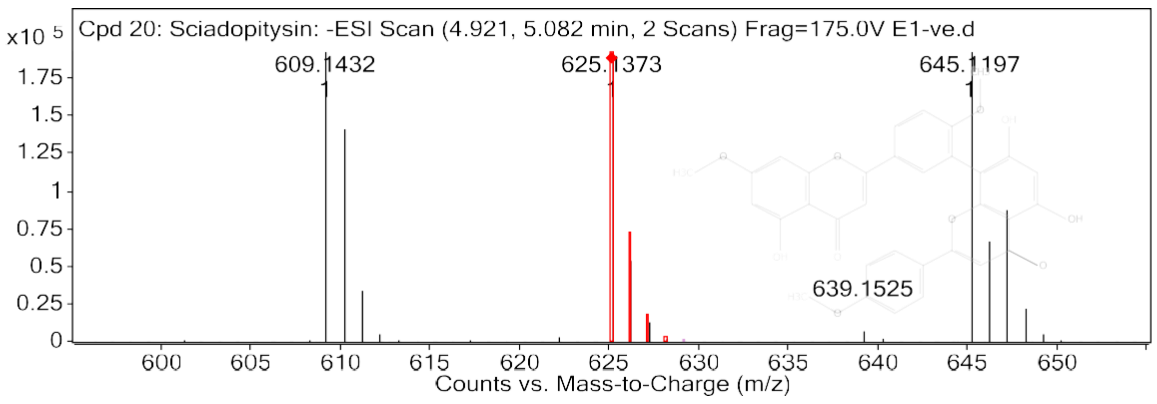

MS Spectrum Peak List

m/z	Calc m/z	Diff(ppm)	z	Abund	Formula	Ion
423.1753			1	92355.78		
609.1432			1	486394.09		
610.1463			1	142020.11		
625.1373	625.1351	-3.4	1	193169.13	33 H24 O10	(M+HCOO)-
626.1401	626.1385	-2.48	1	55206.25	33 H24 O10	(M+HCOO)-
627.1427	627.1413	-2.38	1	13833.61	33 H24 O10	(M+HCOO)-
628.1441	628.144	-0.19	1	3127.81	33 H24 O10	(M+HCOO)-
645.1197			1	239242.91		
647.1179			1	88560.55		
661.1137			1	155282.06		

MSMS Spectrum

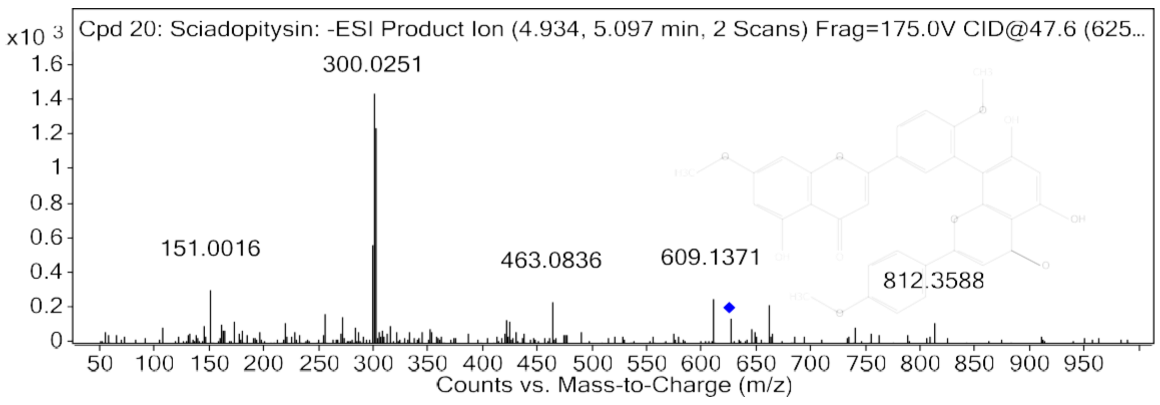

MS/MS Spectrum Peak List

m/z	z	Abund
151.0016		309.76
255.0261		168.84
271.0185		157.53
299.0162		569.87
300.0251		1447.18
301.0318		1248.88
463.0836		241.09
609.1371		258.41
625.1397		148.79
661.1134	1	227.33

Compound Structure

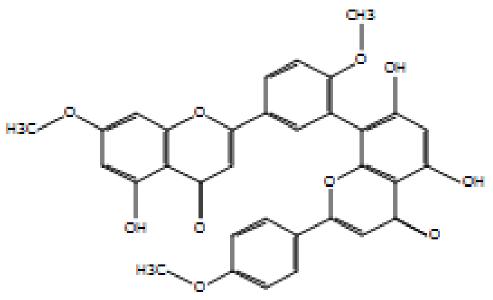

28.Rutin

Compound Label	Name	*m/z*	RT	Algorithm	Mass
Cpd 26: Rutin	**Rutin**	609.1434	5.308	Auto MS/MS	610.1505

MS Spectrum

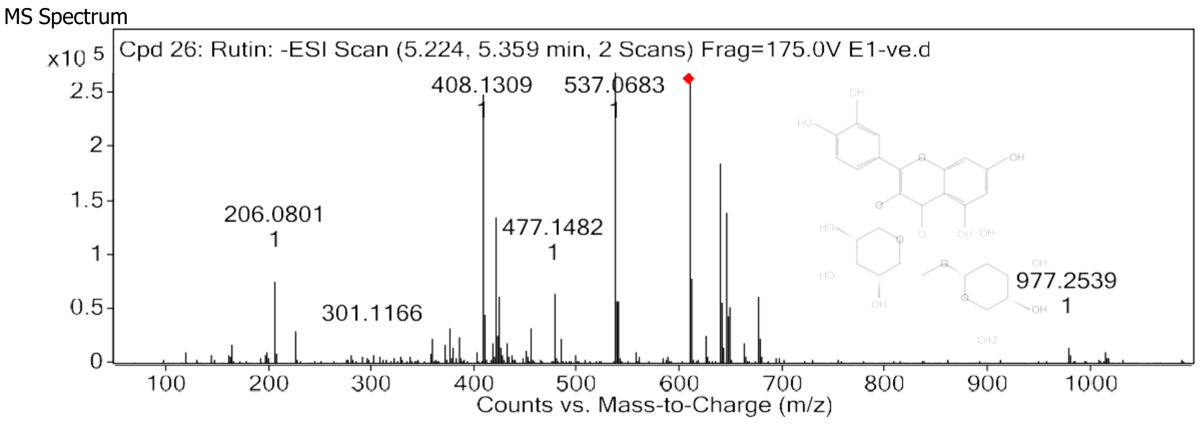

MS Zoomed Spectrum

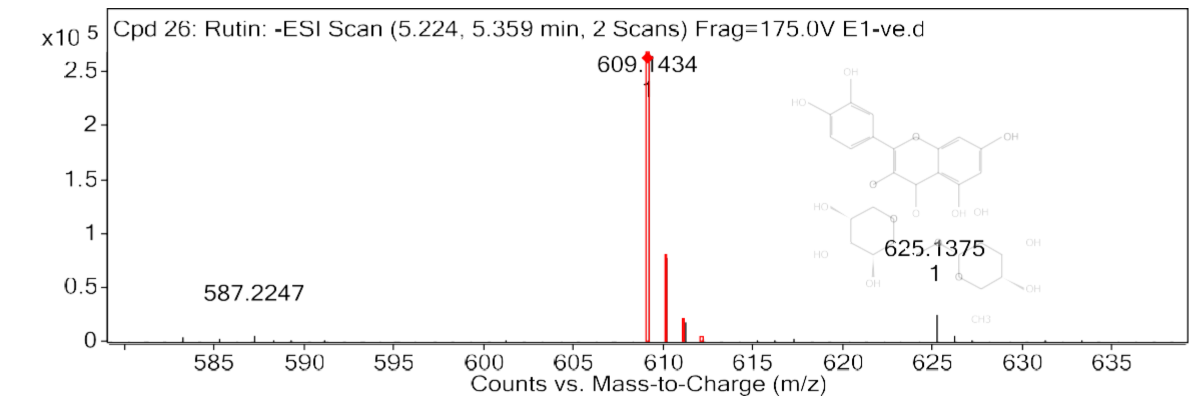

MS Spectrum Peak List

m/z	Calc m/z	Diff(ppm)	z	Abund	Formula	Ion
206.0801			1	76599.42		
408.1309			1	248513.59		
421.1608			1	135882.22		
537.0683			1	279047.63		
609.1434	609.1461	4.38	1	268490.88	C27 H30 O16	(M-H)-
610.1462	610.1495	5.42	1	79427.65	C27 H30 O16	(M-H)-
611.1488	611.1518	4.98	1	19295.77	C27 H30 O16	(M-H)-
612.1513	612.1545	5.26	1	3986.23	C27 H30 O16	(M-H)-
639.1538			1	185015.75		
645.1196			1	139684.78		

MSMS Spectrum

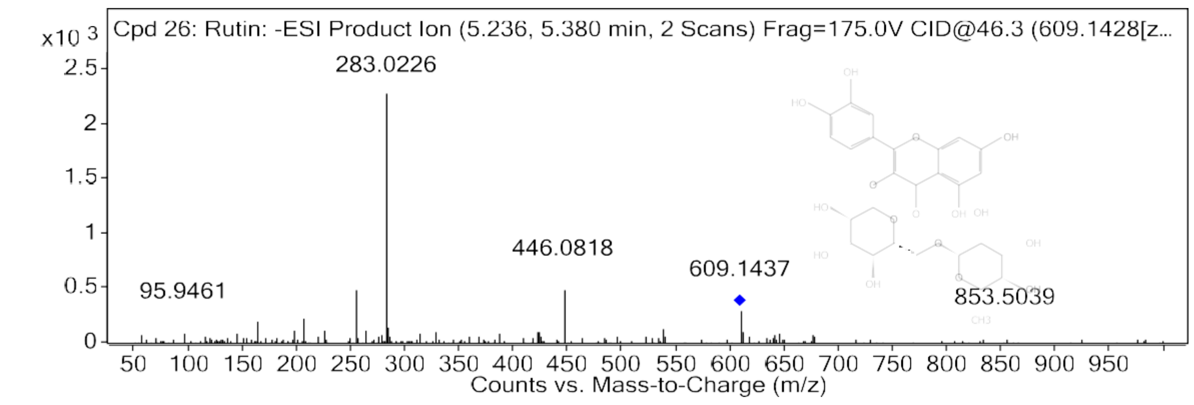

MS/MS Spectrum Peak List

m/z	z	Abund
164.0698		199.96
206.0783		227.37
255.028		496.6
283.0226	1	2288.8
284.0336	1	150.92
446.0818	1	495.49
447.0906	1	257.93
537.0607		143.72
538.0663	1	141.99
609.1437	1	304.63

Compound Structure

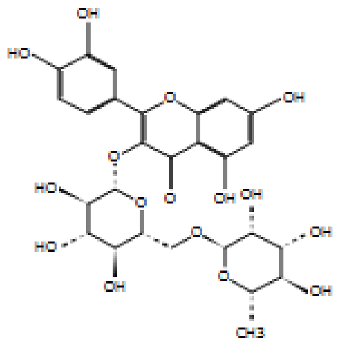

29.5'- Butyrylphosphoinosine

Compound Label	Name	m/z	RT	Algorithm	Mass
Cpd 29: 5'-Butyrylphosphoinosine	**5'-Butyrylphosphoinosine**	463.0856	5.735	Auto MS/MS	418.0875

MS Spectrum

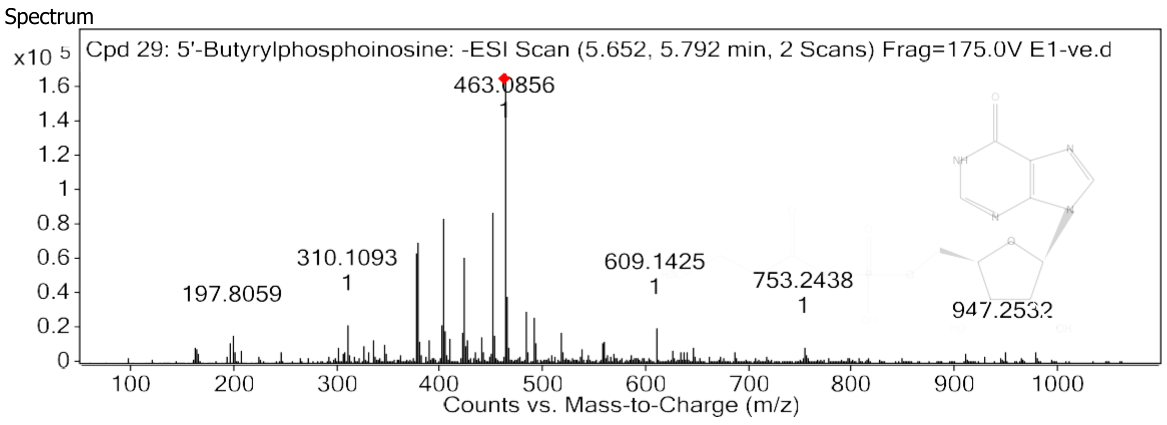

MS Zoomed Spectrum

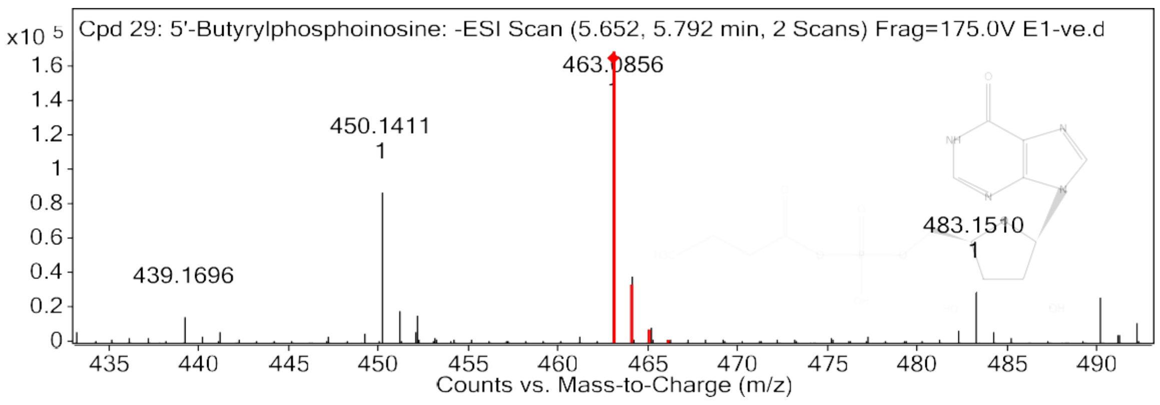

MS Spectrum Peak List

m/z	Calc m/z	Diff(ppm)	z	Abund	Formula	Ion
376.0093			1	64272.88		
378.12			1	70394.77		
403.1947			1	83906.95		
422.1468				61528.07		
423.1726			1	43029.25		
450.1411			1	87592.06		
463.0856	463.0872	3.49	1	168353.58	C14 H19 N4 O9 P	(M+HCOO)-
464.0886	464.0901	3.11	1	39317.38	C14 H19 N4 O9 P	(M+HCOO)-
465.0922	465.092	-0.26	1	9575.1	C14 H19 N4 O9 P	(M+HCOO)-
466.0949	466.0946	-0.59	1	1821.6	C14 H19 N4 O9 P	(M+HCOO)-

MSMS Spectrum

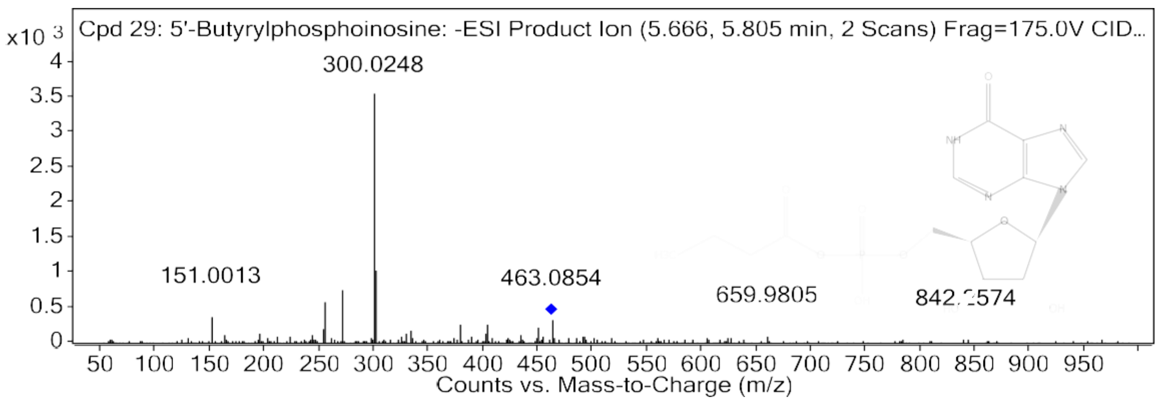

MS/MS Spectrum Peak List

m/z	z	Abund
151.0013		378.1
254.0195		222.73
255.0273		595.43
271.0222		777.47
300.0248	1	3567.98
301.0327	1	1040.34
378.1209		282.03
403.1903		281.69
450.1428		233.27
463.0854	1	344.45

Compound Structure

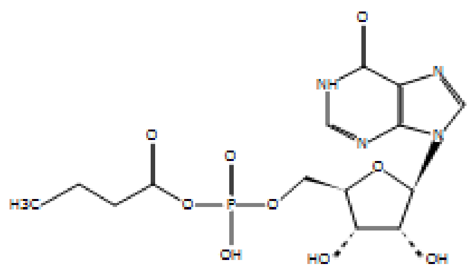

30.Evoxine

Compound Label	Name	*m/z*	RT	Algorithm	Mass
Cpd 30: Evoxine	**Evoxine**	392.136	6.06	Auto MS/MS	347.1374

MS Spectrum

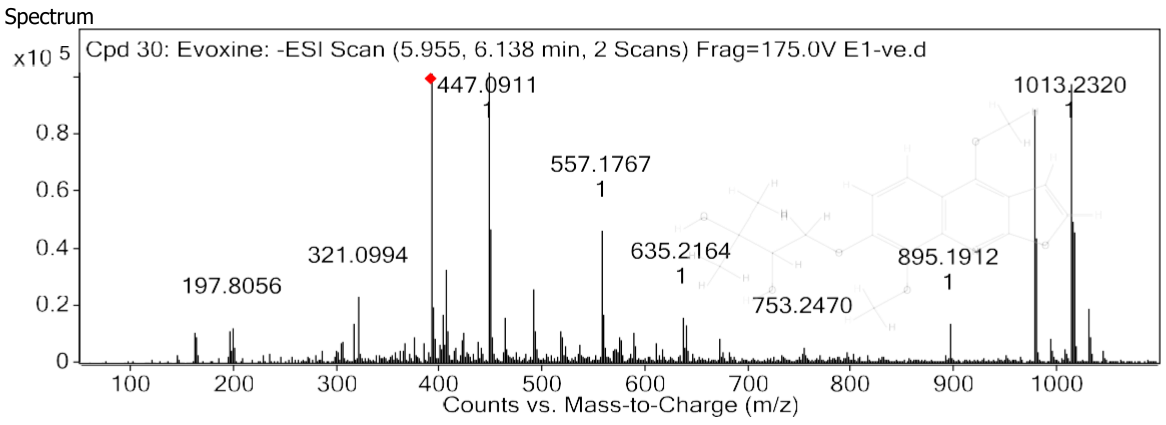

MS Zoomed Spectrum

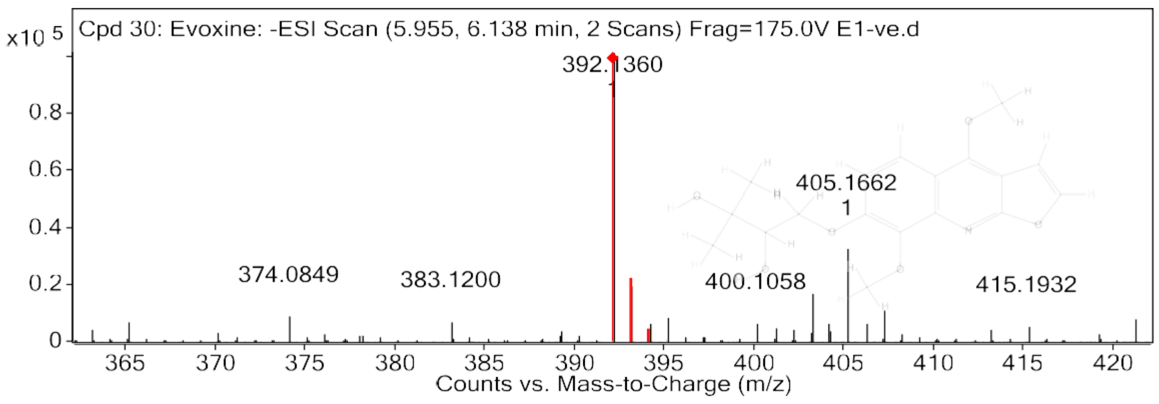

MS Spectrum Peak List

m/z	Calc m/z	Diff(ppm)	z	Abund	Formula	Ion
392.136	392.1351	-2.21	1	101463.36	C18 H21 N O6	(M+HCOO)-
393.1389	393.1384	-1.33	1	19816.52	C18 H21 N O6	(M+HCOO)-
394.1353	394.1407	13.57	1	7022.82	C18 H21 N O6	(M+HCOO)-
447.0911			1	219811.3		
448.0939			1	47106.62		
557.1767			1	46527.59		
977.2553			1	88657.95		
1013.232			1	97728.96		
1014.2352			1	49645.54		
1015.2325			1	45817.61		

MSMS Spectrum

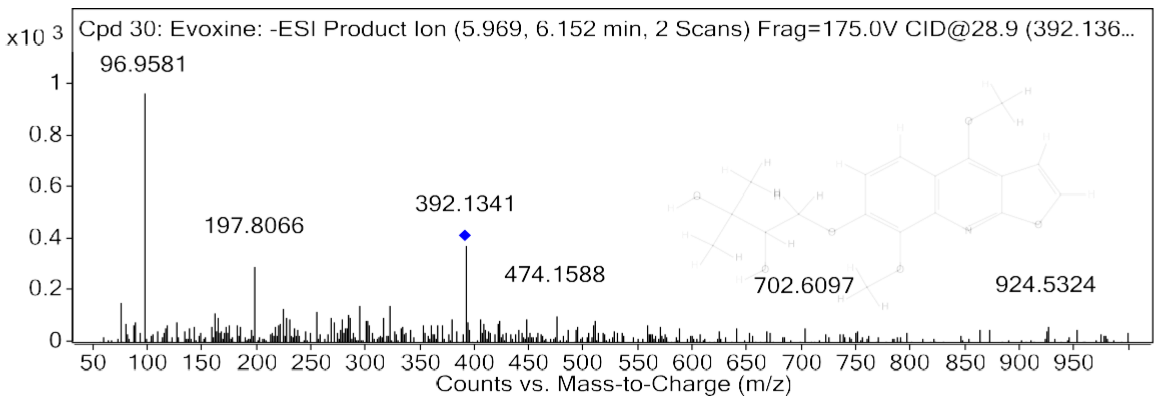

MS/MS Spectrum Peak List

m/z	z	Abund
74.9898		156.87
96.9581		969.11
160.8396		116.05
197.8066		293.88
224.5282		131.94
255.0275		122.36
284.032		108.49
294.1638		146.54
321.1004	1	145.15
392.1341	1	378.49

Compound Structure

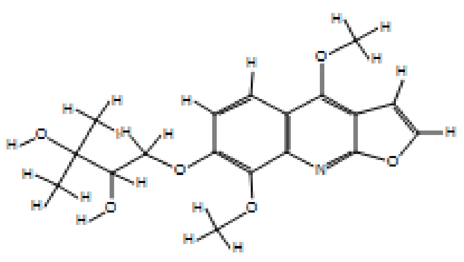

31.Kaempferol 3-O-β-D- galactoside

Compound Label	Name	m/z	RT	Algorithm	Mass
Cpd 32: Kaempferol 3-O-β-D-galactoside	**Kaempferol 3-O-β-D-galactoside**	447.0911	6.223	Auto MS/MS	448.0983

MS Spectrum

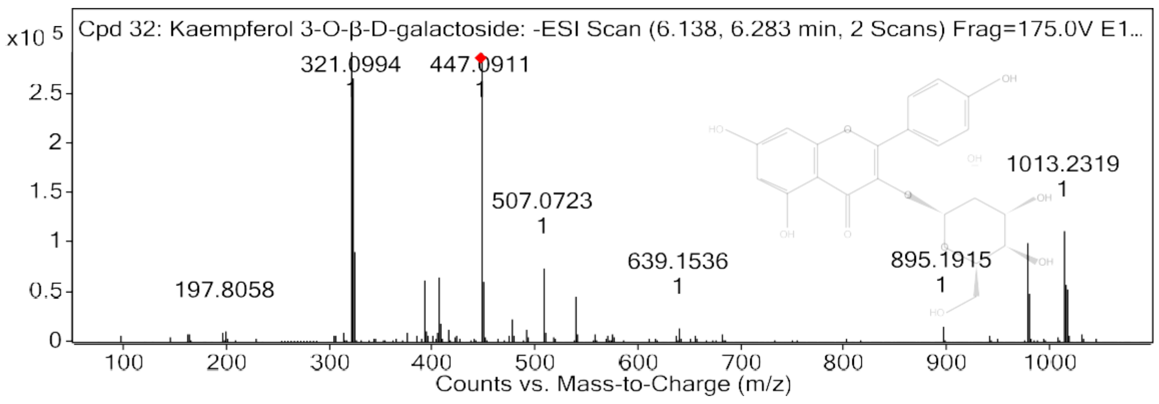

MS Zoomed Spectrum

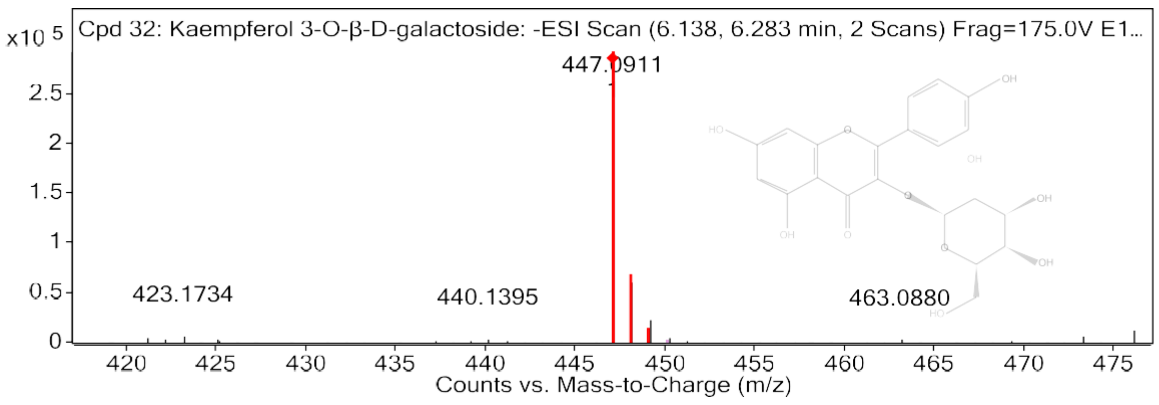

MS Spectrum Peak List

m/z	Calc m/z	Diff(ppm)	z	Abund	Formula	Ion
321.0994			1	1882519.75		
322.1026			1	266648.13		
323.0991			1	91824.42		
405.166			1	65941.68		
447.0911	447.0933	4.99	1	291528.31	C21 H20 O11	(M-H)-
448.094	448.0967	5.98	1	61303.89	C21 H20 O11	(M-H)-
449.0972	449.0989	3.79	1	13844.99	C21 H20 O11	(M-H)-
507.0723			1	75828.09		
977.2552			1	100538.7		
1013.2319			1	112976.65		

MSMS Spectrum

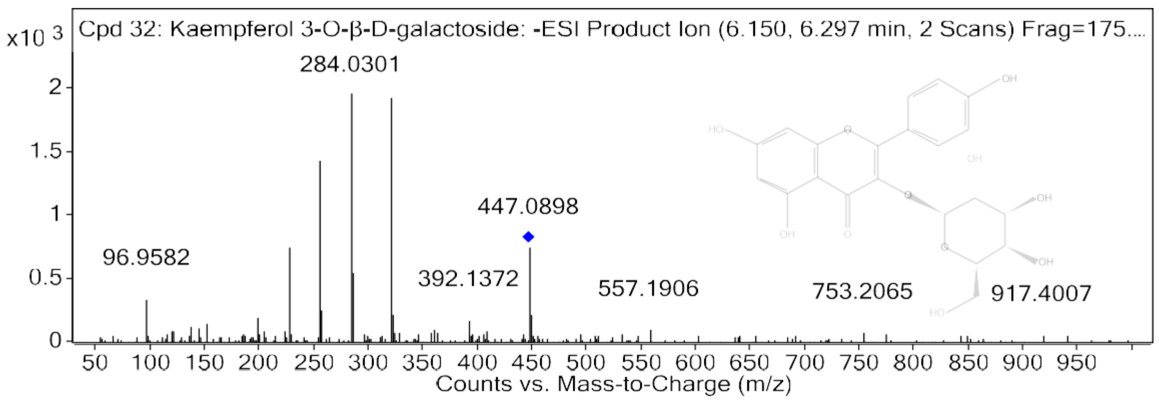

MS/MS Spectrum Peak List

m/z	z	Abund
96.9582		346.66
227.0317	1	760.01
255.0277	1	1443.03
256.0335	1	255.09
284.0301	1	1972.1
285.0373	1	555.45
321.0999	1	1938.76
322.1017	1	224.67
447.0898	1	750.25
448.0952	1	222.12

Compound Structure

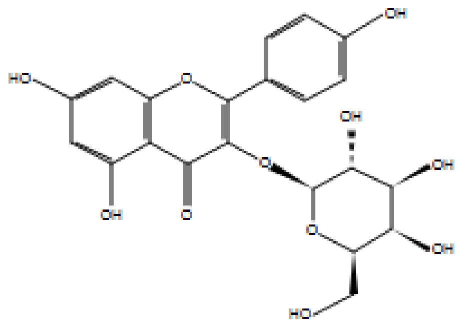

32.Lactucin

Compound Label	Name	m/z	RT	Algorithm	Mass
Cpd 33: Lactucin	**Lactucin**	321.0994	6.227	Auto MS/MS	276.1009

MS Spectrum

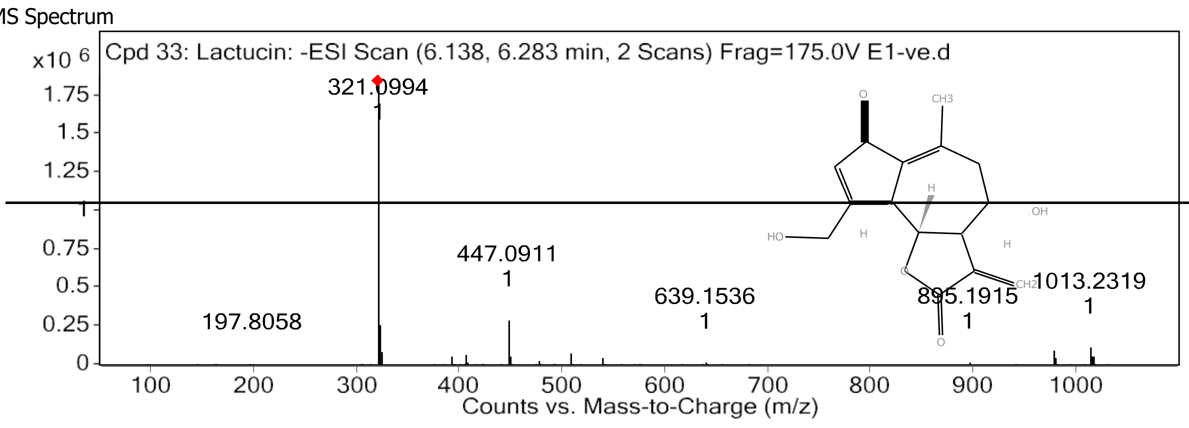

MS Zoomed Spectrum

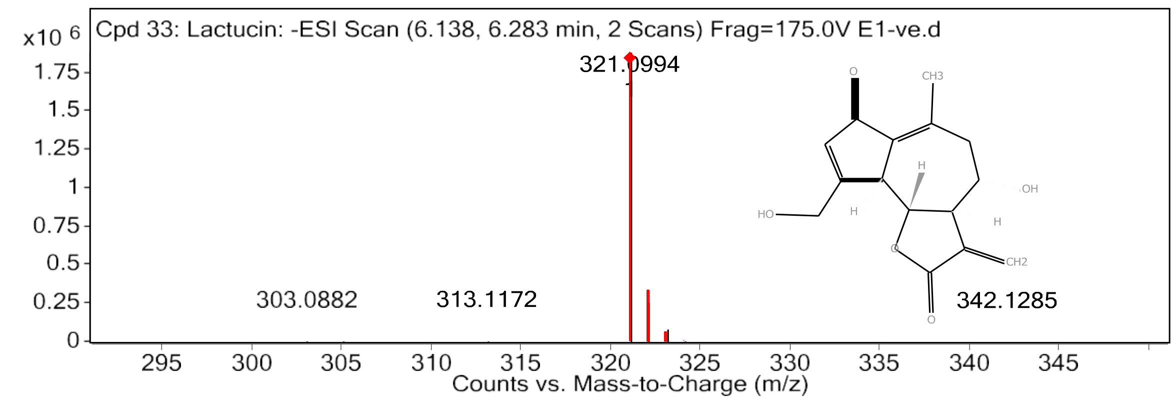

MS Spectrum Peak List

m/z	Calc m/z	Diff(ppm)	z	Abund	Formula	Ion
321.0994	321.098	-4.43	1	1882519.75	C15 H16 O5	(M+HCOO)-
322.1026	322.1014	-3.74	1	266648.13	C15 H16 O5	(M+HCOO)-
323.0991	323.1035	13.7	1	91824.42	C15 H16 O5	(M+HCOO)-
392.1361			1	62885		
405.166			1	65941.68		
447.0911			1	291528.31		
448.094			1	61303.89		
507.0723			1	75828.09		
977.2552			1	100538.7		
1013.2319			1	112976.65		

MSMS Spectrum

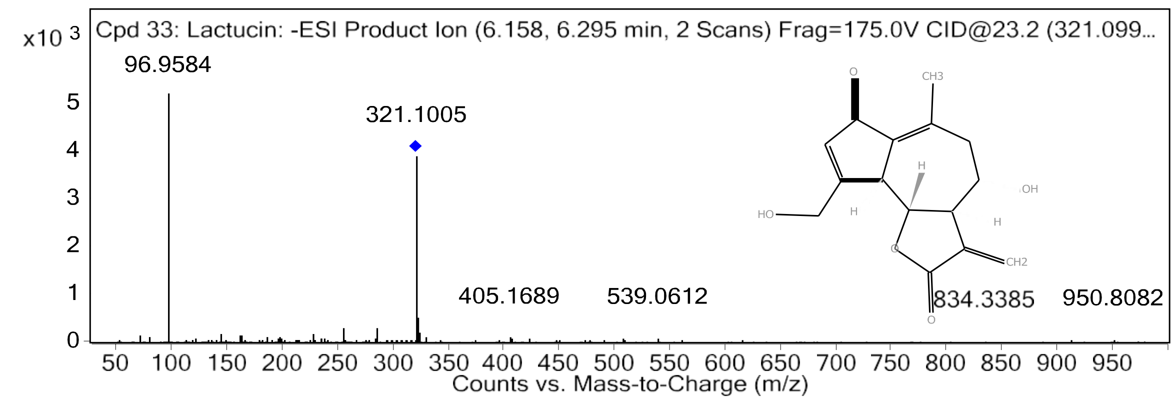

MS/MS Spectrum Peak List

m/z	z	Abund
96.9584		5236.34
144.0427		172.77
160.8387		166.52
162.8373		170.82
227.0327		203.13
255.029		316.17
284.0279	1	317.38
321.1005	1	3926.51
322.1052	1	538.88
323.0989	1	225.77

Compound Structure

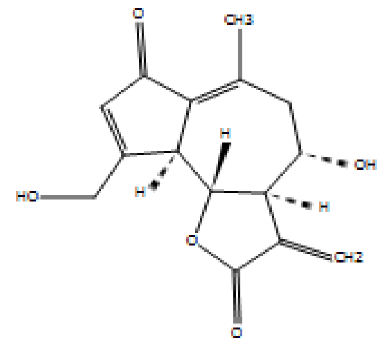

33.1,4-Dimethoxyglucobrassic

Compound Label	Name	*m/z*	RT	Algorithm	Mass
Cpd 34: 1,4-Dimethoxyglucobrassicin	**1,4-Dimethoxyglucobrassic**	507.0722	6.294	Auto MS/MS	508.0794

MS Spectrum

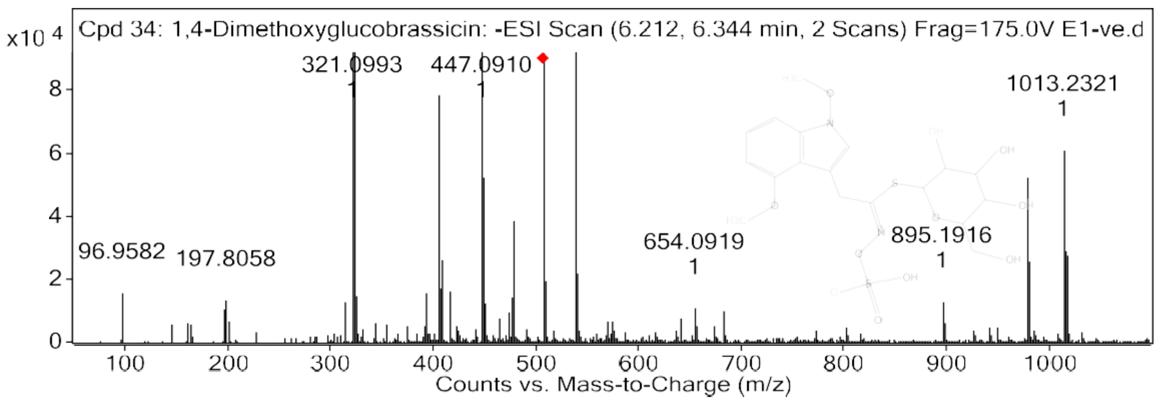

MS Zoomed Spectrum

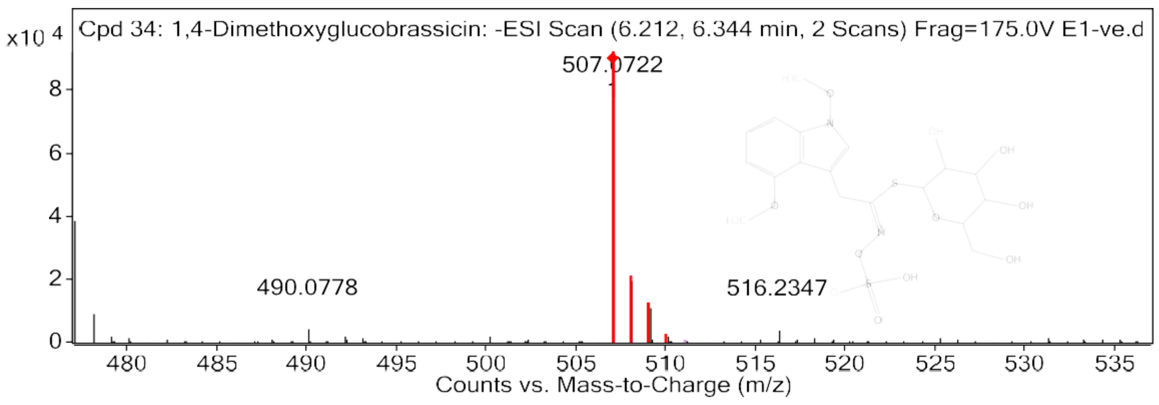

MS Spectrum Peak List

m/z	Calc m/z	Diff(ppm)	z	Abund	Formula	Ion
321.0993			1	2257272.25		
322.1026			1	321526.75		
323.0988			1	113126.81		
405.1662			1	78841.05		
447.091			1	250790.5		
507.0722	507.0749	5.28	1	92027.79	C18 H24 N2 O11 S2	(M-H)-
508.0749	508.0778	5.66	1	19701.67	C18 H24 N2 O11 S2	(M-H)-
509.0706	509.0738	6.38	1	11522.62	C18 H24 N2 O11 S2	(M-H)-
510.0757	510.076	0.59	1	2465.38	C18 H24 N2 O11 S2	(M-H)-
538.0525			1	113979.89		

MSMS Spectrum

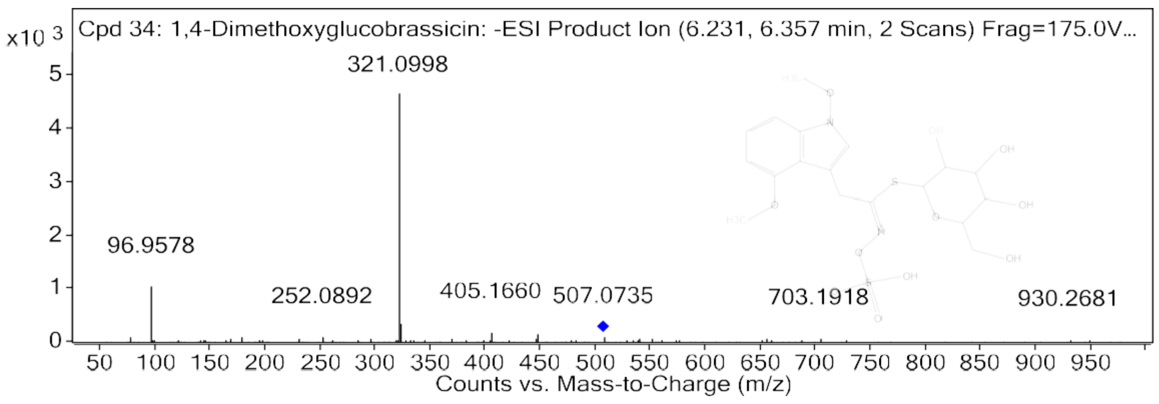

MS/MS Spectrum Peak List

m/z	z	Abund
77.3809		108.91
95.9501		600.26
96.9578		1050.12
179.1062		105.29
252.0892		102.34
321.0998	1	4678.56
322.1041	1	653.3
323.1	1	362.08
405.166		188.41
447.0866		162.26

Compound Structure

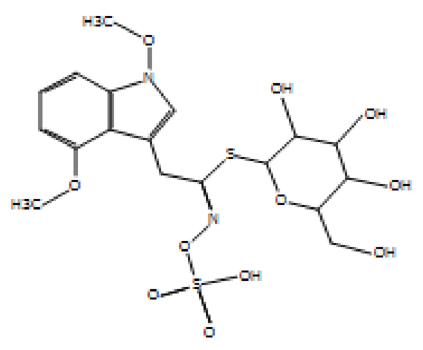

34.Pubesenolide

Compound Label	Name	*m/z*	RT	Algorithm	Mass
Cpd 39: Pubesenolide	**Pubesenolide**	457.2891	7.746	Auto MS/MS	458.2963

MS Spectrum

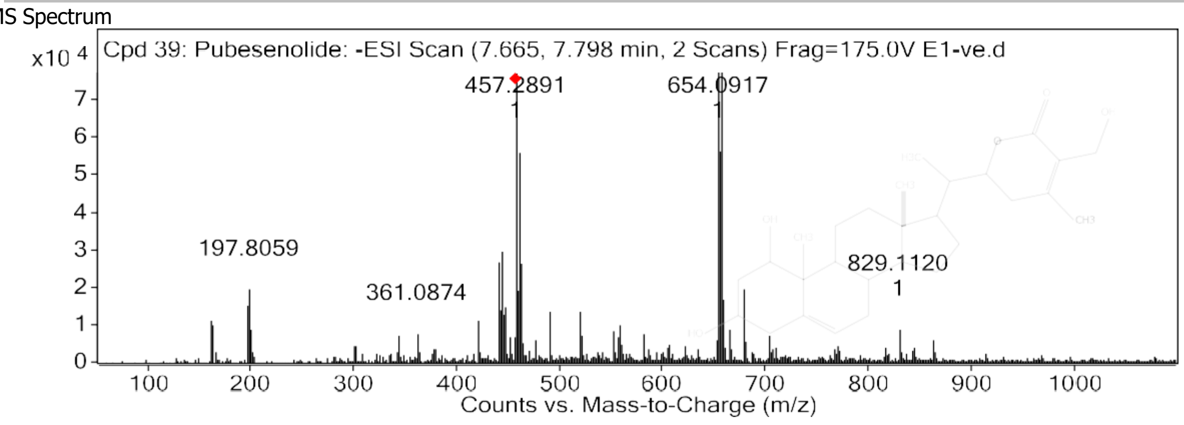

MS Zoomed Spectrum

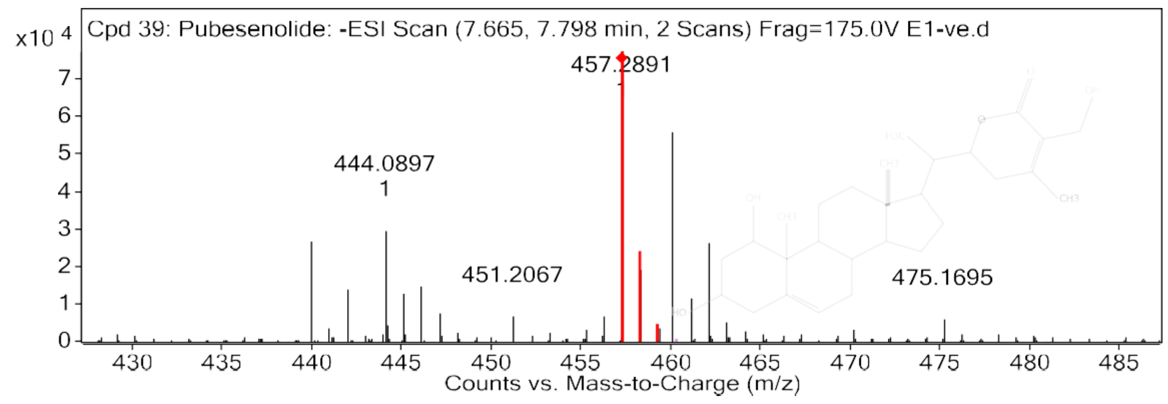

MS Spectrum Peak List

m/z	Calc m/z	Diff(ppm)	z	Abund	Formula	Ion
439.9531			1	27004.87		
444.0897			1	30044.89		
457.2891	457.2959	14.99	1	77217.66	C28 H42 O5	(M-H)-
458.2922	458.2994	15.51	1	19364.13	C28 H42 O5	(M-H)-
459.294	459.3023	18.07	1	4062.35	C28 H42 O5	(M-H)-
460.0667			1	56242.33		
654.0917			1	201302.98		
655.0941			1	56405.41		
656.0887			1	105396.64		
657.0912			1	27850.63		

MSMS Spectrum

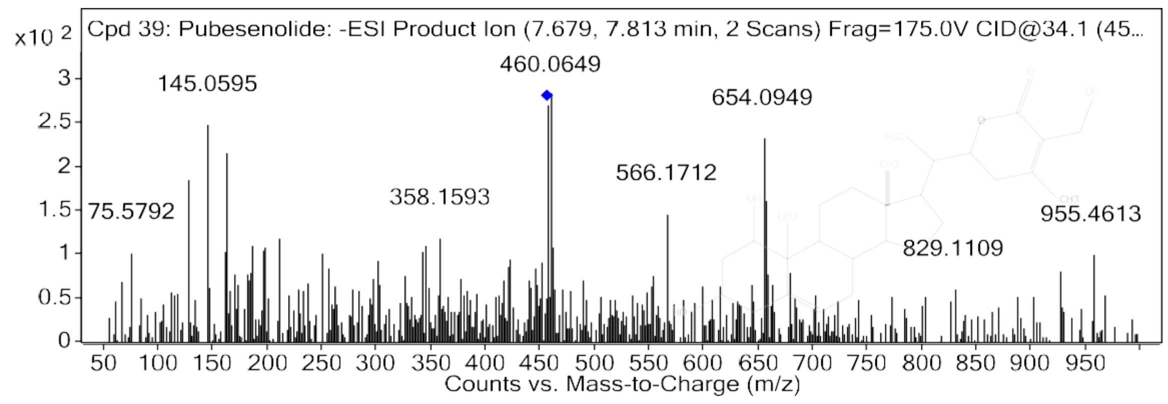

MS/MS Spectrum Peak List

m/z	z	Abund
127.0483		186.17
145.0595		249.04
162.8371		216.61
210.1452		119.41
358.1593		119.31
457.2847	1	270.86
460.0649	1	285.55
566.1712		147.09
654.0949	1	233.24
656.0862	1	162.44

Compound Structure

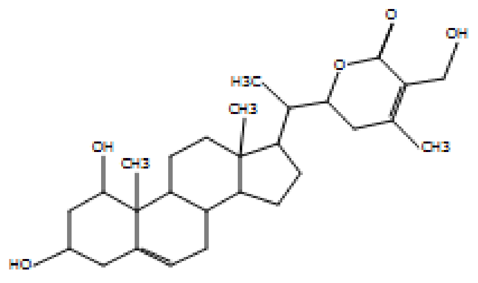

35. Corchorifatty acid F

Compound Label	Name	*m/z*	RT	Algorithm	Mass
Cpd 43: Corchorifatty acid F	**Corchorifatty acid F**	327.2156	8.681	Auto MS/MS	328.2228

MS Spectrum

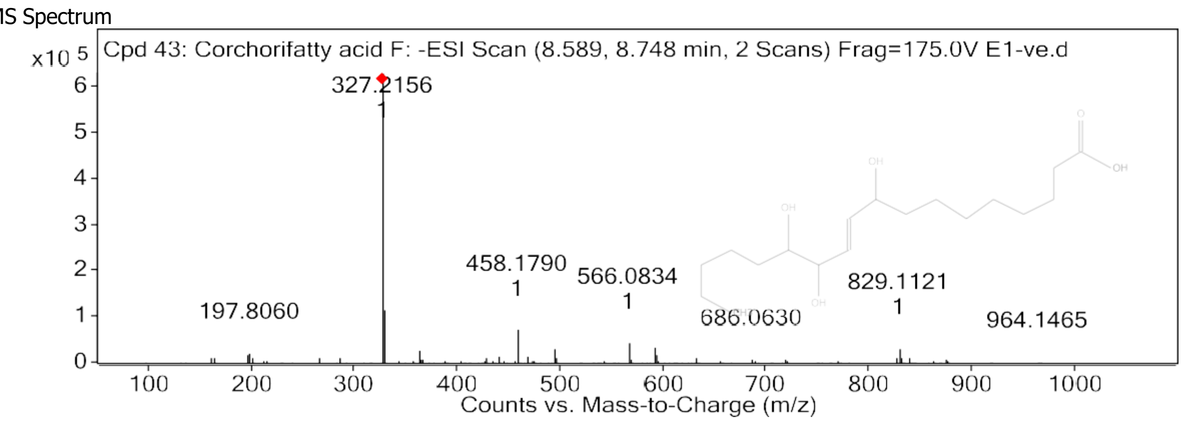

MS Zoomed Spectrum

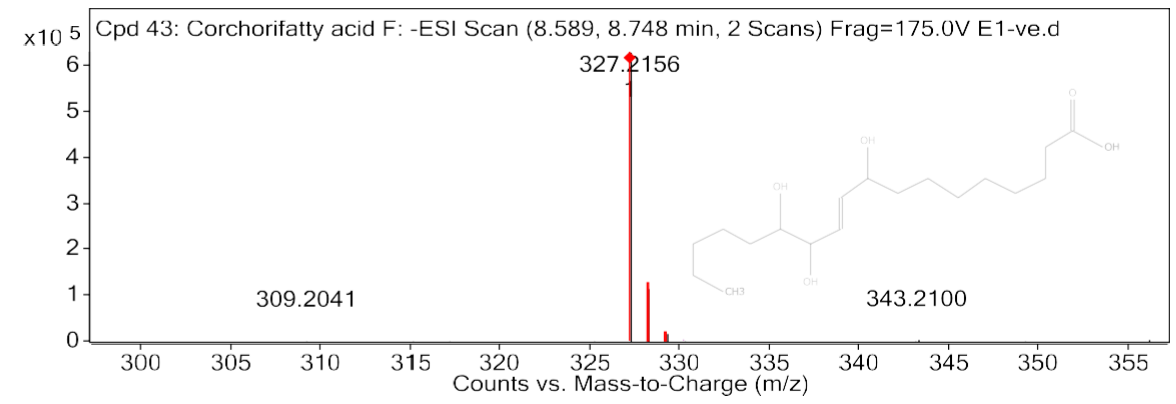

MS Spectrum Peak List

m/z	Calc m/z	Diff(ppm)	z	Abund	Formula	Ion
197.806				23814.24		
327.2156	327.2177	6.44	1	630405.63	C18 H32 O5	(M-H)-
328.2187	328.2211	7.31	1	116777.13	C18 H32 O5	(M-H)-
329.2216	329.2236	6.01	1	17882.08	C18 H32 O5	(M-H)-
363.1915			1	28585.45		
458.179			1	73321.3		
494.1557			1	33016.05		
566.0834			1	45214.27		
591.1071			1	36168.6		
829.1121			1	33373.48		

MSMS Spectrum

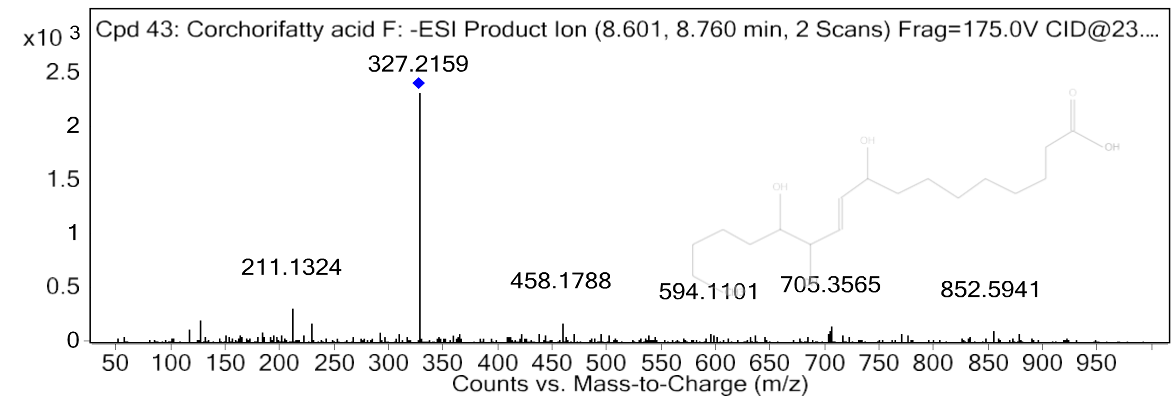

MS/MS Spectrum Peak List

m/z	z	Abund
116.9971		127.83
127.11		210.31
211.1324		314.35
229.1424		177.11
327.2159	1	2318.47
328.2213	1	335.09
458.1788	1	186.95
703.367		105.01
705.3565		152.11
852.5941		106.85

Compound Structure

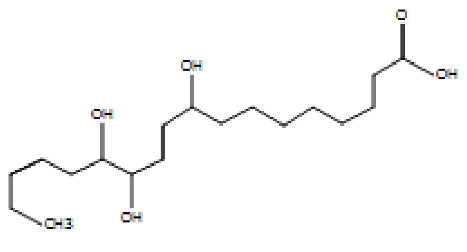

36.Linifolin A

Compound Label	Name	*m/z*	RT	Algorithm	Mass
Cpd 45: Linifolin A	**Linifolin A**	349.1304	8.903	Auto MS/MS	304.1319

MS Spectrum

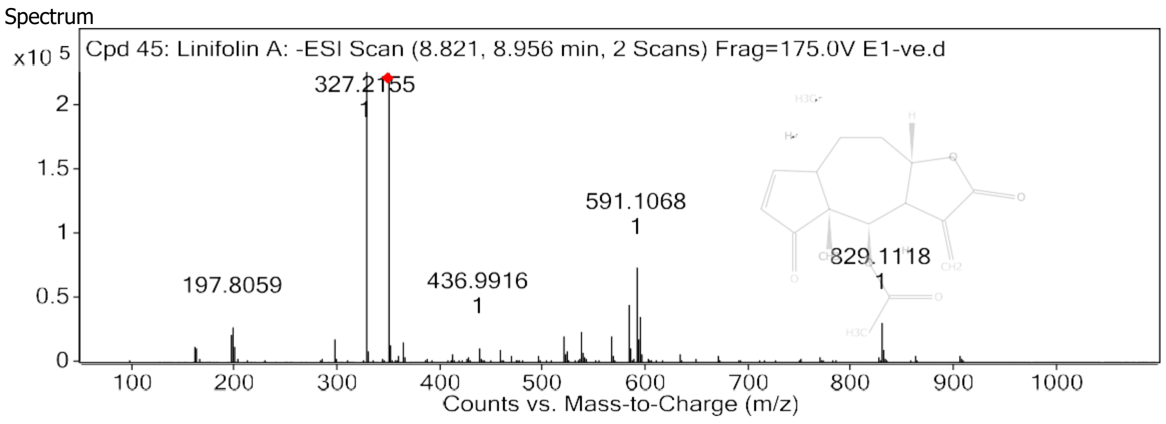

MS Zoomed Spectrum

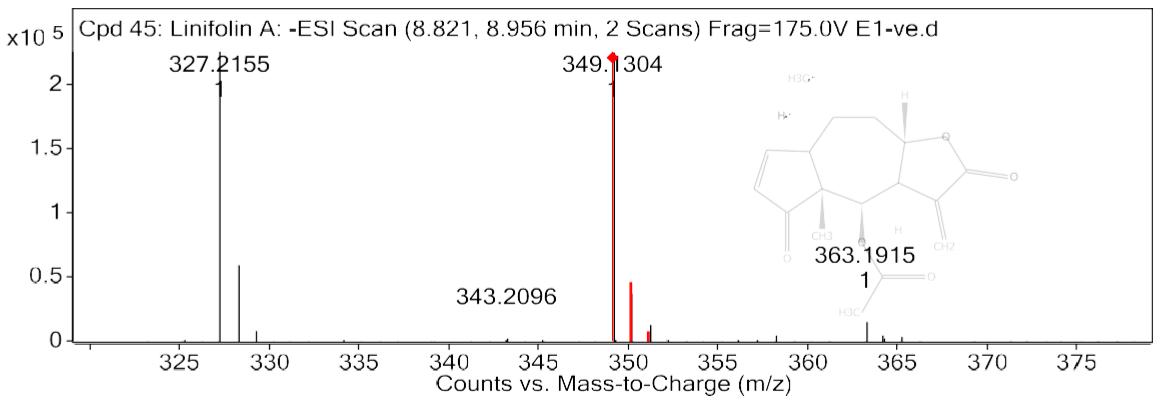

MS Spectrum Peak List

m/z	Calc m/z	Diff(ppm)	z	Abund	Formula	Ion
197.8059				28012.05		
327.2155			1	325771.69		
328.2187			1	60871.77		
349.1304	349.1293	-3.23	1	225070.3	C17 H20 O5	(M+HCOO)-
350.1335	350.1327	-2.24	1	37789.12	C17 H20 O5	(M+HCOO)-
351.13	351.135	14.21	1	13449.76	C17 H20 O5	(M+HCOO)-
583.0012			1	45268.63		
591.1068			1	73719.38		
593.1041			1	36442.61		
829.1118			1	30840.67		

MSMS Spectrum

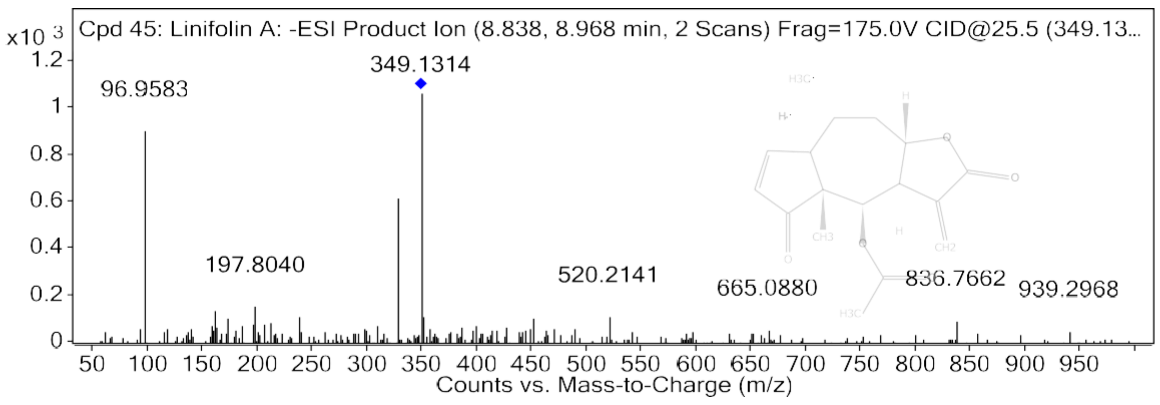

MS/MS Spectrum Peak List

m/z	z	Abund
96.9583		909.84
160.8381		138.43
197.804		159.27
237.9687		112.48
327.2156	1	618.75
328.214	1	219.61
349.1314	1	1067.71
350.1331	1	117.36
450.1935		110.06
520.2141		116.12

Compound Structure

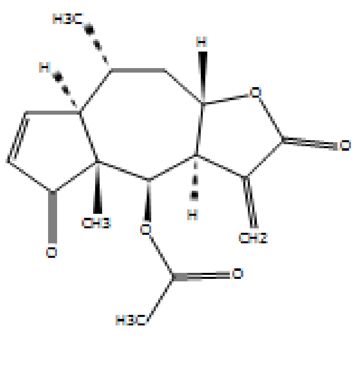

37.N2-(2-Carboxymethyl- 2-hydroxysuccinoyl)arginine

Compound Label	Name	m/z	RT	Algorithm	Mass
Cpd 52: N2-(2-Carboxymethyl-2-hydroxysuccinoyl)arginine	**N2-(2-Carboxymethyl-2-hydroxysuccinoyl)arginine**	393.1286	10.612	Auto MS/MS	348.1297

MS Spectrum

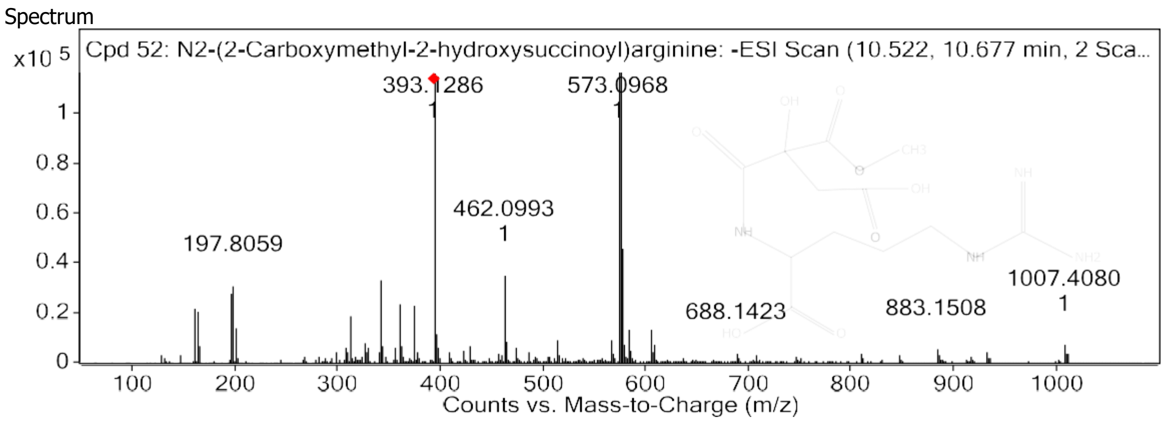

MS Zoomed Spectrum

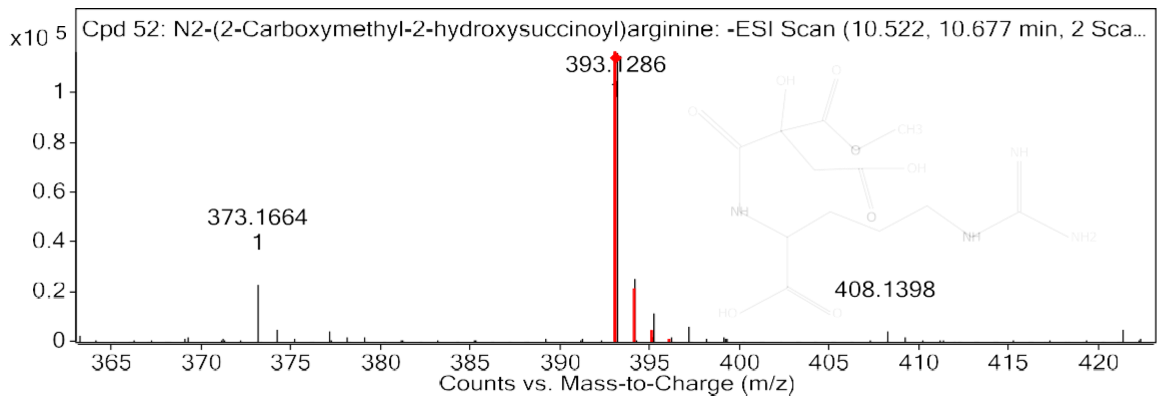

MS Spectrum Peak List

m/z	Calc m/z	Diff(ppm)	z	Abund	Formula	Ion
341.1944			1	33673.26		
393.1286	393.1263	-5.76	1	116310.91	C12 H20 N4 O8	(M+HCOO)-
394.1315	394.1292	-5.95	1	26075.67	C12 H20 N4 O8	(M+HCOO)-
395.1269	395.1311	10.57	1	12260.87	C12 H20 N4 O8	(M+HCOO)-
396.1274	396.1336	15.61	1	2636.49	C12 H20 N4 O8	(M+HCOO)-
462.0993			1	35513.1		
573.0968			1	368510.19		
574.0994			1	98120.02		
575.0938			1	188124		
576.0958			1	46116.3		

MSMS Spectrum

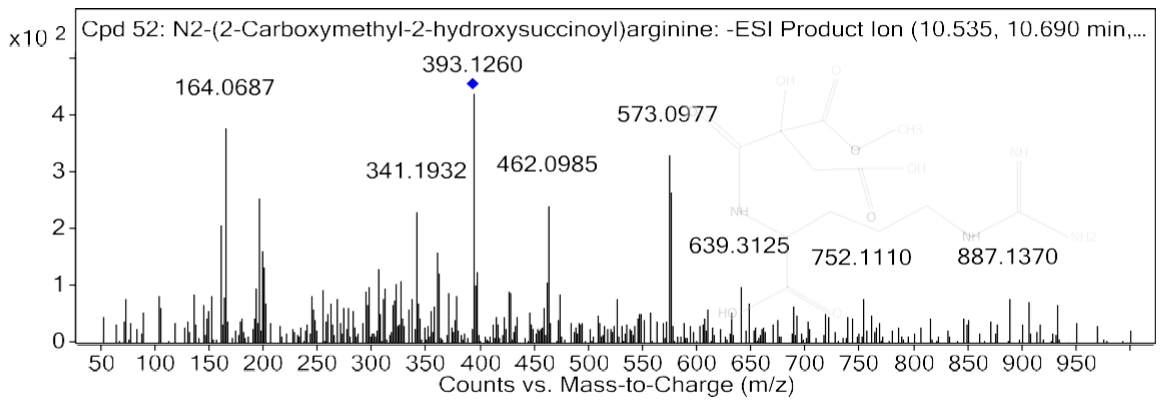

MS/MS Spectrum Peak List

m/z	z	Abund
160.8395		208.44
164.0687		377.53
195.8089		253.91
197.8066		162.37
341.1932	1	230.86
393.126	1	439.9
462.0985		242.45
573.0977	1	330.4
574.0958	1	161.19
575.0922	1	266.4

Compound Structure

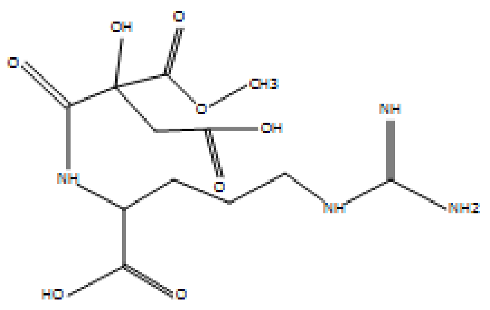

38.9Z-Octadecenedioic acid

Compound Label	Name	m/z	RT	Algorithm	Mass
Cpd 54: 9Z-Octadecenedioic acid	**9Z-Octadecenedioic acid**	311.2204	11.488	Auto MS/MS	312.2278

MS Spectrum

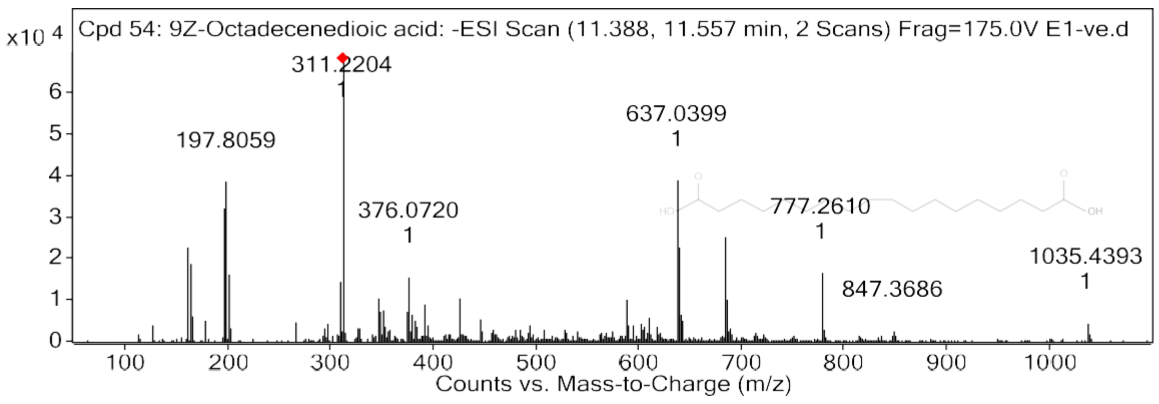

MS Zoomed Spectrum

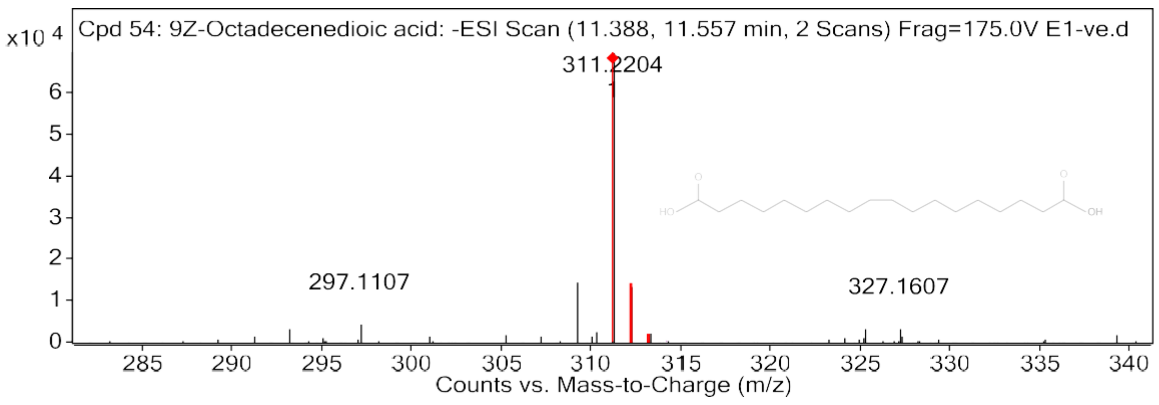

MS Spectrum Peak List

m/z	Calc m/z	Diff(ppm)	z	Abund	Formula	Ion
160.8402				22955.96		
162.8371				19055.45		
195.8091				32525.24		
197.8059				38851.21		
311.2204	311.2228	7.75	1	69805.48	C18 H32 O4	(M-H)-
312.2237	312.2262	8.14	1	13551.76	C18 H32 O4	(M-H)-
313.2324	313.2288	-11.4	1	2660.82	C18 H32 O4	(M-H)-
637.0399			1	39222.22		
639.0373			1	23161.78		
683.302			1	25562.79		

MSMS Spectrum

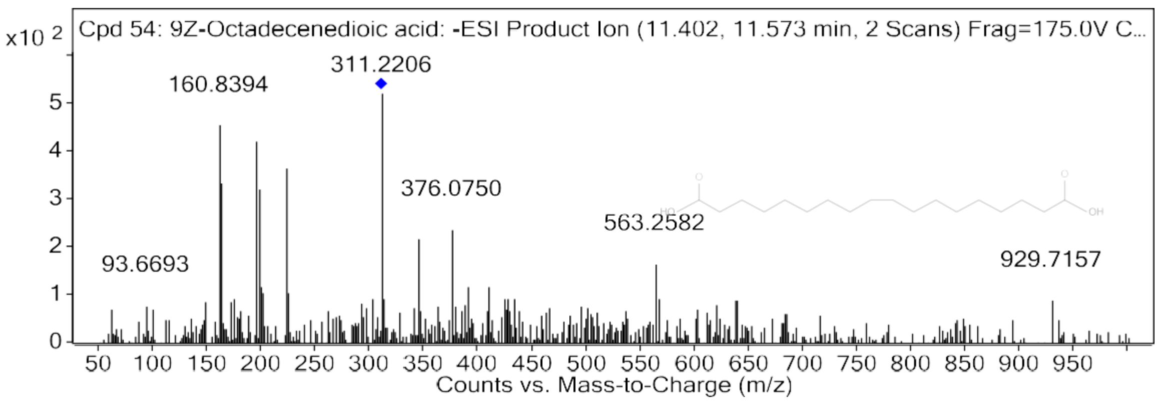

MS/MS Spectrum Peak List

m/z	z	Abund
160.8394	1	454.39
162.8356	1	332.82
195.8088		422.01
197.8047		320.16
223.1679	1	366.05
311.2206	1	521.87
345.1819		219.87
376.075		237.24
391.1796		118.57
563.2582		165.66

Compound Structure

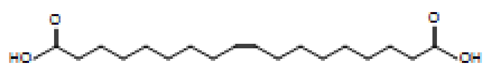

39.Trilobolide

Compound Label	Name	*m/z*	RT	Algorithm	Mass
Cpd 55: Trilobolide	**Trilobolide**	521.2495	12.359	Auto MS/MS	522.2548

MS Spectrum

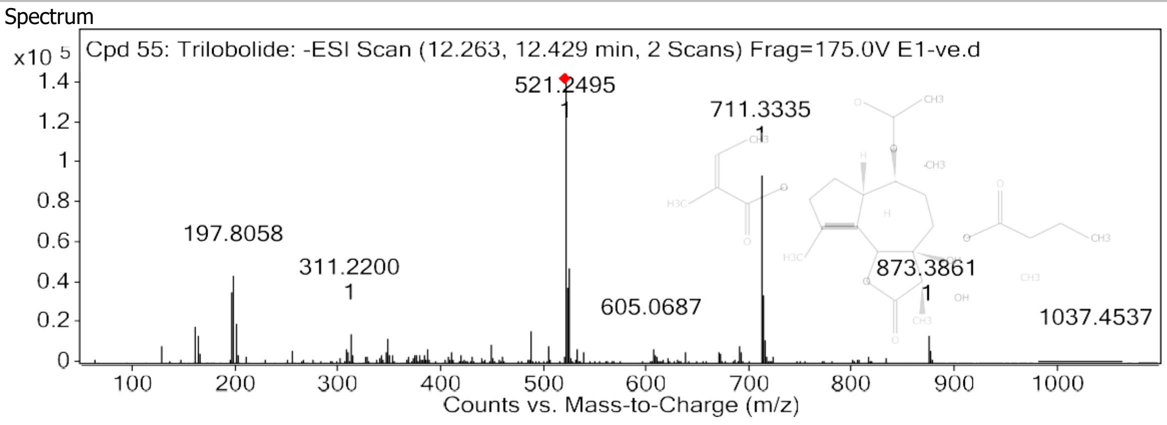

MS Zoomed Spectrum

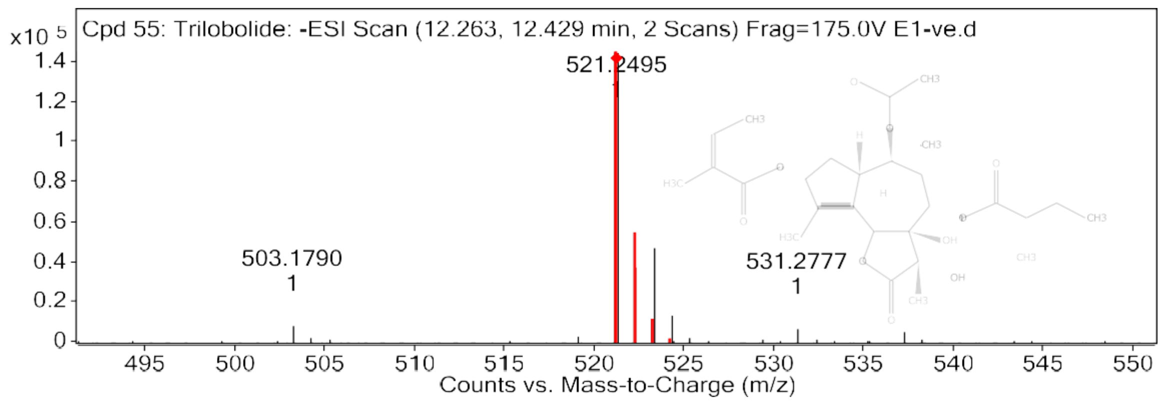

MS Spectrum Peak List

m/z	Calc m/z	Diff(ppm)	z	Abund	Formula	Ion
195.8089				36085.02		
197.8058				44332.73		
521.2495	521.2392	-19.74	1	145147.67	C27 H38 O10	(M-H)-
522.2527	522.2426	-19.34	1	38416.74	C27 H38 O10	(M-H)-
523.2478	523.2452	-4.89	1	47988.5	C27 H38 O10	(M-H)-
524.2507	524.2479	-5.36	1	13919.21	C27 H38 O10	(M-H)-
525.2527	525.2504	-4.24	1	3210	C27 H38 O10	(M-H)-
711.3335			1	94000.06		
712.3367			1	32261.55		
713.3326			1	34625.62		

MSMS Spectrum

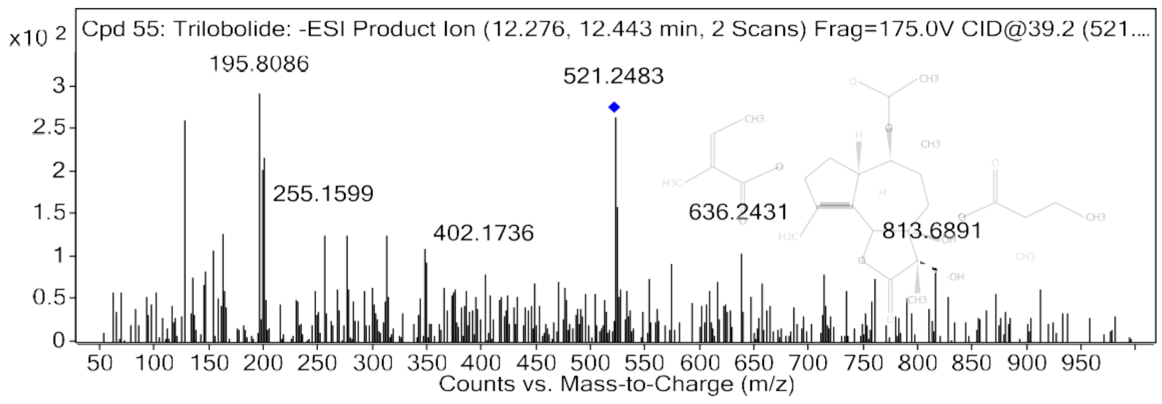

MS/MS Spectrum Peak List

m/z	z	Abund
126.9026		261.83
162.837		128.12
195.8086		293.54
197.8051		204
199.8036		217.77
255.1599		126.36
275.1985		125.54
312.5617		126.1
521.2483	1	264.92
523.2447	1	160.13

Compound Structure

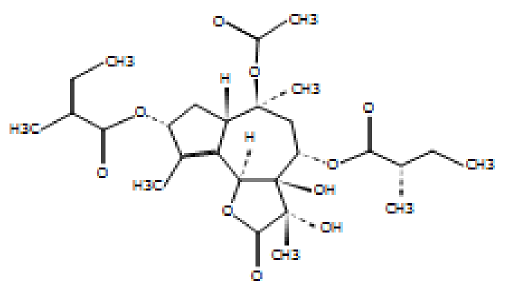

40.Thalidasine

Compound Label	Name	*m/z*	RT	Algorithm	Mass
Cpd 56: Thalidasine	**Thalidasine**	711.3339	12.591	Auto MS/MS	652.3179

MS Spectrum

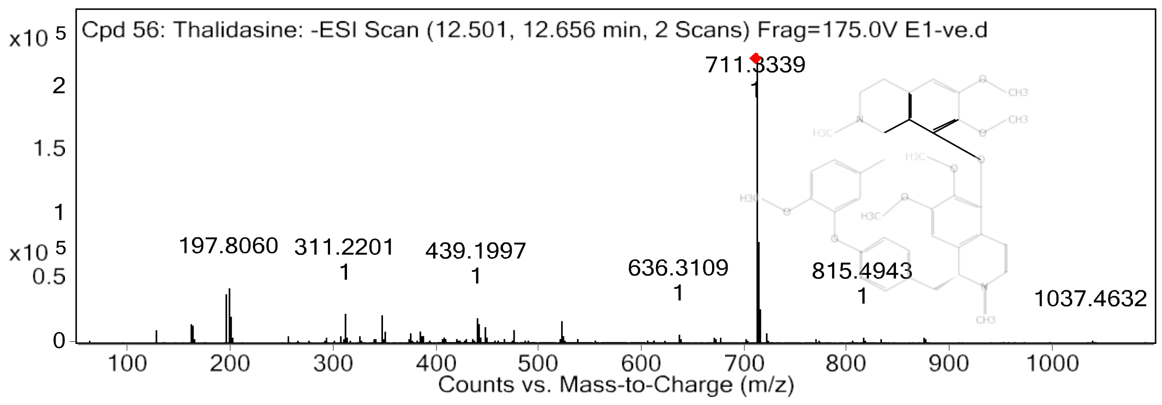

MS Zoomed Spectrum

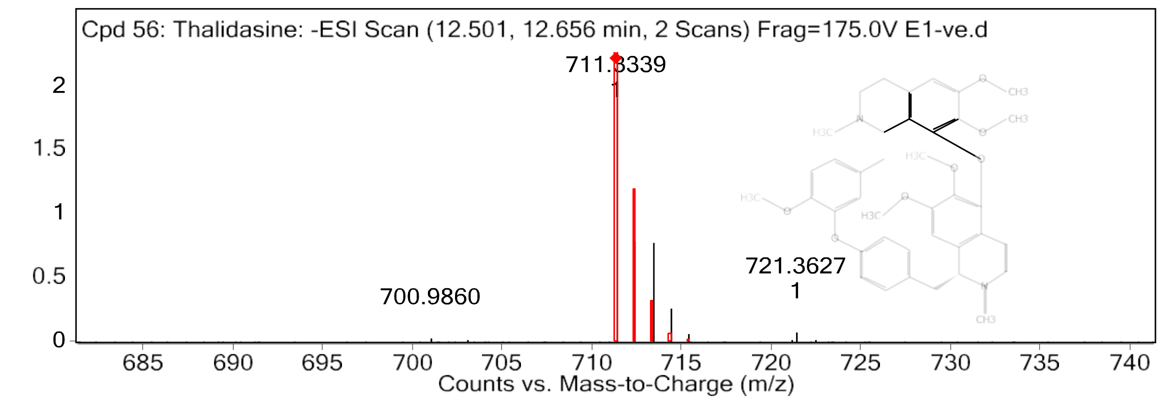

MS Spectrum Peak List

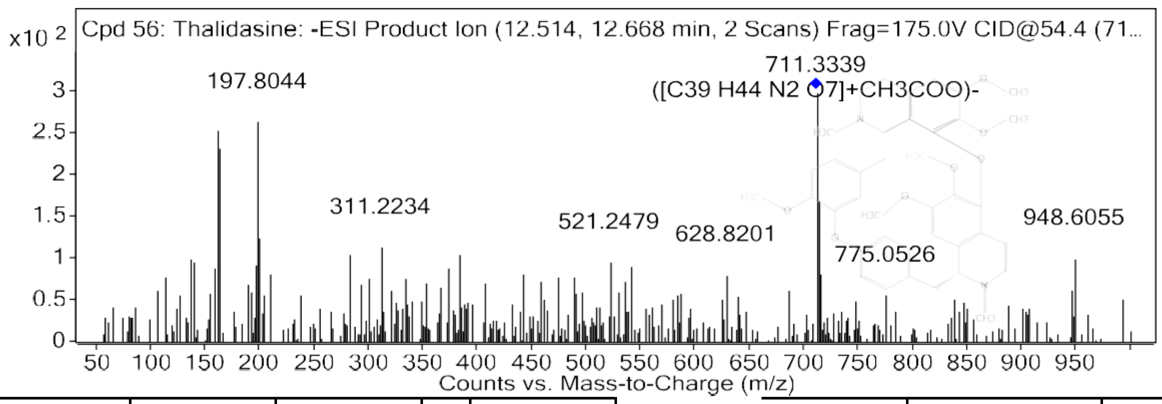

m/z	Calc m/z	Diff(ppm)	z	Abund	Formula	Ion
195.8088				38586.21		
197.806				43683.27		
199.8028				20773.66		
311.2201			1	22966.23		
347.1969			1	21853.04		
711.3339	711.3287	-7.23	1	226267.45	C39 H44 N2 O7	(M+CH3COO)-
712.3368	712.332	-6.8	1	79616	C39 H44 N2 O7	(M+CH3COO)-
713.3329	713.3349	2.9	1	78079.98	C39 H44 N2 O7	(M+CH3COO)-
714.3344	714.3377	4.69	1	27384.85	C39 H44 N2 O7	(M+CH3COO)-
715.3391	715.3405	1.95	1	6524.22	C39 H44 N2 O7	(M+CH3COO)-

MSMS Spectrum

MS/MS Spectrum Peak List

m/z	Calc m/z	Diff (ppm)	z	Abund	Formula	Ion
160.8399				254.03		
162.8355				232.65		
197.8044				263.73		
199.8018				125.15		
283.2629				104.83		
311.2234				113.57		
383.1716				105.5		
711.3339	711.3287	-7.33		298.85	C39 H44 N2 O7	(M+CH3COO)-
712.3367			1	255.07		
713.3417			1	169.51		

Compound Structure

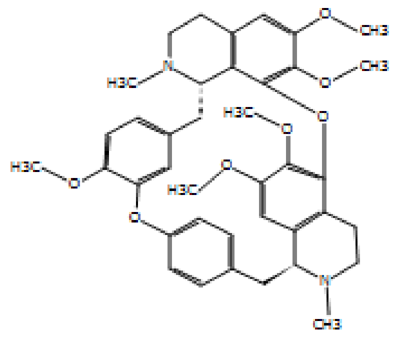

41.α-Linolenic Acid

Compound Label	Name	m/z	RT	Algorithm	Mass
Cpd 70: α-Linolenic Acid	**α-Linolenic Acid**	277.2151	16.479	Auto MS/MS	278.2223

MS Spectrum

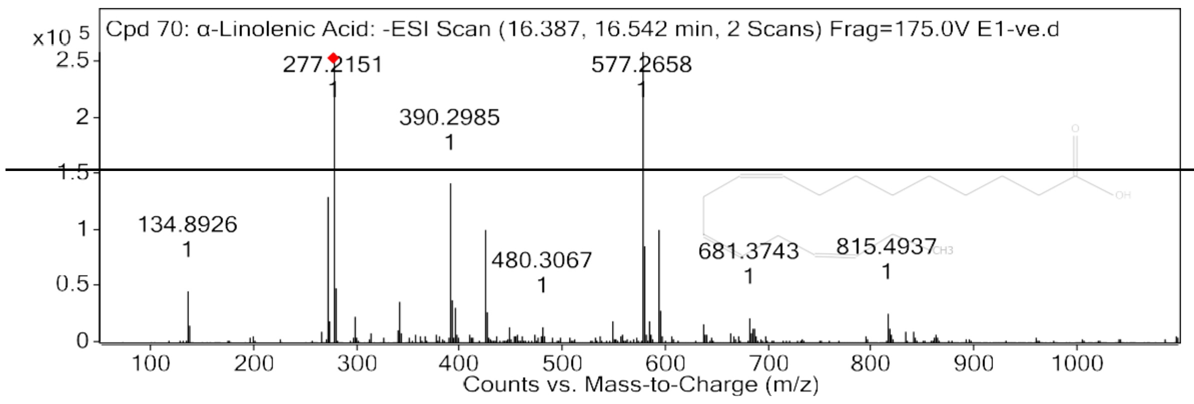

MS Zoomed Spectrum

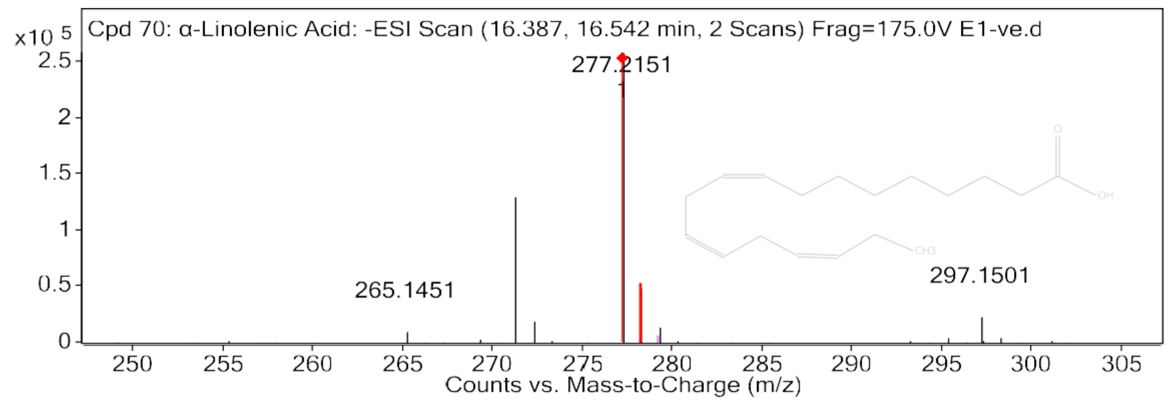

MS Spectrum Peak List

m/z	Calc m/z	Diff(ppm)	z	Abund	Formula	Ion
134.8926			1	46314.64		
271.2256			1	129954.75		
277.2151	277.2173	7.9	1	257980.06	C18 H30 O2	(M-H)-
278.2182	278.2207	9.17	1	49375.91	C18 H30 O2	(M-H)-
389.2439			1	54277.47		
390.2985			1	141770.44		
424.2827			1	101499.24		
577.2658			1	284047.13		
578.269			1	86347.5		
593.27			1	101616.77		

MSMS Spectrum

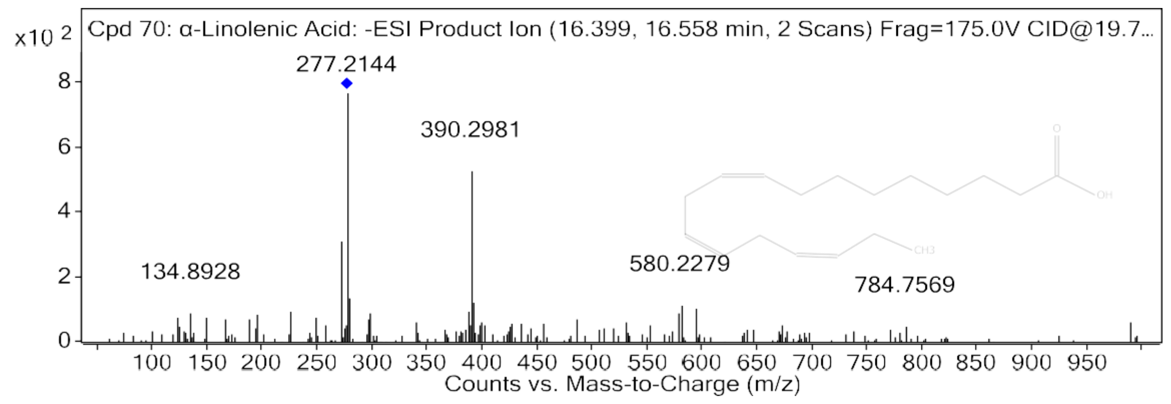

MS/MS Spectrum Peak List

m/z	z	Abund
225.2202		94.55
271.2266	1	314.39
277.2144	1	769.84
278.2162	1	124.57
279.2291		138.39
387.6397		95.94
390.2981	1	529.95
391.3039	1	122.57
580.2279		115.86
593.2618		104.77

Compound Structure

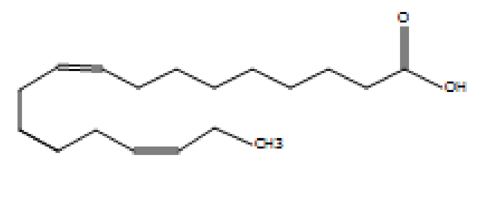

42.16-Hydroxy hexadecanoic acid

Compound Label	Name	*m/z*	RT	Algorithm	Mass
Cpd 71: 16-Hydroxy hexadecanoic acid	**16-Hydroxy hexadecanoic acid**	271.2256	16.487	Auto MS/MS	272.2328

MS Spectrum

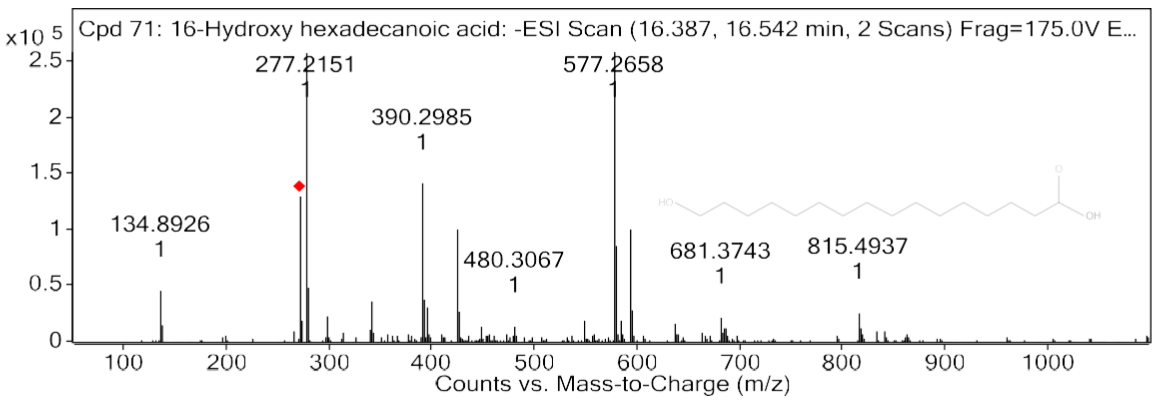

MS Zoomed Spectrum

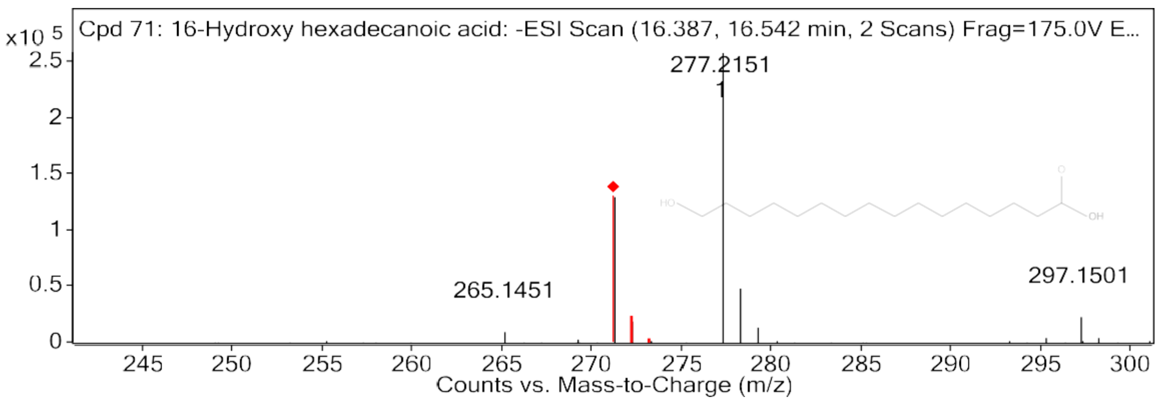

MS Spectrum Peak List

m/z	Calc m/z	Diff(ppm)	z	Abund	Formula	Ion
271.2256	271.2279	8.34	1	129954.75	C16 H32 O3	(M-H)-
272.2286	272.2313	9.85	1	19627.7	C16 H32 O3	(M-H)-
273.2315	273.234	9.09	1	2900.26	C16 H32 O3	(M-H)-
277.2151			1	257980.06		
389.2439			1	54277.47		
390.2985			1	141770.44		
424.2827			1	101499.24		
577.2658			1	284047.13		
578.269			1	86347.5		
593.27			1	101616.77		

MSMS Spectrum

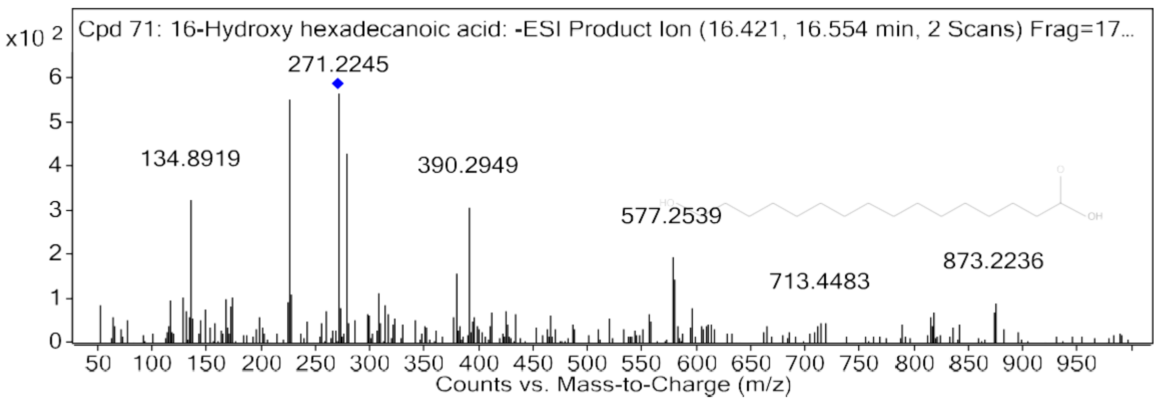

MS/MS Spectrum Peak List

m/z	z	Abund
134.8919		325.8
225.221	1	553.72
226.2219	1	112.36
271.2245	1	568.44
277.2134	1	430.13
307.1712		114.16
379.218		159.66
390.2949		310.79
577.2539		195.57
578.2731		146.9

Compound Structure

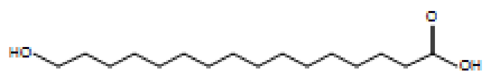

43.4-(3-Hydroxy-7-phenyl- 6-heptenyl)-1,2- benzenediol

Compound Label	Name	*m/z*	RT	Algorithm	Mass
Cpd 75: 4-(3-Hydroxy-7-phenyl-6-heptenyl)-1,2-benzenediol	**4-(3-Hydroxy-7-phenyl-6-heptenyl)-1,2-benzenediol**	297.1505	17.381	Auto MS/MS	298.1575

MS Spectrum

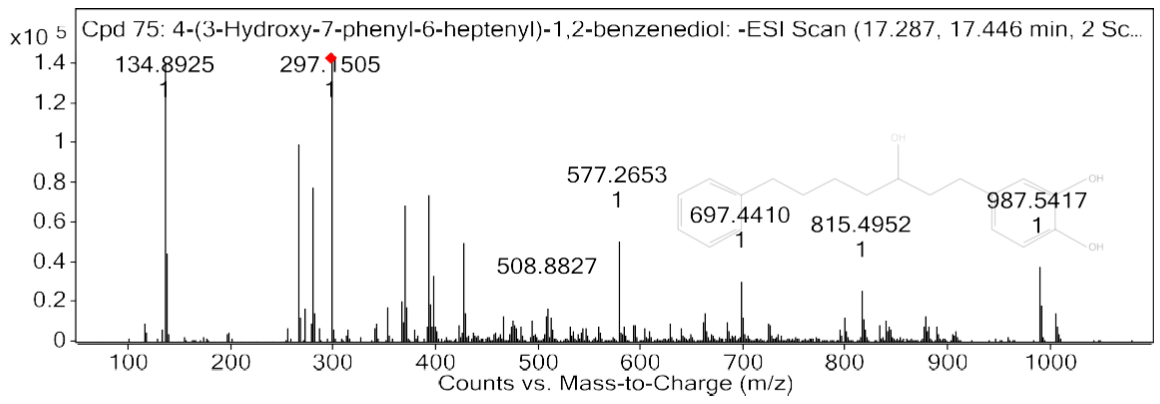

MS Zoomed Spectrum

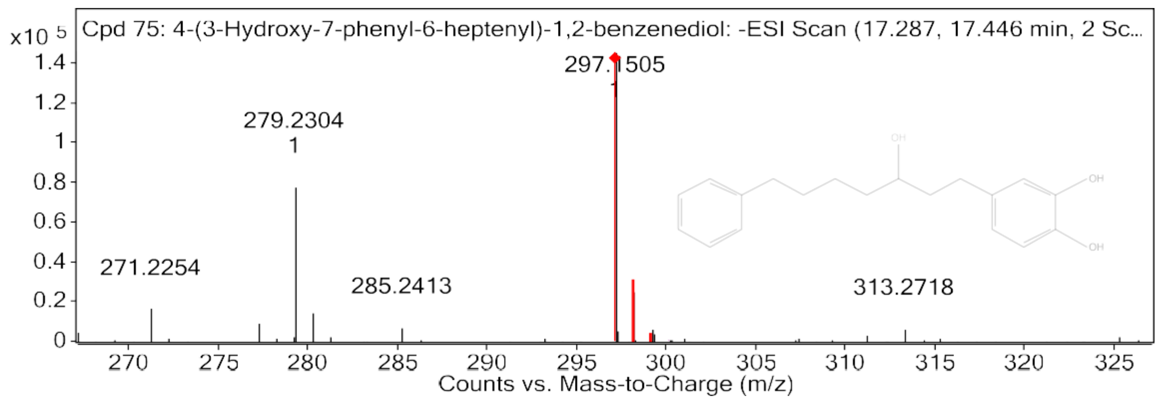

MS Spectrum Peak List

m/z	Calc m/z	Diff(ppm)	z	Abund	Formula	Ion
134.8925			1	140829.13		
265.1454			1	99959.23		
279.2304			1	78788.25		
297.1505	297.1496	-2.89	1	146295.66	C19 H22 O3	(M-H)-
298.1538	298.153	-2.68	1	25605.02	C19 H22 O3	(M-H)-
299.1496	299.1558	20.86	1	6935.89	C19 H22 O3	(M-H)-
369.2983			1	69205.64		
392.3142			1	74955.22		
426.2982			1	50642.21		
577.2653			1	51199.57		

MSMS Spectrum

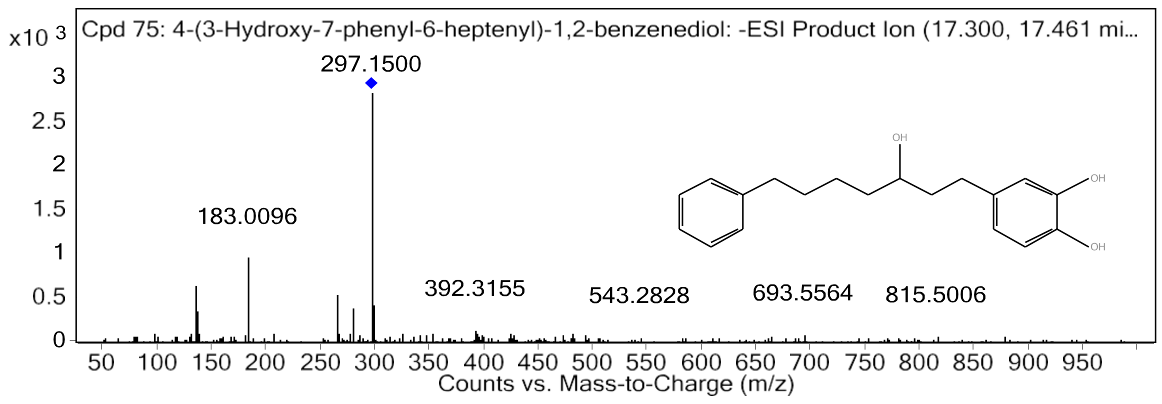

MS/MS Spectrum Peak List

m/z	z	Abund
134.8928	1	644.42
136.8894	1	361.95
138.8885		109.84
183.0096	1	961.88
184.0184	1	114.67
265.1427	2	548.47
279.2287		397.34
297.15	1	2839.11
298.1548	1	427.45
392.3155		139.27

Compound Structure

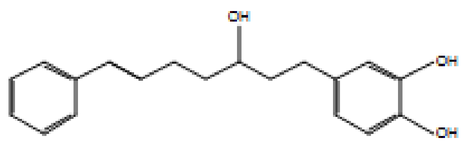

44.(6beta,8betaOH)-6,8-Dihydroxy-7(11)- eremophilen-12,8- olide

Compound Label	Name	*m/z*	RT	Algorithm	Mass
Cpd 76: (6beta,8betaOH)-6,8-Dihydroxy-7(11)-eremophilen-12,8-olide	**(6beta,8betaOH)-6,8-Dihydroxy-7(11)-eremophilen-12,8-olide**	265.1458	17.524	Auto MS/MS	266.1527

MS Spectrum

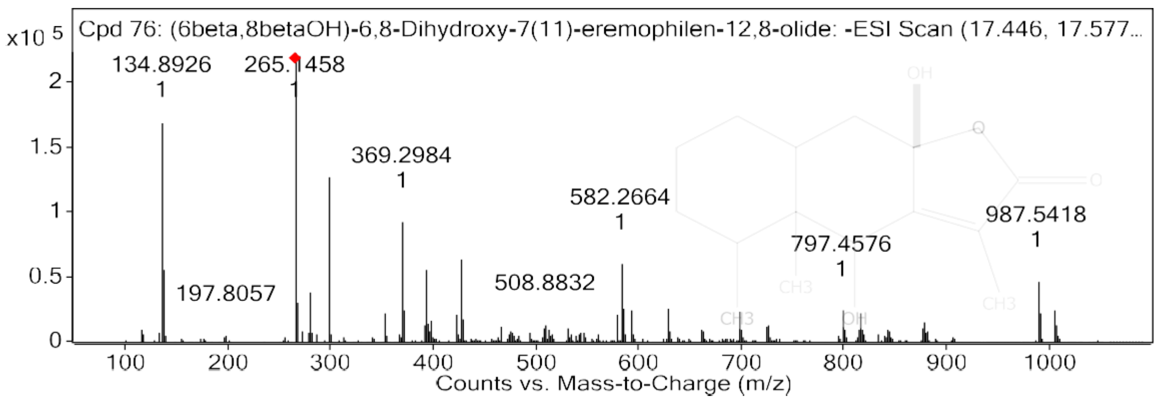

MS Zoomed Spectrum

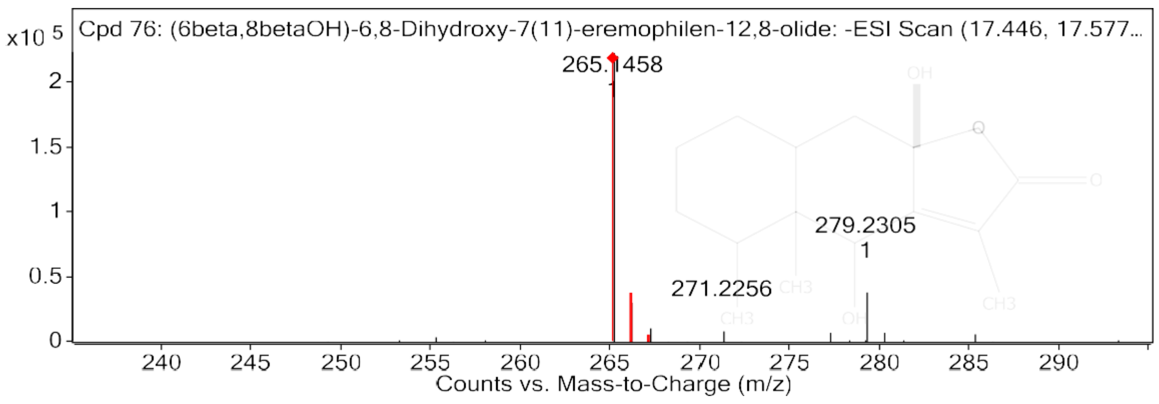

MS Spectrum Peak List

m/z	Calc m/z	Diff(ppm)	z	Abund	Formula	Ion
134.8926			1	169243.72		
136.8897			1	55966.19		
265.1458	265.1445	-4.73	1	223387.05	C15 H22 O4	(M-H)-
266.1487	266.1479	-2.83	1	30791.45	C15 H22 O4	(M-H)-
267.145	267.1504	20.18	1	11087.63	C15 H22 O4	(M-H)-
297.1505			1	127708.8		
369.2984			1	93393.69		
392.3143			1	56480.8		
426.2985			1	64310.85		
582.2664			1	61171.13		

MSMS Spectrum

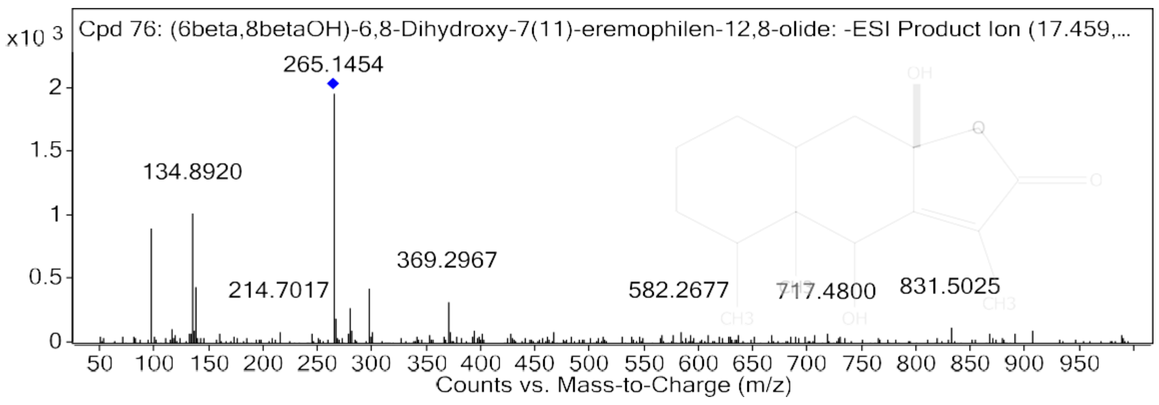

MS/MS Spectrum Peak List

m/z	z	Abund
96.9583		909.2
115.9172		120.27
134.892	1	1022.72
136.8889	1	446.57
265.1454	1	1962.41
266.1458	1	201.54
279.2306	1	282.94
297.1508		430.91
369.2967	1	328.53
831.5025		123.92

Compound Structure

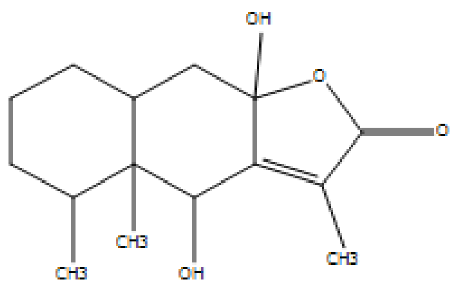

References:

1. Gulfraz, M.; Sadiq, A.; Tariq, H.; Imran, M.; Qureshi, R., Phytochemical analysis and antibacterial activity of Eruca sativa seed. *Pakistan Journal of Botany* **2011,** *43*, 1351-1359.

2.Uğur, A.; Süntar, I.; Aslan, S.; Orhan, I. E.; Kartal, M.; Sekeroğlu, N.; Eşiyok, D.; Sener, B., Variations in fatty acid compositions of the seed oil of Eruca sativa Mill. caused by different sowing periods and nitrogen forms. *Pharmacogn Mag* **2010,** *6* (24), 305-308.

3.Jirovetz, L.; Smith, D.; Buchbauer, G., Aroma compound analysis of Eruca sativa (Brassicaceae) SPME headspace leaf samples using GC, GC-MS, and olfactometry. *Journal of agricultural and food chemistry* **2002,** *50* (16), 4643-6.

4.Abdul-Jalil, T. Z. In *Phytochemicals Screening by GC / MS and Determination of Some Flavonol in Cultivated Iraqi Eruca sativa Dried Leaves Extract and its Biological Activity as Antioxidant*, 2016.

5.Villatoro-Pulido, M.; Priego-Capote, F.; Álvarez-Sánchez, B.; Saha, S.; Philo, M.; Obregón-Cano, S.; De Haro-Bailón, A.; Font, R.; Del Río-Celestino, M., An approach to the phytochemical profiling of rocket [Eruca sativa (Mill.) Thell]. *Journal of the science of food and agriculture* **2013,** *93* (15), 3809-19.

6.Michael, H.; Shafik, R.; Rasmy, G., Studies on the chemical constituents of fresh leaf of Eruca sativa extract and its biological activity as anticancer agent in vitro. *Journal of Medicinal Plants Research* **2011,** *5*, 1184-1191.

7.S.A.:, H., Phytochemical study of vsome medicinal compounds present in Hedera helix L. plant cultivated in Iraq. *M Sc. thesis Baghdad University* **2014,** *20*.

8.Blažević, I.; Mastelić, J., Free and bound volatiles of rocket (Eruca sativa Mill.). *Flavour and Fragrance Journal* **2008,** *23* (4), 278-285.
